# Review of Characteristics and Analytical Methods for Determination of Thiabendazole

**DOI:** 10.3390/molecules28093926

**Published:** 2023-05-06

**Authors:** Mateja Budetić, Doris Kopf, Andrea Dandić, Mirela Samardžić

**Affiliations:** Department of Chemistry, Josip Juraj Strossmayer University of Osijek, 31000 Osijek, Croatia; mbudetic@kemija.unios.hr (M.B.); andreajuric@kemija.unios.hr (A.D.)

**Keywords:** thiabendazole, pesticide, analytical methods

## Abstract

Thiabendazole (TBZ) is a fungicide and anthelmintic drug commonly found in food products. Due to its toxicity and potential carcinogenicity, its determination in various samples is important for public health. Different analytical methods can be used to determine the presence and concentration of TBZ in samples. Liquid chromatography (LC) and its subtypes, high-performance liquid chromatography (HPLC) and ultra-high-performance liquid chromatography (UHPLC), are the most commonly used methods for TBZ determination representing 19%, 18%, and 18% of the described methods, respectively. Surface-enhanced Raman spectroscopy (SERS) and fluorimetry are two more methods widely used for TBZ determination, representing 13% and 12% of the described methods, respectively. In this review, a number of methods for TBZ determination are described, but due to their limitations, there is a high potential for the further improvement and development of each method in order to obtain a simple, precise, and accurate method that can be used for routine analysis.

## 1. Introduction

Thiabendazole (4-1(1H-benzimidazol-2-yl)-1,3-thiazole (TBZ)) is a benzimidazole pesticide. It is also known as the food additive E233 from the class of preservatives [1]. It is an antifungal and anthelminthic drug approved by the United States Food and Drug Administration (FDA) [2]. Preharvest and post-harvest treatment of fruit and vegetables with TBZ can reduce mold, pests and rot, and deterioration during storage and transport [3]. TBZ is one of the most detected pesticides in Europe and USA [4,5]. It is known that it cannot be effectively removed from fruit by washing [6] and is stable during food processing procedures [7]. The maximum residue levels of TBZ allowed by the European Food Safety Authority is 10 mg/kg in papayas, 7 mg/kg in citrus fruit and mangoes, 6 mg/kg in bananas, 4 mg/kg in pome fruit, 3 mg/kg in sweet potatoes, 0.05 mg/kg in teas and coffee beans, less than 0.02 mg/kg in other fruit and vegetables, and less than 2 mg/kg in products of animal origin [8]. Despite the fact that TBZ possesses low toxicity with a toxicity category of 4 (least toxic), the US Environmental Protection Agency (EPA) classified it as likely to be carcinogenic at doses high enough to cause disturbance of the thyroid hormone balance [6,9]. According to EPA, the allowable daily intake (ADI) of TBZ is 0.1 mg/kg, while according to World Health Organization (WHO) it is 0.3 mg/kg. For single-dose exposure, the no observed effect level (NOEL) is 3.3 mg/kg per day [10].

In the study of Zhang and coworkers [11], the in vitro and in vivo antiproliferative activity of TBZ on murine metastatic melanoma cell line B16F10 was investigated. The obtained results revealed TBZ prevented metastatic melanoma cell proliferation by inducing apoptosis in the B16F10 cell line. Furthermore, TBZ has been repurposed as a vascular disrupting agent, which is described in the study of Zhang and coworkers. The structure of TBZ was modified for the purpose of preparing more active compounds. Two derivatives of TBZ were singled out as the most potent compounds and verified as anti-angiogenesis and vascular disrupting agents [2]. Several metal complexes of TBZ were prepared to investigate their antimicrobial activities. The results revealed that two complexes, copper and zinc, exhibited very promising antimicrobial activity against selected bacteria species [12].

A wide range of biological activities of TBZ and its presence in food are the main reasons for the precise determination and monitoring of TBZ residues in food. For that purpose, various analytical methods can be used. Some of the most important requirements in the development of these methods are simplicity, simple sample preparation, selectivity, accuracy, low limit of detection (LOD), low cost, and environmental safety. In addition, the ability to analyze a large number of samples in a short time is preferred.

This study for the first time provides a review of TBZ properties and methods for its determination developed in the period from 2000 to 2023. Therefore, it could be used as the initial step in the development of new analytical methods for determination of TBZ or in TBZ research. Based on our knowledge, there are no other review articles that describe TBZ and its determination. For this research, Scopus, Web of Science and PubMed were used as research databases.

## 2. Physicochemical Properties

TBZ is a white crystalline powder and it is stable as a solid and in solution. It has low solubility in water at neutral pH while its solubility increases in dilute acid and alkali whereby its maximum solubility is at pH 2.5 where it forms a 1.5% solution [13]. TBZ has the molecular formula C_10_H_7_N_3_S with a molecular weight of 201.25 g/mol while its melting range is 298–301 °C [14]. The physicochemical properties of TBZ are presented in Table 1.

## 3. Synthesis

**Scheme 1:**
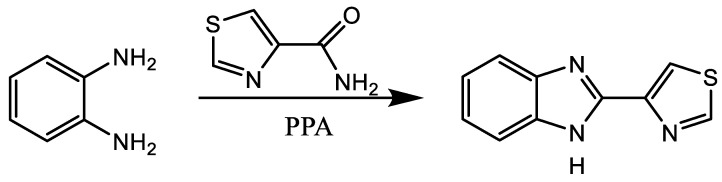
Preparation of TBZ from *o*-phenylenediamine [15].

In 1961, Brown and coworkers [15] reported TBZ synthesis by the reaction of 4-thiazolecarboxamide with *o*-phenylenediamine using polyphosphoric acid (PPA) as a catalyst. The reaction was conducted at 250 °C for three hours and resulted in a formation of TBZ with a yield of 64% (Scheme 1).

**Scheme 2:**
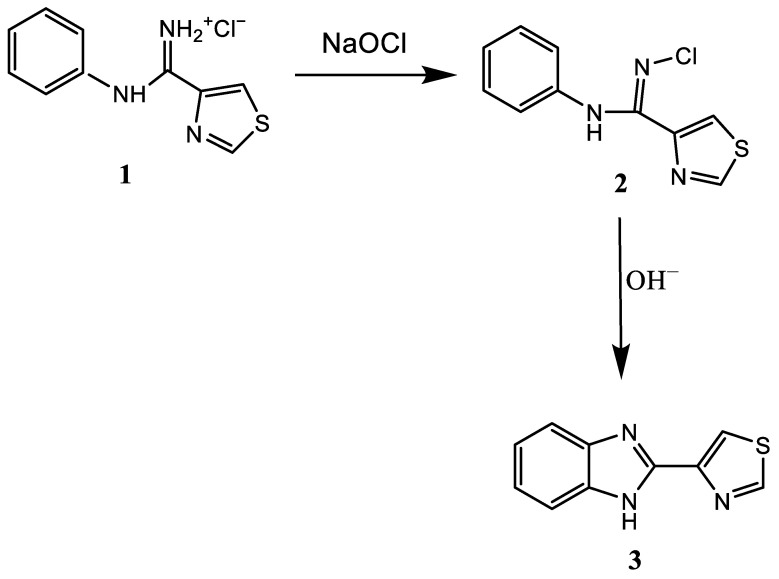
Preparation of TBZ from *N*-arylamidine hydrochloride [17].

In general, the most common synthetic pathways for TBZ synthesis include the condensation of *o*-phenylenediamine or *o*-nitroaniline with a carboxylic acid derivative whereby cyclization in both cases include a coupling at *o*-phenylene nitrogen [16]. It was found that the substituted amidines were potential precursors for benzimidazoles synthesis if the cyclization could be induced by some oxidative process. Grenda and coworkers [17] discovered that *N*-arylamidine hydrochlorides (**1**) could undergo transformation to benzimidazoles in the presence of 1 mole of sodium hypochlorite and base under mild conditions. The proposed synthetic route resulted in the formation of *N*-chloroamidine derivative (**3**) with a yield of 98% (Scheme 2).

TBZ exhibits broad spectrum anthelmintic activity without adverse toxic effects and for that reason has high commercial importance. Prior synthetic pathways for TBZ synthesis had inherent drawbacks, such as low yields, additional purifications steps, long reaction times, usage of environmentally unacceptable organic solvents, and high-pressure reaction conditions. Considering its high biological activity, various other synthetic routes aimed at producing TBZ in high yield and high purity were developed. In 1994, a new synthetic pathway was proposed which was described in a patent. The proposed synthetic pathway includes the acid-catalyzed condensation of *o*-phenylenediamine and 4-cyanothiazole in water or mixtures of water with miscible co-solvents. Advantages of this process over prior processes are high yield, high purity and a low cost one-step process. Furthermore, the advantage of this synthetic pathway is the simple isolation of TBZ by filtration of the reaction mixture due to high solubility of all components of the reaction mass, except the target product, TBZ [18].

## 4. Structural Modifications

**Figure 1:**
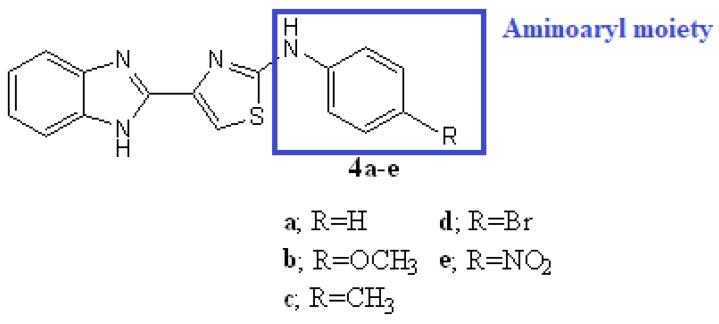
Structural formulas of target compounds **4a**–**e** [19].

The introduction of various substituents, such as alkyl, aryl, or heterocyclic units, on different positions of benzimidazole as well as thiazole nucleus, influence the biological activity of TBZ. In the research of Sood and coworkers [19], a series of TBZ derivatives were prepared by the incorporation of amino aryl moiety at position 2 of the thiazole nucleus for the purpose of preparation of safer and more potent antimicrobial agents (Figure 1). Prepared derivatives showed moderate activities against *Bacillus cereus*, *Escherichia coli*, *Yersinia enterocolitica* and *Staphylococcus aureus*. Based on the results, it was concluded that further structural modifications are needed for the improvement of antimicrobial properties of TBZ.

**Figure 2:**
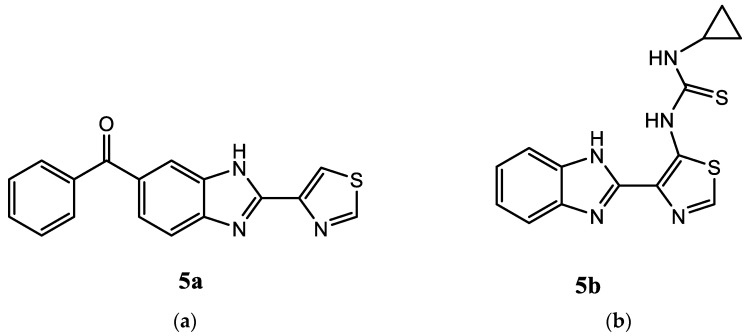
Structural formula of the most potent compounds: (**a**) **5a**; (**b**) **5b** [2].

TBZ has been in use as an anti-fungal and anthelminthic drug since 1967 but has recently been repurposed as a vascular disrupting agent. Zhang and coworkers [2] prepared a series of 24 compounds based on a TBZ scaffold. Target compounds were synthesized by condensations of *o*-phenyldiamine derivatives with thiazole-4-aldehyde, pydrine-2-aldehyde, or 4-formylthiazol-5-yl carbamate. Reactions were catalyzed by sodium pyrosulfite in dimethylformamide (DMF) as a solvent at 120 °C. All prepared compounds were tested against the growth of four different cell lines (A549, HCT-116, HepG2, and HUVECs). Two candidates, compounds **5a** and **5b**, were singled out as the most potent compounds (Figure 2) which exhibited moderate, inhibitory cell-proliferation activity and were verified as antiangiogenesis and vascular disrupting agents.

## 5. Mechanism of Action

TBZ exhibits a broad spectrum of biological activity including the primary described anthelmintic properties [15] and the later discovered fungicidal properties [20]. The precise mode of action of TBZ is unknown although there are some assumptions about the potential mechanism of action which includes inhibition of the energy metabolism and influence on glucose uptake or inhibition of the polymerization of tubulins to microtubules [21,22,23].

Benzimidazoles, in general, interfere with the energy pathway of the helminths and cause inhibition of cytoplasmic and mitochondrial malate dehydrogenase (MDH). As anthelmintics, they have the role of lipid soluble proton conductors in artificial (phospholipid bilayer) and natural (rat liver mitochondria) membrane systems according to the research of McCracken and Stilwell [24]. In addition, this class of compounds severely disturbs the transmembrane proton gradient, which allows a considerable drop in cellular ATP levels.

**Figure 3:**
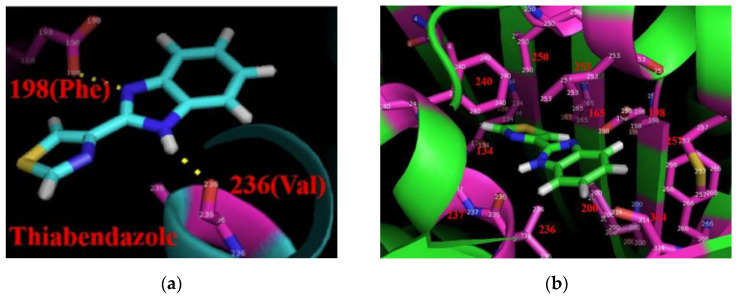
(**a**) The molecular docking between β2-tubulin protein (PDB ID: 5CA1) and TBZ; (**b**) The binding pocket of TBZ and β2-tubulin protein [26].

Tubulin is a protein necessary for cell division and also an important component of the cytoskeleton of all living cells [21,25]. The most important, among the tubulin class, are α- and β-tubulins that polymerize during cell division which results in microtubules formation. The main role of microtubules, during mitosis, is to manipulate and separate daughter chromosomes through the rapid assembly and disassembly of microtubules which presents the key function needed for cellular replication [25]. L. C. Davidse and coworkers [20] revealed that TBZ, at *c* = 80 µM, completely inhibits mitosis in the hyphae of *Aspergillus nidulans*, growing in liquid culture. Furthermore, TBZ competitively inhibits ^14^C-carbendazim binding to tubulin. Based on these results, it is assumed that the interference of TBZ with microtubule assembly is the ground for antimitotic activity of TBZ [25]. The molecular docking between β2-tubulin protein (PDB ID: 5CA1) and TBZ, and the binding pocket of TBZ and β2-tubulin protein are presented in Figure 3, respectively [26].

TBZ is also a chelating agent, which enables its use in the treatment of diseases caused by metal overload [27]. C. Marzano and coworkers [28] prepared four complexes of TBZ with copper (II) acetate, chloride, nitrate, and butanedioate. The results revealed that a complex of TBZ with copper (II) nitrate (Cu(HTBZ)_2_(NO_3_)_2_) showed activity against the human squamous carcinoma tongue-cell line (CAL-27) and the malignant melanoma skin-cell line (SK-MEL-31). Based on these results, it was concluded that the chemotherapeutic potential of TBZ increased upon metal coordination.

## 6. Pharmacokinetics

**Scheme 3:**
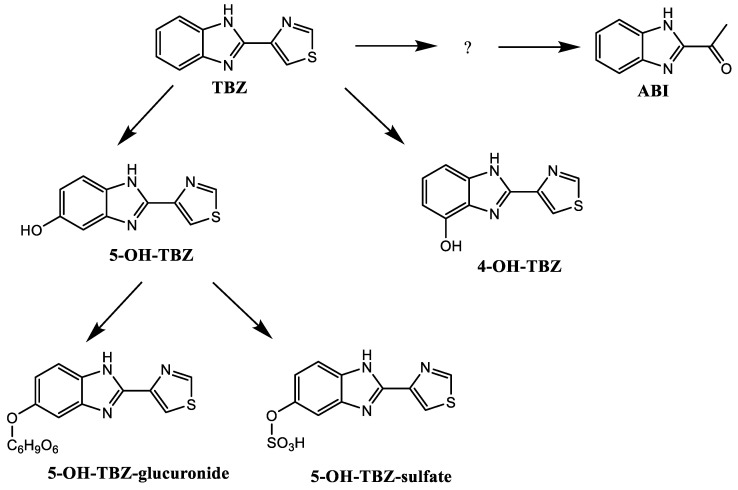
Metabolic pathway of TBZ in the mouse and rat [30].

TBZ, as a broad spectrum anthelmintic affecting gastrointestinal parasites of various domestic species (sheep, cattle, goats, and swine), is rapidly absorbed, metabolized and excreted in the mentioned animals [29]. Peak plasma concentrations were reached 2–8 h after treatment and 85% of the applied drug appeared in the urine (61%) and feces (24%) in 24 h. The metabolic pathway of TBZ was examined on animals (Scheme 3) and humans. This metabolic pathway includes benzimidazole ring hydroxylation which results in the formation of 4-hydroxythiabendazole (4-OH-TBZ) and 5-hydroxythiabendazole (5-OH-TBZ). Furthermore, together with 4-OH-TBZ and 5-OH-TBZ, 2-acetylbenzimidazole (ABI) was found in the embryo in vivo and in vitro. These results suggest that the most of TBZ metabolized to 5-OH-TBZ and its glucuronide (5-OH-TBZ-glucuronide) and sulfate (5-OH-TBZ-sulfate) in the animal species, except in humans and dogs. In this case, results suggest that there was an unidentified compound (?) which was concluded on the basis of its contribution to the difference between the radioactivity and chemical analysis of TBZ and 5-OH-TBZ [30].

Tocco and coworkers [29] conducted a study of the metabolism of TBZ in men and laboratory animals. In humans, radiometric and fluorometric assays showed peak plasma levels 1–2 h after oral treatment of 1 g of TBZ labeled with ^14^C. In addition to the prompt appearance of TBZ and its metabolites in the plasma, large quantities of labeled TBZ were excreted in urine. More than 40% of the applied drug and its metabolites were excreted within the first 4 h, and around 80% within the first 24 h. Approximately 50% of the labeled TBZ in the urine were compounds the concentrations of which could be determined by the chemical assay procedure, while less than 1% of the applied dose was excreted as unchanged TBZ or in the 5-hydroxy form. The biggest part of the applied drug was found in the urine as glucuronide (25%) and as the sulfate ester of 5-OH-TBZ (13%).

Concentrations of TBZ and its metabolites in rats were determined in the same manner as in humans. When administered, the dose was less than 100 mg/kg, approximately 92% of the applied drug was detected in the urine (66%) and feces (26%), while treatment with a dose of 100 mg/kg resulted in the appearance of 79% of the applied drug in the urine (49%) and feces (30%). Paper chromatography of 24-h urine from one of the rats treated with TBZ (25 mg/kg) revealed four radioactive spots which were identified as unchanged TBZ (3%), free 5-OH-TBZ (4%), the sulfate conjugate, 5-OH-TBZ-sulfate (39%), and the glucuronide conjugate, 5-OH-TBZ-glucuronide (28%).

## 7. Adverse Effects and Drug Interactions

TBZ is a fungicide and parasiticide and has low acute toxicity via the oral and dermal routes [10]. Possible side effects of TBZ are appetite loss, bad urine odor, diarrhea, dizziness, drowsiness, dry eyes, headache, indigestion, irritability, nausea, a sensation of floating, stomach upset or pain, tiredness, vomiting and weakness. In addition, administration of TBZ can cause severe allergic reactions, blood in the urine, blurred vision, chills, collapse, confusion, dark urine, depression, flushing, enlarged lymph nodes, hoarseness, increased thirst or urination, loss of coordination, numbness, swollen, ringing in the ears, fever, uncontrolled urination, and yellowing of the skin or eyes [31].

Abacavir is an antiviral nucleoside reverse-transcriptase inhibitor used to treat HIV and AIDS [32]. TBZ can decrease the excretion rate of Abacavir which as a consequence has a higher serum level [27]. Abametapir is a pediculicidal metalloproteinase inhibitor used in the treatment of head lice infestation [33]. The combination of TBZ with Abametapir can cause increased concentration of TBZ in serum [27]. Taking TBZ with acetylsalicylic acid (aspirin), as a drug used to treat pain and fever [34], may decrease the excretion rate of TBZ which could result in a higher serum level.

The analgesic effect of TBZ was evaluated by comparing its effects with those of morphine and aspirin. A conducted study revealed that, besides its anti-inflammatory properties, TBZ has an analgesic activity which is increased by the application of aspirin, but aspirin significantly enhances its toxicity [35].

## 8. Analytical Methods for TBZ Determination 

The determination of TBZ is essential in order to ensure food safety. Additionally, it is important for the quality control of TBZ-based drugs. So far, different analytical methods have been used for its determination, but chromatographic methods are the most commonly used. However, besides low LOD, high precision, selectivity and accuracy, there are a lot of other characteristics that are very important in new method development. Environmental safety is one of them. Today, with increased environmental awareness and knowing that environmental pollution has a significant influence on human health, the use of large amounts of organic solvents should be avoided. It can be accomplished through the miniaturization of the devices for TBZ determination, which demand a reduced amount of chemicals, or by using alternative methods. Additionally, miniaturization allows the possibility of in situ measurements. At the end, the acceptable cost of the analysis is the common limiting factor in many laboratories. Considering that, the newly developed methods should be simple, with no complicated and time-consuming sample preparation, the time of the analysis should be short and the instrumentation should be available in most of the laboratories.

According to the analyzed literature, chromatographic and spectroscopic methods are the most commonly used for TBZ determination. Although this review summarizes research published in the period from 2000 to 2023, Figure 4 presents the chronological order of analytical methods for TBZ determination by year of the beginning of the development of a certain method. It can be seen that the first method developed for TBZ determination was ultraviolet and visible (UV-Vis) spectroscopy. However, over the years, there have been significant developments in other methods. 

### 8.1. UV-Vis Spectroscopy

UV-Vis spectroscopy is primarily a quantitative analytical method used to measure light absorbance across the ultraviolet and visible ranges of the electromagnetic spectrum (180–780 nm). Due to the measured absorbance, concentration of the analyte can be easily calculated using the Beer–Lambert law. UV-Vis spectroscopy is a very simple and economical method that is available in most of laboratories. However, whilst it is less sensitive compared to other techniques, such as fluorimetry, it has a broad range of applications in the analysis of various organic and inorganic analytes, which can provide preliminary data about the absorption characteristics of the analyte, as well as data about the analyte concentration.

Methods for the determination of TBZ using UV-Vis spectroscopy started to develop in 1974 [36]. That method used chloroform for the quantitative extraction of TBZ from fruit and selectivity was achieved based on the solubility of TBZ in different solvents.

In 2018, Altunay et al. [37] developed a simple, accurate, and fast spectrophotometric method for the determination of TBZ residues in fruit (grape, apple, banana, orange, lemon, and apricot) and vegetables (mushroom, cherry, tomato, green pepper, corn, and carrot) based on the selective formation of a complex between TBZ and Cu (II) ions. For the extraction of TBZ, the authors proposed ionic liquid phase microextraction. The ionic liquid 1-butyl-3-methylimidazolium hexafluorophosphate was used as the extracting solvent.

In 2021, Tuzen et al. [38] developed a method for TBZ determination using UV-Vis spectrophotometry, based on green chemistry principles. TBZ was determined in various fruit samples. The extraction method was vortex-assisted dispersive liquid–liquid microextraction (DLLME) based on four zwitterionic deep eutectic solvents prepared from betaine mixed with 2-furoic acid, phenylacetic acid, mandelic acid, and glycolic acid in different molar ratios. All measurements were carried out at the wavelength of 305 nm. The proposed method was accurate, selective, and environmentally friendly.

In the analysis of TBZ using UV-Vis spectroscopy, attention should be paid to sample preparation due to the possible matrix effect and interferents. In the described papers, the extraction of TBZ was emphasized. Although ionic liquids and eutectic solvents were used for TBZ extraction, acetonitrile was usually used in the first step of sample preparation. Careful optimizing of the working conditions can overcome limitations of this method such as impurities, interferents, pH, and temperature influence, so it could continue to develop, although it is expected that the emphasis will be on other, more sensitive and selective analytical methods.

### 8.2. Surface-Enhanced Raman Spectroscopy

The method of vibrational spectroscopy most commonly used in the determination of TBZ is surface-enhanced Raman spectroscopy (SERS). As the name implies, SERS is a surface-sensitive technique based on the enhancement of Raman scattering by molecules adsorbed on rough metal surfaces. SERS is a combination of Raman spectroscopy and nanotechnology. The low sensitivity as the major drawback of Raman spectroscopy has been solved by the introduction of nanomaterials as enhancement factors. This kind of spectroscopy can provide information of a sample content which is called a molecular “fingerprint”. The enhancement can be achieved by electromagnetic or chemical mechanisms, and the enhancement factor can be as high as 10^14^–10^15^, making the technique sensitive enough to detect single molecules under certain conditions.

Determination of TBZ using SERS began in 2013, when Muller et al. [39] demonstrated the possibility of combining near infrared spectroscopy (NIR) and SERS by using a silver colloid as an enhancing substrate with a portable mini-Raman spectrometer. The determination was performed on “bio” lemon samples. The authors showed that the SERS signal of TBZ can be detected in the NIR region. Based on the results, it was concluded that washing the fruit under tap water did not completely remove TBZ from the fruit. One year later, the same group of authors [6] characterized the spectrum of TBZ using the same technique and under the same conditions, but the samples were grapes, bananas, oranges, and lemons. Different SERS spectra were obtained for TBZ dissolved in ethanol compared to the SERS spectra for TBZ dissolved in an aqueous solution, and the spectra for TBZ dissolved in ethanol were three orders of magnitude lower than those for TBZ dissolved in aqueous solution. The final results showed that all fruit samples, except bananas, contained a concentration of TBZ 13 times higher than the permissible value.

Using a silver nanoparticle to enhance the Raman scattering, He et al. [40] detected TBZ on the surface of apples. The authors used a surface swab method to extract TBZ, followed by SERS. Surface swab methods are regularly used to obtain biological and chemical compounds. The duration of this method, including sample preparation and detection, was 10 min, which makes this method fast and simple. The obtained results showed that the LOD was sensitive enough to determine the maximum allowable concentrations of TBZ on the surface of apples (5 μg/g).

Luo et al. [41] developed a sensitive and rapid method for the determination of phosmet and TBZ in apples using SERS with gold nanoparticles as the substrate. In this study, the authors used the QuEChERS (acronym for Quick, Easy, Cheap, Effective, Rugged, and Safe) method for sample preparation to remove interfering factors from the apple extract. In the SERS spectrum, TBZ could be visually determined at a concentration of 0.02 μg/mL. The results indicated that the developed method could be successfully used for the determination of other types of hazardous chemicals in food.

In 2017, Hong et al. [42] immobilized gold nanoparticles on an ultrafiltration membrane using a suction technique and combined this technique with SERS for the determination of TBZ in oranges and TBZ solution. The gold nanoparticles were synthesized from chloroauric acid and sodium citrate. In addition to the determination of TBZ, the authors also observed the effects of the size and amount of the golden nanoparticles on SERS enhancement. The results showed that the signal intensity was proportional to the size of the golden nanoparticles. The developed method proved to be rapid, simple, and reproducible for the determination of TBZ in water and orange peels.

A year later, Feng et al. [43] synthesized molecularly imprinted polymers (MIPs) with selective affinity for TBZ and used them as sorbents for solid phase extraction (SPE) to extract TBZ from orange juice samples. Then, they added colloidal silver nanoparticles for SERS analysis and showed that such a system formed a unique MISPE-SERS chemosensor for the rapid and sensitive determination of TBZ in orange juice. The authors concluded that the proposed MISPE-SERS chemosensor could also be used for multiple analyses, as it can be modified by synthesizing MIPs to selectively and specifically determine different analytes.

In the same year, Lin et al. [44] combined chemometric methods with the SERS method to determine TBZ in rapeseed leaves. For the experiment, the authors prepared 101 TBZ solutions with different concentrations, which they sprayed on rapeseed leaves. One of the problems was the fluorescent effect of the chlorophyl, fat, carbohydrates, and other substances, but the authors solved that possible interference by adding Fe_3_O_4_ nanoparticles.

Wang et al. [45] demonstrated the in situ detection of TBZ on orange peels using tattoo paper coated with gold island film (GIF). The authors coated the aqueous tattoo paper with gold nanoparticles in argon plasma. SERS spectra showed that the fingerprint signals were strong and sharp with no background fluorescence when this tattoo paper was used. The LOD for this method is lower than the maximum residue level for TBZ, so this method could be used for the determination of pesticides in fruit.

Using gold nanoparticles, Nie et al. [46] enhanced the SERS signal and determined TBZ quantitatively in red soil extracts. Peak intensities were observed at different wavenumbers and compared based on linear correlation and quantification limits. The authors applied density functional theory (DFT) to calculate TBZ molecules, optimized its structure, and found its characteristic peaks. The whole determination process, from sample preparation to detection, lasted 25 min, which, in addition to its good linearity and low LOD, made this method applicable for the analysis of pesticides in red soil extracts.

Alsammarraie et al. [3] proposed the determination of TBZ in lemon, carrot, and mango juice using the gold nanorod-enriched SERS. In the real samples of all three juices, the TBZ concentration was 50 μg/L, which is below the maximum residue limit of 10 mg/L. The authors demonstrated that the developed method for TBZ detection in juice samples is efficient, fast (10 min for sample preparation and SERS measurement), and convenient.

In 2019, Kang et al. [47] combined the nanoparticle metastable state method SERS and thin layer chromatography (TLC-MSNERS) to determine TBZ in a mixture of three pesticides on cherry skin. To enhance Raman scattering, they used polyurethane-Ag nanoparticles. The TLC was used to separate the different pesticides and then perform the determination using the SERS method. Knowing that the HPLC method is the traditional method for the determination of pesticides, the authors compared the data obtained with those obtained using HPLC. The deviations in the measurement of pesticides concentration were less than 10% between compared methods, but the developed method was faster and cheaper than the HPLC method; therefore, it could be a very good choice for the rapid simultaneous detection of different pesticides.

Fu et al. [48] measured TBZ in the TBZ methanol solution and in apples. Enhancing the Raman scattering was performed using homogeneous and reusable gold nanorods. The results showed a logarithmic correlation between TBZ concentration and Raman scattering intensity. The emphasis was placed on the fact that the gold nanorod array substrate can be used more than seven times in SERS detection.

Xuan et al. [49] developed an Ag-Au-IP6-Mil-101(Fe) substrate for the SERS method by reducing silver and gold nanoparticles in situ to form a metal–organic framework (MOF) of terephthalic acid (PTA) and Fe^3+^, named Mil-101(Fe), modified with inositol hexaphosphate (IP6). MOFs are compounds prepared from metal ions and organic ligands that form a special class of porous materials with nanoscale pores. The authors determined TBZ in peach juice samples. The LOD for TBZ obtained using the proposed method was 50 ppb. The authors also performed the HPLC method to verify the reliability of the synthesized MOFs, and the results were in agreement with those obtained using the SERS method.

Mekonnen et al. [50] developed a highly active SERS flexible plasmonic paper substrate by composing Ag@SiO_2_ nanocubes with Fe-TiO_2_ nanosheets on modified miniaturized paper to determine TBZ in a standard TBZ solution, on the surface of apples, and in apple juice matrix. The novel substrate showed great sensitivity to TBZ, and the results showed that with increasing TBZ concentration, the characteristic Raman peaks became more intense.

Teixeira and Poppi [51] determined TBZ and carbaryl in mango peel samples. The gold nanoparticles were deposited on plain paper using a wax printer, which superficially enhanced the intensity of Raman scattering. They used the sodium thiocyanate as the internal standard to normalize the signal, thus reducing the relative standard deviation (RSD) from 10.0% to 2.3%. After the measurements, they noticed that there was a very good distinction between the solutions containing less than 2 ppm of TBZ and the solutions with higher concentration. All the results were confirmed using HPLC with diode array detection (DAD).

In several juice samples (apple, pear, and orange) Wang et al. [52] determined TBZ and thiram using a two-dimensional Au@Ag nanodot array as the SERS substrate. The obtained results (very low LOD and limit of quantification (LOQ) values) showed that the novel substrate had high SERS activity for the detection of TBZ and thiram. With their study, the authors showed that the described SERS platform is very easy to use for the determination of multiple pesticides.

Another example of the application of the SERS method with colloidal silver nanoparticles as the SERS substrate was presented by Oliveira et al. [53]. The authors determined TBZ in TBZ solutions and noted enhanced bands of the TBZ SERS spectrum, suggesting that the mechanism of absorption on the silver substrate is through thiazoles. The results showed that reducing the TBZ concentration in the sample causes a reduced SERS intensity. The LOD was lower than the maximum value allowed by the Brazilian regulatory agency ANVISA (10 ppm for citrus fruit).

Hu et al. [54] used gold nanorod arrays as the SERS substrate for the determination of TBZ and thiram on the surface of apples, tomatoes, and pears. The SERS coupled with the self-modelling mixture analysis (SMA) method was used for determination of TBZ and thiram. The SMA method was introduced to separate and identify the spectra of TBZ and thiram, individually from a mixture of spectra. The results obtained showed that it is possible to determine a mixture of pesticides simultaneously and obtain high quality spectra for a single analyte using the SMA method.

Li et al. [55] coupled SERS and variably selected regression methods as one simple and rapid method to determine TBZ in apples. The SERS was enhanced with Au@Ag nanoparticles, which showed a strong sensitivity to TBZ. The authors used three different nonlinear regression models for constructing a quantitative predictive model for TBZ extract, and a competitive adaptive reweighted sampling–extreme learning machine (CARS-ELM) model achieved the best correlation coefficient.

Hussain et al. [56] developed a SERS method for the determination of TBZ and ferbam in liquid milk. The authors synthesized for the first time a substrate based on silver and gold nanoparticles modified with thioglycolic acid (TGA) and characterized it by transmission electron microscope. A newly synthesized substrate showed the possibility of Raman signal amplification for a high enhancement factor. The results showed that the method could detect other classes of pesticides with minimal sample preparation.

In the development of the SERS method for the determination of TBZ, Au@Ag nanoparticles substrates were found to be very good at enhancing the Raman signal, so Chen et al. [57] used the Au@Ag nanoparticles substrate for the determination of TBZ in apples and peaches. The results showed that the Au@Ag nanoparticles had an excellent SERS effect and significantly enhanced the Raman signal. The Raman spectrum of solid TBZ powder is presented in Figure S1 (see Supplementary Materials).

In the last study, Pan et al. [58] combined SERS with chemometrics to establish a detection method for TBZ in citrus. The authors used a variable screening algorithm combined with multiple linear regression. The proposed method was validated, the results were compared with those obtained using HPLC and there was no significant deviation.

The sensitivity and stability as the main disadvantages of this method were eliminated by introducing nanoparticles. The most commonly used nanoparticles were silver and gold as the preferred substrate for pesticide detection. From the results, it can be concluded that SERS could be a suitable alternative method for the detection of TBZ in fruit and fruit juices. In the above studies, rapid and less- or non-destructive methods were used for sample preparation and detection, which is one of the main advantages of the SERS method.

### 8.3. Fluorimetry

Fluorimetry is a quantitative analytical method based on the emission of radiation that occurs when molecules, excited with a photon, relax to the ground state. Compared to absorption methods, it has better sensitivity and accuracy due to the characteristic wavelengths for both excitation and emission. TBZ can be directly determined due to its native fluorescence which is strongly affected by pH. In a weak acidic solution, TBZ has a high intensity of fluorescence while at very low or very high pH values, it has low fluorescence intensity [59]. The determination of TBZ using fluorimetry started in the 1980s. The first fluorometric methods for TBZ determination used ethyl acetate for extraction and were applicable for TBZ determination in fruit and vegetables [60,61]. The following researches were focused on the TBZ determination in water samples [62,63]. The fluorescence emission spectrum of TBZ (excitation wavelength: 299 nm) in a pH 2 buffer aqueous solution is presented in Figure S2 (see Supplementary Materials).

In 2000, Picón Zamora et al. [64] used cross-section fluorimetry combined with various calibration algorithms for TBZ determination in water. The authors studied the influence of the solvent nature and pH on the intensity of the fluorescence signal. They concluded that the maximum fluorescence intensity was obtained at pH values of 2–3 and that by increasing the methanol percentage, fluorescence intensity increased as well. When combined with SPE before the measurements of real samples, this method could be used for the simultaneous determination of TBZ and two other pesticides: carbendazim and fuberidazole. Three years later, the same group of authors led by Rodríguez-Cuesta [65] developed a method for the simultaneous determination of the same pesticides using fluorescence combined with parallel factor analysis (PARAFAC) of the excitation–emission matrix. It was based on the description of every analyte in the sample by one PARAFAC component and the same analyte contribution to the emission for all samples, independently of the other analytes.

In 2001, de Armas et al. [66] combined fluorescence detection with sequential injection analysis (SIA) for the determination of TBZ and fuberidazole, also in water samples. Instead of methanol, they used ethanol as a solvent at pH 2 in order to obtain the maximum fluorescence intensity. In the SIA system, water was used as a carrier. A year later, the same group of authors [67] combined a sandwich SIA with spectrofluorimetric detection and multivariate linear regression algorithms for the simultaneous determination of TBZ and three other pesticides (carbaryl, fuberidazole, and warfarin) in tap and mineral water samples. The application of that method allowed sample segmentation between two buffer zones (two and seven) and recording fluorescence spectra at these pH values in order to improve the method sensitivity and spectral discrimination.

In 2002, Ruedas Rama et al. [68] developed a spectrofluorimetric sensor for TBZ and warfarin determination. The sensor worked on the following principle: a pre-column, made of octadecyl silane C_18_ gel, was used for on-line separation of the analytes, the analytes then arrived to a quartz flow-cell where solid sensing zone (made of the same gel) was placed into detection zone. Warfarin was retained in the pre-column, so TBZ was determined first using methanol 30% (*v*/*v*) as a carrier.

In 2004, García-Reyes et al. [69] developed a flow-through sensor with solid-surface fluorescence detection for TBZ and *o*-phenylphenol detection. C_18_ silica gel, in the form of beads, was placed into the flow cell and TBZ was separated from the other pesticide based on a different retention in the solid zone. That on-line separation/preconcentration/detection strategy was characterized with good selectivity and low reagent consumption but it was not applicable for pesticide determination in drinking water (maximum residue level, MRL = 0.1 μg/L; method quantification limit = 0.3 μg/L). For such samples, SPE should be performed as an enrichment procedure. In the same year, the same group of authors [70] modified their method for the simultaneous determination of TBZ and benomyl in natural water and pesticide formulations. Again, they used C_18_ silica beads placed in the flow cell, as an active solid phase for the separation of analytes. Two years later, the same group of authors [71] developed a flow-through optosensor for TBZ determination in oranges and lemons. Again, they used C_18_ silica gel microbeads as the solid phase in the flow cell. For the sample preparation, the authors used liquid–liquid extraction with acetonitrile and for clean-up, they used dispersive SPE with primary secondary amine (PSA).

In 2007, Piccirilli and Escandar [72] proposed a similar optosensor for TBZ determination. As a solid support, they used nylon powder, so after TBZ immobilization onto a solid support in a continuous flow system the native TBZ fluorescence was measured. There were no extraction or purification steps. In the interference study, the authors found that Cu (II) ions caused a quenching effect due to the formation of complexes with TBZ. In addition, few other pesticides were characterized with emission wavelengths near to those of TBZ, so selectivity of the method should be improved.

Two years later, López Flores et al. [73] developed a flow-through optosensor for determining TBZ and metsulfuron methyl in water samples. As a solid support, the authors used C_18_ silica gel placed in the flow cell. The determination of the TBZ was based on its native fluorescence, while metsulfuron methyl was photochemically converted to a fluorescent photoproduct in micellar medium.

In 2012, Huang et al. [59] proposed a different fluorescence method for TBZ determination in water samples. In order to enhance TBZ fluorescence intensity, the authors studied its complexation with cucurbit [6]uril, cucurbit [7]uril, and tetramethyl-cucurbit [6]uril in aqueous solutions, at pH 6.5. They found that TBZ fluorescence quantum yield increased two or three times due to the formation of host–guest complex which protected the TBZ fluorescent state.

In the same year, Llorent-Martínez et al. [74] developed a method based on SIA with fluorescence detection for TBZ determination in mushrooms. The authors used C_18_ silica gel as a solid support used to retain and preconcentrate TBZ in the detection area in the flow cell. The QuECheRS method was used for the analyte extraction. The liquid–liquid extraction with acetonitrile was followed by dispersive SPE with PSA in order to remove polar matrix components.

Two years later, Zhong et al. [75] determined TBZ in red wine using second-derivative constant-wavelength synchronous fluorescence spectroscopy. Red wine samples were extracted twice using ultrasound-assisted liquid–liquid extraction with dichloromethane. The interference study showed that TBZ could be determined using this method even with higher concentrations of other pesticides present in the sample.

In 2015, Li et al. [76] described DLLME as a sample preparation technique combined with fluorometric detection for TBZ determination in apple juice samples. The analysis of one sample, including sample preparation, lasted only 5 min. After optimization, the chloroform was chosen as the extraction solvent and ethanol as the disperser solvent at pH 8.

Two years later, in order to enhance its fluorescence intensity, Chen et al. [77] developed a fluorometric sensor for Tb^3+^ detection based on a complex between Tb^3+^ as the central cation and TBZ as the ligand. The authors concluded that when Tb^3+^ and TBZ were alone, a weak fluorescence intensity was observed, but when the complex was formed, the strongly enhanced fluorescence was observed. The sensor was also applicable for TBZ determination in juice samples.

In 2018, Murillo Pulgarín et al. [78] presented first-derivative constant wavelength synchronous fluorescence spectrometry as the method for the simultaneous determination of TBZ and 1-naphthylacetic acid in strawberry tree berries and citrus fruit. Although the analytes had overlapped spectra, their simultaneous determination was possible by applying the zero-crossing method to their spectra.

In the same year, Kaur et al. [79] functionalized zinc oxide nanorods with three imidazole-based ionic liquids and then investigated their sensing properties towards TBZ and four other drugs. Only TBZ formed a supramolecular complex with two of the three ionic liquids, thus increasing the fluorescence intensity. Since the fluorescent sensor for TBZ determination had a detection limit in the nanomolar range, the authors concluded that this type of “green” sensor should be further developed and improved.

In the next year, Pagani and Ibañez [80] simultaneously determined TBZ and three other pesticides in lettuce, pear, orange, and mushroom samples using flow injection analysis (FIA) with double pH-gradient and fluorometric detection. TBZ was extracted from the samples using liquid–liquid extraction with ethyl acetate. A four-way calibration model based on excitation–emission matrices as pH function was used for the simultaneous determination of pesticides. The unfolded partial minimum squares (U-PLS) combined with residual trilinealization (RTL) as a multivariate calibration procedure resolved the problem of spectra overlapping.

In 2020, Kazemifard et al. [81] described a new environmentally friendly method to develop a sensor for the determination of TBZ in apple, orange, and tomato juice. The optical sensor they developed consisted of carbon dots synthesized from rosemary as a carbon source, that were modified with MIPs with the TBZ recognition cavities to increase selectivity.

One year later, Peng et al. [82] presented a fluorescent sensor based on Tb^3+^-functionalized MOFs for TBZ determination in oranges. UiO-66-(COOH)_2_ was used as the MOF. The presence of TBZ caused the quenching of the fluorescence intensity of Tb^3+^-functionalized MOF. The samples were prepared using QuEChERS method and the entire process with sample preparation and TBZ detection lasted 35 min.

The most recent research by Chen et al. [83] described chemometrics assisted with excitation–emission fluorescence matrices for determination of TBZ and carbaryl in peach, soil, and sewage. The authors compared three second-order calibration methods and showed that there was no significant difference between the results obtained using these methods. It was confirmed using statistical tests. The proposed method allowed for the accurate determination of analytes despite overlapping peaks from the sample matrix.

Due to its sensitivity, simplicity and low cost, fluorimetry is widely used for TBZ determination in fruit and vegetables, water samples, juices, wines, soil, etc. The samples are usually prepared using liquid–liquid extraction. The extraction can be improved using the SPE or QuEChERS method. However, there are few factors that can affect fluorometric detection such as pH influence and quenching of fluorescence caused by presence of heavy metals. Additionally, overlapping of signals is a big problem for TBZ determination in multicomponent mixtures or during simultaneous determination of TBZ and other pesticides. In order to obtain better results, fluorimetry can be combined with methods such as SIA or FIA. The solid phase fluorimetry can increase the selectivity and sensitivity due to the preconcentration and retention of the TBZ on active solid support, usually C_18_ silica gel. By combining these two approaches, the flow-through optosensors with better properties can be developed. Additionally, in order to improve selectivity, calixarenes and MIPs can be used. Due to the fact that fluorimetry has been used for TBZ determination for more than 30 years, it can be expected that the method will continue to develop and improve in the future.

### 8.4. Room Temperature Phosphorimetry

Phosphorimetry is a quantitative analytical method very similar to the fluorimetry, but unlike the fluorimetry, the energy transitions in phosphorimetry involve a change in electron spin. Consequently, phosphorescence is characterized with slower emission process and has a longer lifetime compared to fluorescence. Due to the complexity and problems related to the analysis at low temperatures, phosphorimetry was less widely used than fluorimetry. In an effort to solve that problem, the room temperature phosphorimetry (RTP) was developed. It is based on the ability of polar or ionic molecules to phosphoresce at room temperature when adsorbed on suitable solid carriers such as silicon, aluminum or cellulose. The advantages of RTP are numerous. High selectivity characterizes this method because a small number of compounds show potential for this type of luminescence. Additionally, compared to absorption methods, the selectivity is higher due to the choice of excitation and emission wavelengths. It leads to the direct analysis of substances which absorb in the same spectral range, but have different spectral emissions. In addition, the method is very precise and sensitive, has a wide linear range and requires a small amount of sample, in the volume range from 3 μL to 5 μL [84].

Segura-Carretero et al. [85] started to use this method for TBZ determination. In 2000, they [86] developed the heavy-atom-induced RTP for TBZ determination without using any organized, protective media. The authors used only high concentrations of potassium iodide as the heavy atom perturber and simple deoxygenation with sodium sulfite. It resulted in interaction between the heavy atom perturber and TBZ, and consequently the production of their triplet states and intense phosphorescence emission. In 2003, the same group of authors [87] developed for the first time a synchronous scanning heavy-atom RTP (SS-HAI-RTP) for the determination of TBZ and carbaryl in water samples. They used potassium iodide as the heavy atom perturber and deoxygenation with sodium sulfite again. The authors demonstrated that it was possible to simultaneously determine various pesticides which overlap in their phosphorence spectrum, such as thiabendazole and carbaryl.

Two years later, Tang et al. [88] established a new method which included a supramolecular complex composed of 1:1:1 β-cyclodextrin (β-CD), TBZ and Triton X-100 with potassium iodide as the heavy atom perturber. This method did not require deoxygenation which made it very simple. Using this method, the detection limit and the concentration of heavy atom perturber were decreased and phosphorescence was significantly prolonged. The authors applied this method to determine TBZ in real samples: tap water, lake water, and fresh pineapple, with excellent recoveries.

Correa and Escandar [89] used a solid nylon carrier which induced the phosphorescence of TBZ. Its intensity was enhanced by the addition of lead (II) acetate as the heavy atom salt and performed the measurements under a nitrogen atmosphere. Both polar and non-polar solvents were used to place TBZ on the surface of the nylon carrier, but water gave the best phosphorescence signal. It resulted in facilitated experimental work, reduced the analysis time and did not use toxic organic solvents. The method had good analytical parameters and was tested on river, mineral, and tap water samples.

Three years later, Piccirilli and Escandar [90] designed a flow-through phosphorescence optosensor for TBZ determination. They also used the nylon as a solid support, but they enhanced the phosphorescence by adding the potassium iodide as a heavy atom perturber and sodium sulfite as oxygen scavenger. They used water as a carrier in a flow-injection system. However, they used methanol for the elution of the system. The analyzed water samples did not contain TBZ, so they were spiked with a standard solution of TBZ at three concentration levels, and the recoveries were satisfactory. The authors also compared the TBZ phosphorescence optosensor to a TBZ fluorescence optosensor, which they characterized earlier, and showed that the phosphorescence optosensor had better selectivity.

Although phosphorimetry is a sensitive analytical method, it was not widely used for TBZ determination. The reason could be use of protective media such as surfactants which causes few problems such as foam formation, and delayed elimination of oxygen dissolved into the micelles. In addition, solutions should be carefully degassed and purified to minimize collisional triplet quenching [91].

### 8.5. Chemiluminescence

Chemiluminescence (CL) reactions include emission of light as the result of a chemical reaction. In general, emission of light is the result of redox reactions in which the oxidant and reductants are a metal complex and a luminophore (analyte or another compound, for example luminol). Transition metals, such as silver (III), copper (III), and nickel (IV) have been applied for the CL reactions as oxidizing agents whereby chelating with polydentate ligands resulted in their stabilization. The development of novel oxidant reagents for the CL reactions became the subject of interest in analytical chemistry [92].

Asghar et al. [93] used CL with flow injection to determine TBZ in tap, rain, and irrigation water samples from various locations. The authors used a diperiodatocuprate (III)–sulfuric acid–CL system. Its CL signal was remarkably enhanced after TBZ addition. An interference study showed that none of the organic compounds, ions or other pesticides interfere with TBZ.

The advantages of the proposed method are simplicity and low LOD. On the other hand, the disadvantage is the sensitivity to various physicochemical factors. That fact can present potential reason for a small number of papers for this method.

### 8.6. Gas Chromatography

Chromatographic methods are used to separate and analyze the components of a mixture. The components carried through the stationary phase, by a mobile phase (gas or liquid), interact differently with the stationary phase based on their chemical and physical properties, such as size, charge, polarity, and affinity. There are few types of chromatography, including gas chromatography (GC), liquid chromatography (LC), etc. Each type of chromatography has its own unique properties and is used for specific applications, but they all rely on the same basic principles of separation and analysis.

GC is a type of chromatography which applies gas as the mobile phase and solid or liquid stationary phase (gas–solid chromatography (GSC), or gas–liquid chromatography (GLC)). This method is used for the separation and analysis of compounds that can be vaporized without decomposition. The main purpose of using the GC method is to control the purity of the substance and separation of some components from a mixture [94]. Determination of TBZ using GC started in the 1970s [95,96]. All of the authors presented in this review used GC coupled to mass spectrometry (MS) for the detection of target compounds, including TBZ.

Maštovská et al. [97] compared a conventional GC method coupled to mass spectrometry (GC-MS) and low-pressure gas chromatography–mass spectrometry (LP-GC-MS) for the determination of 20 pesticides, including TBZ, in carrot extract. The results showed that using the LP-GC-MS method gave better peaks for TBZ, but the influence of the interference was greater and the detection limit increased from 3 ng/g for the GC-MS method to 11 ng/g for the LP-GC-MS method. In the mass spectrum, a peak for TBZ was obtained at *m*/*z* 201 and 174.

In 2007, Walorczyk [98] wrote a paper in which he described using GC-triple quadrupole tandem MS (GC-MS/MS) as a method for pesticide determination in cereals and dry animal feed. TBZ was one of the pesticides that were determined. Re-hydrated samples were extracted with acetonitrile, followed by SPE used as a clean-up step before the chromatographic determination. The advantages of using this method are that the detection and quantification of the pesticides were achieved with a single sample injection which shortened the process and reduced costs. In the mass spectrum, peaks for TBZ were observed at *m*/*z* 201, 174, and 130. One year later, Walorczyk [99] revalidated the previously described multiresidue method for the determination of pesticides in cereals and dry animal feed. The samples were extracted using the buffered QuECheRS method and dispersive SPE with Bondesil PSA and C_18_ sorbents. The optimization of instrument acquisition conditions and application of an efficient clean-up procedure resulted in the improvement of method performance characteristics such as linearity, precision, and accuracy. For the detection, MS was used in electron ionization mode. In the mass spectrum, peaks for TBZ were found at *m*/*z* 201, 174 and 130. In 2011, Walorczyk et al. [100] determined more than 160 pesticides, including TBZ, in white, rosé, and red wines using GC-tandem quadrupole MS (GC-QqQ-MS/MS). Since wine has many different matrix components, various sample preparations were compared. The optimal sample preparation, which gave the best recoveries and lowest RSD values, included an extraction with a citrate buffer and dispersive SPE clean-up with mixed sorbents PSA and C_18_. The MS in electron impact mode resulted in peaks for TBZ at *m*/*z* 201, 174, and 130.

In 2008, Silva et al. [101] used matrix solid-phase dispersion (MSPD) with GC-MS to determine various pesticides, including TBZ, in coconuts. For this method, best parameters, such as the type and amount of solid-phase and eluent, were chosen as follows: 1.0 g of C_18_ was used as the dispersant sorbent, 1.0 g of Florisil was used as the clean-up sorbent and acetonitrile, saturated with *n*-hexane, was used as the eluent. In the mass spectrum, peaks for TBZ were observed at *m*/*z* 201, 174, and 129.

In the same year, Lesueur et al. [102] analyzed 140 pesticides, including TBZ, in various fruit and vegetables. For the determination of pesticides, they used GC and high-performance liquid chromatography (HPLC) coupled with MS (GC/MS and HPLC/MS). Samples of different fruit and vegetables were prepared using QuECheRS method. Authors showed that the QuECheRS method had good accuracy, was applicable to both GC and HPLC, and that it was quick. For the detection of molecular ion, the electrospray ionization (ESI) in positive mode was used as MS ionization technique.

In 2010, Menezes Filho et al. [103] used a GC-MS method to simultaneously determine TBZ and 13 other pesticides in mangoes. For pesticide extraction, direct-immersion solid-phase microextraction (DI-SPME) was used. The developed method proved to be selective, precise, sensitive and applicable for both quantitative and qualitative determinations. For the TBZ detection, the electron impact mode was applied and MS peaks were observed at *m*/*z* 201, 174, and 129.

In 2017, Machado et al. [104] used GC-MS to determine TBZ in globe artichoke leaves and fruit. Modified QuEChERS method was used for sample preparation because it proved to be the best among two others, matrix solid phase dispersion and dispersive ethyl acetate extraction. After validation of the method, real samples were analyzed and all had TBZ concentration below maximum residue level. For MS detection, electron impact mode was used, and MS peaks for TBZ were found at *m*/*z* 202, 175, and 131.

In 2020, Gionfriddo et al. [105] wrote a paper on pesticide analysis in soy milk by using GC-MS for analysis and DI-SPME for sample preparation. Using DI-SPME as the sample preparation method minimized waste and made extraction and desorption process automatic. For the MS detection, electron ionization was used.

The GC method is a highly applicable method for pesticide determination with acceptable values of analytical parameters. In GC analysis, the greatest limitation is the type of compound suitable for the analysis. Compounds appropriate for GC analysis need to have measurable vapor pressure below 350–400 °C, they need to be inert toward mobile or stationary phase, and they need to be stable during vaporization [106]. Some of these limitations could be potential reasons for much lower number of developed methods for TBZ determination using GC, compared to LC.

### 8.7. Liquid Chromatography

Liquid Chromatography is an analytical separation method based on the interaction of the sample components with the stationary and liquid mobile phase. Combined with the MS as a specific detector based on mass analysis, it provides an excellent tool for TBZ determination in complex samples. The European Food Safety Agency (EFSA) recommends LC coupled with tandem MS for TBZ monitoring in the environment (plants, surface water, and groundwater) [107]. Determination of TBZ using LC [108,109] or LC in combination with MS [110,111,112] started in the 1990s. Most of the authors used liquid–liquid extraction, SPE, and QuEChERS methods for sample preparation. For MS detection, most of the authors used the ESI in positive ion mode and ion at *m*/*z* 202 as precursor ion. The product ions of the *m*/*z* 175 (obtained by loss of the cyano group) and *m*/*z* 131 (obtained by further loss of CS) were the most commonly used fragment ions. The MS-MS spectrum of TBZ is presented in Figure S3 (see Supplementary Materials) [113].

In 2000, Fernández-Alba et al. [114] determined TBZ and four other pesticides in pear and tomato samples using reversed-phase (RP) LC for separation, and atmospheric pressure ionization (API) and MS for detection. It minimized the need for complicated sample preparation and purification. The extraction of pesticides was performed using ethyl acetate and sodium sulphate. For TBZ, atmospheric-pressure chemical ionization (APCI) in positive ionization mode and ESI were applied to produce protonated molecular ions [M + H]^+^ and in the mass spectrum, a peak at *m*/*z* 202.1.

One year later, Fernández et al. [115] presented a similar LC-API-MS method for the determination of TBZ and four other post-harvest pesticides in oranges. They used different chromatographic column and mobile phase that resulted with reduced retention time for TBZ. They compared APCI and ESI and obtained similar results so they concluded that both interfaces could be used for determination of studied pesticides. For TBZ determination, they used MS peat at *m*/*z* 202.

In 2001, Pous et al. [116] developed another similar LC-APCI-MS method for the determination of five pesticides, including TBZ, in fruit and vegetables. They improved the extraction method using matrix solid-phase dispersion (MSPD) that reduced consumption of organic solvents, cost, and analysis time. The authors homogenized samples with C_8_-bonded silica as the sorbent, the mixture was placed into a column, and dichloromethane was used as the eluent. They also used APCI in positive mode and peak at *m*/*z* 202.1 for TBZ determination.

In 2004, Yoshioka et al. [117] developed a new LC-MS method for the determination of TBZ and three other post-harvest fungicides and its metabolite in citrus fruit. Instead of ethyl acetate, they used diethyl ether for liquid–liquid extraction. The ionization was performed using atmospheric pressure photoionization (APPI) in both positive and negative ionization modes. That technique also allowed ionization of nonpolar compounds. The mass spectrum for TBZ revealed a peak at *m*/*z* 202, as protonated molecular ions, [M + H]^+^, in positive ion mode, and at *m*/*z* 200, as deprotonated molecular ions, [M − H]^−^, in negative ion mode. Although TBZ was ionized in both ion modes, the negative mode was chosen due to the higher relative abundance of the fragment ions compared to the positive ion mode. The sample preparation time and LC-MS analysis time using this method was approximately two and three hours, respectively, for ten samples. The same group of authors [118] modified their method using time-of-flight (TOF) mass spectrometry which increased sensitivity and improved separation from the matrix background. For TBZ determination, the authors used the fragment ion at *m*/*z* 200.02–200.04 for quantitation and ion at *m*/*z* 173.01–173.03 as qualifier ion.

In the same year, Agüera et al. [119] proposed LC combined with tandem MS (LC-MS/MS) for the determination of 16 pesticides, including TBZ, in vegetable samples. They used a NaOH solution to improve the efficiency of the usual extraction using ethyl acetate and sodium sulphate. The ESI in positive ion mode and collision-induced fragmentation resulted in a final determination of TBZ using peak at *m*/*z* 131. This method was successfully used in a one-year program for monitoring pesticides in 560 samples of vegetables.

Zamora et al. [120] also used LC combined with tandem MS for TBZ and five other pesticides determination in oranges and bananas. They used acetone as an extraction solvent. The ESI in positive ion mode was used to obtain two MS–MS transitions for TBZ determination, so 202.1 → 174.9 and 202.1 → 130.9 were selected as the quantitative and confirmative transitions, respectively. The use of two transitions lowered the possibilities of false results.

In 2005, Dowling et al. [121] developed a LC method with UV detection for the determination of 12 benzimidazole drugs, including TBZ, in bovine liver. The sample preparation was extensive and included extraction with ethyl acetate, defatting with hexane, and purification using SPE.

A year later, Msagati et al. [122] determined TBZ and four other benzimidazole anthelmintics in water and biological matrices (urine and milk), using LC combined with MS. They optimized the sample preparation. The extraction was performed using ethyl acetate. For sample clean-up and enrichment, the authors used and compared the SPE and supported liquid membrane (SLM) technique. As the liquid membrane, they tested five membrane liquids and found 5% tri-*n*-octylphosphine oxide dissolved in *n*-undecane/di-*n*-hexyl ether (1:1) the best. For SPE, the authors compared two types and sorbents and used acetonitrile and aqueous ammonium solution for elution of the analytes. For TBZ detection, ESI was applied to produce protonated molecular ions [M + H]^+^ and in the mass spectrum a peak at *m*/*z* 201.5–202.5.

In 2008, García-Reyes et al. [123] developed a new LC-TOF-MS screening method for the determination of 100 pesticides. The pesticides, including TBZ, were determined in more than 100 fruit-based soft drinks. The screening method was based on a database including retention time and accurate masses of characteristic ions for each pesticide. The authors used SPE for sample treatments. The methanol was used as the eluent. For TBZ detection, protonated molecules, [M + H]^+^, and their fragment ions (*m*/*z* 202.04334) were used. Two years later, the same group of authors [124] improved their method for the determination of 33 pesticides. They used a LC column with smaller particle size which reduced time of the analysis. In addition, the base-line peak width was reduced which improved LOD values. In-source collision-induced dissociation (CID) and isotopic profiles were investigated as identification tools. Two years later, authors [125] used that method for their study on the pesticide occurrence in fruit-based soft drinks. In the same year, the authors [126] proposed a LC method combined with ion trap tandem MS for determination of 10 pesticides, including TBZ, in fruit-based baby food samples. The sample treatment was based on QuEChERS method. For pesticides extraction, they used liquid–liquid extraction with acetonitrile and for clean-up, dispersive SPE with PSA was used. The authors used ESI in positive ion mode to obtain peak at *m*/*z* 202.

In 2009, Moral et al. [127] proposed extraction based on a new supramolecular solvent before detection of three benzimidazole fungicides, including TBZ, in fruit and vegetables using LC with fluorimetric detection. The supramolecular solvent was made from decanoic acid and tetrabutylammonium hydroxide. The extraction was based on hydrophobic and electrostatic interactions, and the formation of hydrogen bonds. It required low volume of sample, was highly efficient, and additional cleaning steps were not needed. In the same year, the authors [128] modified and applied their method for benzimidazole fungicides determination in river and underground water. This time sample preparation started with pH adjustment to 5 using sodium hydroxide and addition of tetrabutylammonium chloride for supramolecular solvent preparation. It caused better recoveries for fungicides through π-cation interactions.

In the same year, Economou et al. [129] used LC-MS/MS for the determination of 46 pesticides, including TBZ, in wines. The sample preparation was based on one-step isolation and clean-up, using SPE. Methanol was used as the eluent. The authors used ESI in positive ion mode to obtain two MS-MS transitions for TBZ determination: 202 → 175 (qualifier ion) and 202 → 131 (quantifier ion). The developed method was successfully used in a one-year program for monitoring pesticides in 60 wine samples.

In 2010, Barahona et al. [130] proposed hollow fiber-liquid-phase microextraction (FH LPME) as the extraction method from orange juices for three post-harvest fungicides, including TBZ. As the organic phase, 2-octanone was used, pH was adjusted to alkaline using sodium hydroxide solution, and HCl was used as the acceptor solution. There was no sample pretreatment before the extraction. However, recoveries obtained were low. Fungicides were determined using LC-MS. The peak at *m*/*z* 202 was used for TBZ detection.

In the same year, Dreassi et al. [131] developed LC method combined with tandem MS for TBZ and four other postharvest fungicides determination in citrus juices. The authors used liquid–liquid extraction with ethyl acetate. The ESI in positive ion mode was used to obtain two MS-MS transitions for TBZ determination: 202.0 → 174.8 (quantification transition) and 202.0 → 130.9 (confirmatory transition).

In 2013, Cho et al. [132] applied a new LC-MS/MS method for the determination of TBZ, carbendazim, and 6-benzyl aminopurine in bean sprout samples. They used solvent extraction with acetonitrile and low temperature partitioning at −80 °C. Using that method, the extracts obtained were transparent and without any interferences. The ESI in positive ion mode was applied for ionization. The product ion of the most intensity was used as the quantifier ion (*m*/*z* 174.8) and the other one at *m*/*z* 130.9 was used as the qualifier.

Two years later, Reichert et al. [133] determined 99 pesticides in 51 fruit jam samples using micro-flow LC combined with tandem MS. Micro-flow LC uses columns with smaller diameter and lower flow rate of the mobile phase, and is characterized by an increased signal-to-noise ratio and improved detection performance [134]. The authors used the QuEChERS procedure for sample preparation. The pesticides were extracted in acetonitrile and the clean-up step followed with magnesium sulfate, PSA, and C_18_ sorbent. Besides the clean-up, the 30-fold dilution step also contributed to reduced matrix interferences and improved selectivity. Again, the ESI in positive ion mode was applied for ionization and TBZ detection was based on two ions (*m*/*z* 174.9 and 131.1). The same group of authors [135] also proposed a LC-MS/MS method for the determination of 16 post-harvest fungicides, including TBZ in oranges and pears. They focused on the sample preparation using a modified QuEChERS procedure. After the optimization, they performed extraction using the acetonitrile. Then, the yttria-stabilized zirconium dioxide was successfully used as the sorbent for dispersive SPE in clean-up step. At the end, the extracts obtained were 10-fold diluted in order to reduce matrix interferences. As usual, peaks at *m*/*z* 175.0 and 131.0 were used for TBZ determination after the ESI in positive ion mode.

In the same year, Dasenaki and Thomaidis [136] determined 115 veterinary drugs, including TBZ, in milk powder, butter, fish tissue, and eggs using LC-MS/MS. The authors optimized the sample preparation. First, they used solid–liquid extraction with a mixture of 0.1% formic acid in 0.1% EDTA solution, acetonitrile, and methanol as the extraction solvent. It was followed by ultrasonic-assisted extraction at 60 °C and a clean-up step at low temperature (−23 °C) in order to amplify the precipitation of fat and proteins. The hexane was used for an additional defatting step. The TBZ was determined after ESI ionization in positive ion mode and again based on two ions (*m*/*z* 175 and 131).

Han et al. [137] described LC with tandem MS as the method for simultaneous determination of TBZ and 69 other pesticide residues in leek, leaf lettuce, and garland chrysanthemum. In order to eliminate pigments and other matrix interferences from the samples, the authors used modified QuEChERS procedure. The extraction was performed using acetonitrile. It was followed by reversed-dispersive SPE method with multi-walled carbon nanotubes (MWCNTs) as clean-up step because in comparison with graphitized carbon black and PSA, better results were obtained using MWCNTs. The TBZ was analyzed in positive ESI mode. The ions at *m*/*z* 202.0 were used as parent ions, while ions at *m*/*z* 175.1 and 131.2 were used as quantifying and qualifying ions, respectively.

In 2016, Boix et al. [138] coupled LC to tandem MS to determine 13 contaminants, including TBZ, in 50 sewage sludge samples, in both liquid and solid phase. For the sample treatment, they used SPE with methanol as the eluent, and lyophilization under vacuum followed by ultrasonic assisted extraction with methanol and water (1:1), for aqueous and solid phases, respectively. Again, after positive ESI mode application, TBZ was determined based on 202.3 → 175.2 and 202.3 → 131.2 transitions used as quantification and confirmation transitions, respectively.

Kim et al. [139] used 126 bean sprout samples to determine TBZ, carbendazim and 6-benzylaminopurine using the QuEChERS method for sample preparation, LC for separation and tandem MS for detection. The extraction was performed using acetonitrile. To reduce the solubility of pesticides in aqueous solution, the authors used sodium chloride and sodium acetate as the salting-out agents. As the clean-up sorbent, magnesium sulfate was used. Peaks at *m*/*z* 174.8 and 130.9 were used for quantification and confirmation of TBZ after positive mode ESI.

Ferreira et al. [140] proposed LC-MS/MS for the determination of 10 pesticides, including TBZ, in coconut water and pulp samples. The modified QuEChERS procedure was applied for sample preparation. The extraction of the pesticides was performed in acidified acetonitrile. Magnesium sulfate and sodium chloride were used as salts for liquid–liquid partition. The PSA and C_18_ sorbent were used for the clean-up step. Again, a positive ESI mode was applied and peaks at *m*/*z* 175.2 and 131.2 were used for quantification and confirmation of TBZ, respectively.

Two years later, Arias et al. [141] developed LC coupled with tandem MS as a method for determination of various veterinary drugs, including TBZ, in milk samples (full cream, semi-skimmed and skimmed milk). The authors modified the QuEChERS procedure using chitosan from shrimp shell waste as a green sorbent in the clean-up step because it can absorb fat and some other biomolecules which can interfere during the analysis. Using that renewable and non-toxic biopolymer also reduced the cost of the method. The acidified acetonitrile was used as a solvent for the extraction of drugs because addition of the acetic acid increased the efficiency of protein precipitation. The ESI in positive ion mode was used to obtain two MS–MS transitions for TBZ determination: 202.18 → 175.2 (quantification transition) and 202.18 → 64.9 (confirmation transition).

Da Costa Morais et al. [142] proposed a LC-MS/MS method for the determination of 13 pesticides, including TBZ, in sweet green peppers. They also used a modified QuEChERS procedure for sample preparation. The acetonitrile was used as the extraction solvent. It was followed by the addition of partitioning salts and citrate buffer, and clean-up using magnesium sulfate and PSA. For TBZ determination, positive ESI mode was applied and peaks at *m*/*z* 174.8 and 130.6 were used.

Cerqueira et al. [143] used hydrophilic interaction LC (HILIC) coupled with tandem MS for the determination of 19 pharmaceuticals, 4 personal care products, and 4 degradation products in sewage sludge collected from wastewater treatment plants. HILIC uses an aqueous-polar/organic solvent as the mobile phase and hydrophilic stationary phase and thus results in better separation and retention. For sample preparation, the authors used MSPD without a solid support. They homogenized samples only by using mortar and pestle. Methanol was used as the extraction solvent. The proposed method resulted in lower reagent consumption and low cost. The ionization was performed using ESI in both positive and negative ion modes.

In 2019, Colazzo et al. [144] proposed a LC-MS/MS method for the determination of 77 semi-polar pesticide residues, including TBZ, in freshwater fish muscle-tissue. As sample preparation methods, acetate buffered and unbuffered QuEChERS methods were compared and the latter was suggested by the authors as a more suitable one due to the better recovery yields. The acetonitrile was used for the extraction of pesticides and the clean-up was performed using magnesium sulfate, PSA, and C_18_ sorbent. As usual, peaks at *m*/*z* 175.1 and 131.1 were used for TBZ determination.

In 2021, Martínez-Piernas et al. [145] also developed a LC-MS/MS method combined with modified QuEChERS procedure for sample preparation. They determined TBZ and 106 other contaminants in wastewater samples. The extraction was performed using acetonitrile and methanol. Due to the matrix complexity, magnesium sulfate and zirconium dioxide-based sorbent were used for the dispersive SPE clean-up. The ESI in positive ion mode was used for ionization. For TBZ determination, the ion at *m*/*z* 202.0 was used as precursor ion, while ions at *m*/*z* 175.1 and 131.1 were product ions.

In the same year, Fares et al. [146] developed LC coupled with tandem MS for the determination of five fungicides, including TBZ, in oranges. For the sample preparation, they used the QuEChERS procedure with acetonitrile as the extraction solvent. They also used positive ESI mode for ionization. Using their method, the authors also studied degradation kinetics of fungicides.

In 2022, Zheng et al. [147] developed a LC-MS/MS method for the determination of TBZ and carbendazim in edible vegetable oil samples. The authors used a simplified and less time-consuming sample preparation method using magnetic flower-like Ni-NiO composite as a sorbent in magnetic SPE. The high affinity of the sorbent towards pesticides was based on the reversible interaction between the Ni (II) ions and the electron-donating imidazole groups in pesticides. That coordination interaction was broke down using acetone with ammonia as the eluent. The ethyl acetate was used to remove interferences. As usual, ESI in positive ion mode was applied, and 202.0 → 175.0 and 202.0 → 131.0 transitions were used as quantification and confirmation transitions, respectively.

As can be seen, LC was widely used for TBZ determination, mainly combined with MS or tandem MS. The reasons are the high specificity and sensitivity of the method. However, LC-MS and LC-MS/MS systems are characterized by a high cost of instrumentation and are not available for many laboratories. In addition, interferences from the matrix can influence the analysis, so clean extracts are crucial. Consequently, sample preparation and additional clean-up steps are usually extensive, complex, and time-consuming. These drawbacks make LC a less desirable method for TBZ determination and encourage the development of other, less complicated methods.

#### 8.7.1. High Performance Liquid Chromatography

The HPLC method was developed in order to eliminate the shortcomings of LC. The main disadvantages of LC are the rate of sample separation and the size of the column packing. These disadvantages were removed through the HPLC method by introducing a high pressure, which resulted in the possibility of separation into smaller and narrower columns, and with that, a faster analysis time was obtained. With smaller and narrower columns comes the possibility of using smaller column fillers, which give a much greater surface area for interactions between the stationary phase and the molecules flowing past it, and that allow for better separation. Over the years, the HPLC method, due to its excellent analytical performance, has become one of most desirable instruments in laboratories.

TBZ determination using HPLC began in the 1980s [148,149], but significant development of the method started in the 1990s [150,151,152,153,154,155]. Due to the modifications and improvements made through the years, the method has become one of the most commonly used methods for benzimidazole determination, including TBZ; coupled with different detection systems, such as UV, fluorescence, and MS. For TBZ determination in pure samples, for determination of impurities, and for determination of TBZ as a plant protection product, the EFSA recommends RP HPLC method with a UV detector (RP-HPLC-UV) [107].

In 2000, De Ruyck et al. [156] proposed two simple, fast, and validated HPLC methods for the determination of five anthelmintic drugs, including TBZ, in milk. They used a diode array as a detector. During sample preparation, acetonitrile and ethyl acetate were added as extraction solvents. Developed methods were suitable for routine analyses of benzimidazole residues in milk.

A few years later, Su et al. [157] developed one more HPLC method that could be appropriate for the routine determination of TBZ and other benzimidazoles in milk and livestock samples. For developing a method, they used six different benzimidazoles extracted with acetonitrile under the basic conditions with a cleaning procedure and two different detectors: a photodiode array (PDA) detector and fluorescence detector. None of the 30 real samples that were tested contained benzimidazole compounds, including TBZ.

In lemon samples, Prousalis et al. [158] determined TBZ, carbendazim, and *o*-phenylphenol by using the RP-HPLC method with UV detection. Acetonitrile, trifluoroacetic acid, and a mixture of ethyl acetate and petroleum ether were used for extraction during the sample preparation and polymeric RP cartridges, with sodium dodecyl sulfate as an ion-pairing agent, were used during the clean-up procedure. The authors concluded that the analytical parameters for this proposed method were satisfactory.

In 2004, Halko et al. [159] proposed, for the first time, the extraction of benzimidazoles (benomyl, carbendazim, TBZ, and fuberidazole) from water samples using a cloud-point extraction (CPE). The extraction was performed with two different non-ionic surfactants, oligoethylene glycol monoalkyl ether (Genapol X-080) and polyoxyethylene 10 lauryl ether (POLE). Using this type of extraction, a better efficiency was achieved compared to other conventional extraction methods, such as SPE. The RP-HPLC was used for the determination of benzimidazoles with a fluorescence detector.

Nozal et al. [160] determined TBZ with seven other azolic fungicides in white, red, and rosé wine samples. The isolation of analytes was performed by SPE on polymeric cartridges and the determination was carried out using HPLC with MS detection in positive ionization mode. The influence of different parameters, such as mobile phase composition, column temperature, corona current, and fragmentor voltage, was studied and then the proposed method was validated. This method is suitable for the simultaneous determination of fungicides in wine samples because it is sensitive, it has a simple sample preparation and it does not require a clean-up step.

In the same year, Turiel et al. [161] determined TBZ in orange juice and fruit samples (grapes, oranges, lemons, and strawberries) using HPLC. The authors first synthesized a MIP using the TBZ molecule as a template and thus obtained a highly selective stationary phase for the HPLC column. Samples were extracted using acetonitrile as a solvent and filtered through 0.45 μm membrane before the injection in the chromatographic system for the separation. In real samples, TBZ was found in strawberry samples and orange and lemon peels.

López Monzón et al. [162] studied the extraction of fungicides in water samples using different types of fiber and optimized SPME as a sample preparation procedure. As a result, SPME with carboxen-polydimethylsiloxane 75 μm fiber, in combination with HPLC with a fluorescence detector, was used for the determination of fungicides in water samples. The authors concluded that this method is suitable for the simultaneous determination of fungicides because it was simple, fast, sensitive, and precise.

In 2008, Hu et al. [163] determined carbendazim and TBZ in apple samples using SPME for sample preparation, HPLC for the separation and fluorescence for detection. SPME was performed at room temperature on a 60 μm polydimethylsiloxane/divinylbenzene (PDMS/DVB) fiber for 35 min prior to the injection into the chromatographic system. Concentrations of TBZ found in real samples were lower than their maximum residue level. This method was selective, sensitive and without interferences.

Wu et al. [164] developed a simple, rapid, and sensitive HPLC method for TBZ and carbendazim determination in soil and water samples. In this work, the authors, for the first time, used DLLME for sample preparation before HPLC analysis. They investigated five different kinds of solvent dispersers (ethanol, methanol, acetone, acetonitrile, and tetrahydrofuran (THF)), and due to the best results, THF was chosen as the disperser solvent. Compared to the other extraction methods such as SPME and SPE, DLLME offers the advantages of simplicity, speed, and low consumption of organic solvents.

In 2011, Barahona et al. [165] performed the HPLC technique with UV and fluorescence detection to determine TBZ in orange juice samples. For SPME, the authors made MIP-fibers which were inside a polypropylene hollow capillary. The extraction consisted of two simultaneous processes: analytes were extracted using LPME from the sample to the organic acceptor solution, and then they were extracted from the organic acceptor solution to a MIP-fiber by SPME. With this step, the authors overcame the problem of selective recognition of MIPs in water samples.

In the same year, using HPLC and fluorescence detection, Asensio-Ramos et al. [166] determined pesticides, including TBZ, and their metabolites in different soil samples (forest, ornamental, garden and lapilli soil). Sample preparation was based on ultrasound-assisted extraction (USE) and DLLME using ionic liquid 1-hexyl-3-methylimidazolium hexafluorophosphate as an extraction solvent and methanol as a dispersion solvent. In this study, ionic liquids were used for the first time as extraction solvents during DLLME procedure in soil samples. This method proved to be rapid, simple, precise, and effective.

Two years later, Han et al. [167] developed a new HPLC method with UV detection for the determination of TBZ, carbendazim, and thiophanate-methyl in tomato samples. In this study, authors combined two types of extraction methods, DLLME and SPE, to provide a pretreatment method for the analysis of pesticides in tomatoes. The authors concluded that the DLLME method had a few advantages, but its main disadvantage was that it could only be applied to simple samples. However, in combination with SPE, this disadvantage could be overcome. The proposed combination of extraction techniques resulted in good analytical performance.

Lin et al. [168] simultaneously determined TBZ, carbendazim, imidacloprid, and acetamiprid in edible fungi using HPLC with UV detection. Before analysis, *Lentinus edodes*, straw mushroom and oyster mushroom samples were extracted with acetonitrile, purified using NH_2_ SPE cartridge and filtered through 0.45 μm filter.

In 2014, Boeris et al. [169] introduced a new HPLC method for the detection of five frequently utilized pesticides (TBZ, fuberidazole, propoxur, carbaryl, and carbendazim) using DAD. The analysis involved the use of multi-variate curve resolution coupled with alternating least-squares (MCR-ALS) to predict the concentration of the five target analytes in a complex matrix sample. Furthermore, the analysis was completed in less than 10 min, which was an improvement on the time required for pesticide analysis.

In 2015, Vichapong et al. [170] put an emphasis on extraction and enhanced it by using a binary mixture of anionic and cationic surfactants as the emulsifier for a method called vortex-assisted surfactant-enhanced emulsification microextraction with solidification of floating organic droplet (VASEME-SFO). The analysis was performed using HPLC with UV detection. Several different animal tissues were used as samples, and results revealed that this improved method could be a great choice for the determination of benzimidazoles in tissue samples. A few years later, the same group of authors [171] improved the extraction using surfactants in the sample preparation. The proposed extraction technique was performed at room temperature with cationic surfactant cetyltrimethyl ammonium bromide in the absence of dispersive solvent. The data obtained demonstrated that this method can be used for the accurate determination of TBZ in milk samples.

In orange samples, Golge et al. [172] determined 115 pesticide residues, including TBZ, using HPLC combined with triple-quadrupole MS for detection. The sample preparation procedure was based on dispersive SPE and QuEChERS methods. In analyzed samples, 112 (including TBZ) out of 115 pesticides were not detected at or above the LOD level.

In 2016, Xu et al. [173] proposed a simple and rapid method for TBZ determination using MWCNTs in the extraction process. A modified QuEChERS method combined with dispersive SPE was used for sample preparation and HPLC with UV detection was used for TBZ detection in fruit juice and wastewater samples.

Amelin and Andoralov [174] identified and determined 111 pesticides, including TBZ, in different samples (milk, meat, fat, eggs, liver, kidney, feed, grain, drinking water, natural water, ground water, and soil). The QuEChERS method was used for simple sample preparation and HPLC with MS detection for the determination of pesticides. For the determination of analytes, the standard addition method was used. The analytes were added in a sample solution in an amount that caused an increase in the peak area by two or three times. The proposed method had several advantages in comparison with the external standard method (calibration curve) such as cost-effectiveness, reduced use of expensive standards and increased accuracy. This study demonstrated that the residues of three to four pesticides can be concurrently present in the analyzed food.

Another simple, convenient, and applicable HPLC method for the simultaneous determination of carbendazim and TBZ in food was proposed by Wang et al. [175]. In this work, the authors used a SPE with carbon nanofibers-packed microcolumn for sample preparation. To achieve better results, several influence factors were studied, such as the amounts of packing material, sample solution acidity, flow rate, and volume. The results have shown that carbon nanofibers had a very good enrichment capability and good stability so the proposed method proved to be a good choice for carbendazim and TBZ determination.

In 2017, Zhang et al. [176] developed and validated a stability-indicated reversed phase ion-paired HPLC method for routine analysis of TBZ in quality control laboratories. In the study, the authors compared several columns for chromatographic analysis. The obtained data indicated that the method was specific, linear, accurate, precise, sensitive, and robust.

Today, it is well known that the use of the SPE material with a good selectivity for imidazole groups plays a key role in TBZ determination in real samples. Therefore, Yu et al. [177] made progress in a sample pretreatment approach using SiO_2_@NiO as a sorbent material in SPE extraction. HPLC with fluorescence detection was used to detect TBZ in fruits and vegetables. In order to improve a developed method, several extraction parameters were optimized (pH, amount of SiO_2_@NiO, and desorption condition).

Another study where MIPs were used as the sorbent material in sample preparation was completed by Garcia-Fernandez et al. [178]. The authors prepared molecularly imprinted core-shell magnetic nanoparticles. The prepared material was characterized and it exhibited a good selectivity for TBZ. HPLC with fluorescence detection was used for TBZ determination. One year later, the same group of authors led by Diaz-Álvarez [179] developed MIPs, which were packed in polypropylene hollow fiber, to provide selectivity to TBZ in sample preparation. The main role of the hollow fiber membrane was to protect the MIP beads from the solid matrix, permitting the extraction and clean-up process without the inclusion of further filtration and/or centrifugation steps. That approach could open up a new path of research in the near future. The newly developed method is suitable for TBZ determination in citrus fruits (oranges and lemons). One year later, the authors published another work [180] and described the same method but with the modification of sample preparation. They developed a molecularly imprinted stir-bar consisting of MIP monolith with magnetic nanoparticles which was used in stir-bar sorptive extraction (SBSE) for the determination of TBZ and carbendazim in orange samples using HPLC. After optimization, the results demonstrated the suitability of this sample preparation method and proposed method for the monitoring of TBZ and carbendazim.

Liang et al. [181] used HPLC with fluorescence detection to determine three benzimidazole residues, including TBZ, in citrus samples. To selectively recognize benzimidazole residues in the samples, an acryloyl-cyclodextrin-based MIP coupled with silanized MWCNT (SMWNTs) was developed by polymerization in a monolithic column with acryloyl-cyclodextrin and methacrylic acid as functional monomers, and SMWNTs as modified materials. Because of the high affinities of the newly developed column, all the benzimidazoles in the fruit samples were successfully extracted simultaneously. Generally, the obtained results confirmed that the combination of HPLC with fluorescence detection and described extraction is an effective, rapid, and simple method for the determination of benzimidazoles in citrus samples.

Based on HPLC with DAD, Lu et al. [182] simultaneously determined TBZ, thiophanate-methyl, and 2-amino-benzimidazole in cherry tomato and red grape samples. In the proposed method, the authors used Tchebichef image moments (TM) models for the quantitative analysis. According to the results, that model could reduce the steps in analyses, and the method could be much simpler and provide more robustness.

Sutcharitchan et al. [183] developed a method for the determination of TBZ and 38 other plant growth regulators in 2 Chinese herbs, using a HPLC with MS detection. The method was validated and applied for analysis of 95 batches of real samples, in which 11 kinds of plant growth regulator residues were detected. The authors proposed this method for routine and quantitative analysis.

In 2020, Zhao et al. [184] used the functionalized material (Ni/triazine-based organic framework-SO_3_H) as an absorbent for the SPE of carbendazim and TBZ in vegetables, fruit, and juices. The mechanism of the new material combined cation–exchange interaction with hydrophilic–lipophilic-balance and at the same time reduced the matrix effect and enriched analytes from the samples. The determination was carried out using the HPLC method with UV detection and according to the results, a new material could be a promising adsorbent for effective and selective SPE for carbendazim and TBZ determination in various samples.

Moreno-González et al. [185] introduced nanoflow LC coupled with Q-Orbitrap-MS detection for the determination of 162 pesticides (from different classes), including TBZ, in specific parts of honeybee samples. The use of this method with quick and simple ultrasound-assisted extraction allowed a reduction in the flow rate, and resulted in improved sensitivity and a strong reduction in matrix effects. The matrix effect was insignificant for 94% of compounds. The suggested methodology can be used to detect a large number of pesticides, with high sensitivity, in specific allocation of bee organs in order to find out how pesticides affect the bees.

In the last study, Choi et al. [186] used HPLC with PDA detection to determine the levels of TBZ in bananas and citrus fruit. The authors validated and optimized the proposed method by comparing the results obtained using various column types, column temperatures, and mobile phase compositions. The results demonstrated that the method was suitable for the quantification and identification of TBZ in solid and liquid foods. After a process of validation, the results were compared with those obtained using HPLC with MS detection. The results obtained using the HPLC-MS method gave lower values and higher LOQ and LOD than the results obtained using the developed HPLC method.

In the studies described above, fruit and vegetables are the most common samples in which TBZ was determined, which is not surprising because of its main application. For extraction and clean-up procedures, various pretreatment methods were used, but the most commonly used were SPE and SPME with acetonitrile and methanol as the extraction solvent. In order to protect the column, samples were filtrated before injection into the chromatographic system. The C_18_ columns with 150 mm × 4.6 mm or 150 mm × 3.9 mm inner diameter with 5 µm size particles were predominant for separations. Acetonitrile and methanol were commonly used as mobile phases. Fluorescence and UV were used for final detection and the MS detector was used in just a few articles.

The HPLC method is a highly applicable method with a very low LOD, high reproducibility, etc. However, the method has some disadvantages. The main disadvantages are the use of a large amount of organic solvents, which is not environmentally friendly, and the equipment for HPLC system is very expensive. Therefore, developing new methods for the determination of TBZ should go toward developing less expensive and more environmentally friendly methods.

#### 8.7.2. Ultra-High-Performance Liquid Chromatography

Ultra-high-performance liquid chromatography (UHPLC) is a type of LC which includes implementation of columns with particles smaller than 2.5–5 µm. Decrease in the column-packing particle size enables an increase in efficiency per unit time, resolution, and sensitivity. Using this method linked with the spectrometric system, such as MS, UHPLC-MS method is developed for drug detection in pharmaceutical as well as the biological matrix [187]. In this review, UHPLC coupled with tandem MS (UHPLC-MS/MS) and UHPLC coupled with high resolution MS (UHPLC-HRMS) were described as methods for the determination of TBZ. Most of the authors used the QuEChERS method for sample preparation. The developed methods combine the selectivity, high resolution capacity, and fast analysis of UPLC-MS/MS with the advantages of QuEChERS, such as being easy to use, the rapidity, and low cost, which have resulted in the generation of simple, rapid and reliable methods for a broad spectrum of analysis. The advantages of described methods are reductions in run time, waste, and use of solvent. Some of the authors used other different methods for sample preparation, such as microwave-assisted extraction (MAE), SPE, and MSPD.

In 2008, Romero-González et al. [188] wrote a paper on using the UPLC-MS/MS method for the simultaneous determination of 90 pesticides, including TBZ in fruit juices (orange, apple, pineapple, peach, and multifruit juices). Samples were prepared using extraction, based on QuEChERS methodology [189] with 1% of acetic acid in acetonitrile. During chromatographic analyses, different mobile phases were chosen with the aim of optimization of the chromatographic method. Mobile phases consisted of methanol, acetonitrile, and water with formic acid or acetic acid at different concentrations. In the case when acetonitrile was used as the mobile phase, a bad peak shape was observed for some pesticides. The addition of formic acid provided better results than acetic acid and was used for the improvement of the ionization efficiency. For the detection of TBZ as an ionization technique, ESI was used and *m*/*z* values in positive mode were 202, 175 and 131. Two years later, the same group of authors [190] used UHPLC-MS/MS to determine several veterinary drugs, including benzimidazoles and avermectins, in egg samples. The authors compared different extraction methods: the QuEChERS method, SPE method, MSPD method, and solvent extraction. The latter proved to be the most efficient and was used in real sample analysis. For MS detection, ESI was also used to produce [M + H]^+^ at *m*/*z* 201.8 while fragmentation ions were found at *m*/*z* 175.2 and 131.2. In their study, one year later, the same group of authors [191] determined TBZ and other veterinary drugs in milk and in powdered milk-based infant formulae using UHPLC with the Orbitrap screening method (UHPLC-Orbitrap-MS). The authors compared this method of screening with quadrupole-time of flight (QqTOF) and triple quadrupole (QqQ) MS, but UHPLC-Orbitrap-MS gave the best quantitative results. For detection, high resolution MS (HRMS) was used and as ionization technique, ESI in positive mode was applied to obtain mass spectrum peaks at *m*/*z* 202.0434, 175.0324, and 131.0604. In 2012, the same research group, led by Perreira Lopes [192], determined the presence of veterinary drugs, including TBZ using UHPLC coupled to QqQ tandem MS as the detection method. Again, they used the QuEChERS method for sample preparation. In this study, chicken meat, specifically muscles, were used as real samples. Mass spectrum peaks were obtained at *m*/*z* 201.8 and 175.2 in positive mode. In the same year, the same research group led by Aguilera-Luiz [193] presented UHPLC-MS/MS as a method for the determination of veterinary drug residues in meat-based baby food and powdered milk-based infant formulae. Samples were prepared by two different methods: the QuEChERS method and “dilute-and shoot” liquid–liquid extraction method. The QuEChERS method gave better results because it allowed a good extraction for simultaneous determination and there was no need for any additional purification. In the mass spectrum, a peak [M + H]^+^ was obtained at *m*/*z* 201.8 and fragmentation ion was obtained at *m*/*z* 175.2. In the same year, the same group of authors [194] again used UHPLC-MS/MS method to determine TBZ and other veterinary drug residues in gilthead sea bream (Sparus aurata). Again, they used the QuEChERS method for sample preparation. Analytes were extracted with a mixture of acetonitrile and methanol (75:25). Before injection into the chromatographic system, samples were filtered through Millex-GN nylon filter. In the mass spectrum, a peak [M + H]^+^ was obtained at *m*/*z* 201.8 while fragmentation ions were obtained at *m*/*z* 175.2 and 131.2. Three years later, the same group of authors, led by Martínez-Domínguez [195], wrote a paper on the determination of pesticides, including TBZ, in nutraceutical products from green tea (Camellia sinensis) by using the QuEChERS method and UHPLC coupled to QqQ tandem MS. Green tea tablets and capsules were homogenized in a coffee grinder prior to extraction. Acetonitrile with 1% acetic acid was used as a solvent for extraction and then magnesium sulfate and sodium acetate (the American version of the QuEChERS method) were added before chromatographic analysis. The method was fast (total run time was 11 min) and reliable. In the mass spectrum, [M + H]^+^ was obtained at *m*/*z* 202, while fragmentation ions were obtained at *m*/*z* 175 and 131.

Additionally, using UHPLC–MS/MS, Whelan et al. [196] determined anthelmintic residues in milk. For sample preparation, the QuEChERS method was also used. Milk samples were extracted in acetonitrile, water solutions of MgSO_4_, and NaCl, vortexed, centrifuged, and filtered before analysis through 0.2 μm polytetrafluoroethylene (PTFE) 13 mm syringe filters. As in the previous mentioned research, to obtain [M + H]^+^, ESI was used as the ionization technique and a peak in the mass spectrum was observed at *m*/*z* 201.9 while fragmentation ions were obtained at *m*/*z* 130.85 and 174.8.

Xia et al. [197] presented a paper on the determination of benzimidazole residue in bovine milk samples. As a method, they used UHPLC-MS/MS, as in the previous mentioned research. Since proteins and fat have to be removed from milk in order to determine trace levels of TBZ and other benzimidazoles, SPE was used for sample preparation. In this study, chromatographic analysis lasted only 8 min. Mass spectrum peaks were observed at *m*/*z* 202, 175, and 131 in positive ion mode.

In 2013, Shang et al. [198] developed an UHPLC-MS/MS method for the determination of four antifungal drugs: enilconazole, parconazole, TBZ, and 5-hydroxythiabendazole in three types of chicken tissue: egg, liver, and muscle. As a sample preparation method, the authors used SPE with the Oasis MCX cartridge. When used in real samples, the method showed that TBZ and its metabolite 5-hydroxythiabendazole were detected in two liver samples. These two compounds were found in a greater concentration in chicken liver tissue compared to chicken muscle tissue. In the mass spectrum, a peak [M + H]^+^ was obtained at *m*/*z* 202 while fragmentation ions were obtained at *m*/*z* 175 and 131.

One year later, Hou et al. [199] used UHPLC-MS/MS for the determination of 38 veterinary drugs, including TBZ, in bovine milk. Milk samples were prepared by adding internal standard and then they were extracted using acetic acid in acetonitrile. Before injecting into the chromatographic system, the samples were filtered through a 0.22 μm filter. The proposed method proved to be specific and good for simultaneous determination with a run time of 13 min and very good analytical parameters. In the mass spectrum, a peak [M + H]^+^ was obtained at *m*/*z* 202.1 while fragmentation ions were obtained at *m*/*z* 131.1 and 175.1.

In 2015, a paper on using UHPLC-TOF-MS to determine 60 pesticides, including TBZ, in various fruit and vegetable samples was written by Sivaperumal et al. [200]. For extraction, the authors used acetonitrile-methanol mixture and sodium chloride. Clean-up step was performed using SPE. In the mass spectrum, [M + H]^+^ was obtained at *m*/*z* 202.0439.

Hanot et al. [201] presented another paper for the determination of pesticides in fruit and vegetable samples in the same year. The authors used solvent extraction with ammonium acetate in methanol/water (95:5) for the sample preparation, UHPLC for separation of analytes, and the MS/MS method for detection. A total of 194 out of 200 pesticides analyzed, including TBZ, had a limit of quantification equal to or below maximum residue levels so this method proved to be good choice in multiresidue determination. In the mass spectrum, [M + H]^+^ was obtained at *m*/*z* 202 while fragmentation ions were obtained at *m*/*z* 175.1 and 131.

A similar method was proposed by Haroune et al. [202] to determine various phytopharmaceutical compounds in insect boluses. For sample preparation, three different approaches were performed: MSPD, the QuEChERS method, and the MAE method, but none of the proposed methods were satisfactory so a two-step procedure called microwave-assisted and salt-out extraction (MASOE) was chosen for sample preparation. By using this precise method, 54 analytes were detected, including TBZ. In the mass spectrum, [M + H]^+^ was found at *m*/*z* 202 while fragmentation ions were found at *m*/*z* 175 and 131.

Additionally in 2015, Chitescu et al. [203] used UHPLC-Q-exactive Orbitrap HRMS as a method for the determination of pharmaceutical and antifungal residues in water samples of the Danube river, collected in various locations in Romanian territory. Before chromato-graphic analysis, SPE was used as the sample preparation method. The described method was selective and sensitive. The results showed that water was contaminated with pharmaceutical and antifungal residues. In the HRMS spectrum, [M + H]^+^ was obtained at *m*/*z* 202.0434.

One year later, Wang et al. [204] used the UHPLC-MS/MS method for the determination of TBZ in bovine milk. Besides TBZ, they also determined various other veterinary drugs and its metabolites. Acetonitrile and acetonitrile-sodium chloride mixture were used for liquid–liquid partition of the samples. Before chromatographic separation, samples were filtered through a 0.2 μm filter. Using this simple, specific, and sensitive method 23 veterinary drugs were determined with satisfactory analytical parameters. In the mass spectrum, [M + H]^+^ was found at *m*/*z* 201.9 and fragmentation ions were found at *m*/*z* 175 and 131.1.

In the same year, Rizzetti et al. [205] wrote a paper on also using the UHPLC-MS/MS method for multiresidue pesticide determination in orange juice samples. For choosing the sample preparation method, interfering components in orange juice were taken into consideration (carbohydrates, proteins, fatty acids, minerals, vitamins, and water). The QuEChERS method was optimized and an optimal extraction method was using acetic acid in acetonitrile and sodium acetate. The detection method, UHPLC-MS/MS, proved to be sensitive and selective at a satisfactory level. In the mass spectrum, [M + H]^+^ was found at *m*/*z* 202. One year later, the same group of authors [206] used the QuEChERS method with acetonitrile and formic acid as the sample preparation method, UHPLC as the separation method, single level standard addition in the sample (SLSAS) as the calibration method, and tandem MS as the detection method. Their aim was to simultaneously determine 72 pesticides, including TBZ, in plant parts of different vegetables and cereals. Compared with other calibration methods, SLSAS gave the best recoveries, reduced the use of solvent and samples, which reduced the total waste and it did not need blank samples for quantification. Molecular ion was detected in positive mode, using ESI as the ionization technique. In the next year, the same group of authors [207] proposed the UHPLC-MS/MS method for the determination of multiresidues of veterinary drugs in bovine tissue samples (liver, kidney, and muscle). During sample preparation, extraction was also performed with acetonitrile, and purification was performed with EMR–Lipid^®^ sorbent and trichloroacetic acid. The authors concluded that this method was effective, simple, sensitive, and with satisfactory results. Detection was performed using ESI as ionization technique in positive mode.

In another type of sample, swine-waste lagoon sludge, Li et al. [208] determined TBZ, along with other 81 veterinary drugs by using the UHPLC-MS/MS method. Before chromatographic separation, samples were prepared by the SPE method using ammonium acetate as a solvent. Total run time of the analysis was 9.5 min. In the mass spectrum, [M + H]^+^ was found at *m*/*z* 202 while fragmentation ions were found at *m*/*z* 175 and 131.

In the same year, Campos-Mañas et al. [209] proposed the UHPLC-MS/MS method for the determination of various pesticides in wastewater. Before chromatographic separation, water samples were filtered through 0.45 μm glass microfiber filter, mixed with acetonitrile and filtered again through a 0.45 μm PTFE filter. Entire analysis lasted less than 15 min and the proposed method was sensitive and suitable for the simultaneous determination of various compounds. Molecular ion was detected in positive mode, using ESI as the ionization technique.

Silva et al. [210] used the UHPLC-MS/MS method for the determination of TBZ in bovine muscle tissue. The authors also determined other benzimidazoles, avermectins, and nitroimidazoles. The samples of bovine muscle tissue were extracted with acetonitrile and acetic acid. Later, samples were vortexed and centrifuged two times (after adding NaCl and Na_2_SO_4_, and after adding dispersive phase and MgSO_4_). Before injection into the chromatographic system, samples were filtered through 22 μm PTFE filter. In the mass spectrum, a precursor ion was obtained at *m*/*z* 202 and product ions were found at *m*/*z* 91.9, 131.0 and 175.0.

In 2018, López et al. [211] wrote a paper on using an UHPLC-HRMS method to determine airborne pesticides in the air. The samples were extracted by microwave-accelerated extraction using ethyl acetate. Before chromatographic separation, samples were dissolved into a water-methanol (70:30) mixture and filtered through a 0.22 μm filter. In presence of matrix, TBZ presented moderate ion suppression. The proposed method used smaller amounts of samples and sorbents, and in general it was useful for the determination of volatile pesticides in air samples. In the HRMS spectrum, molecular ions were obtained at *m*/*z* 202.04334 and 175.09788.

Yao et al. [212] used UHPLC-MS/MS for the determination of TBZ in fish bile samples. In this study, hybrid precipitation on dispersive SPE tubes was used as a purification method to remove proteins and phospholipids from samples. The GC-MS method was used for comparison of the results. The proposed method was sensitive, gave satisfactory results, and it proved to be suitable for the detection of TBZ and personal care products (PCPs). In the mass spectrum, a precursor ion was observed at *m*/*z* 208.1 and product ions were observed at *m*/*z* 136.1 and 180.1.

One year later, Álvarez-Muñoz et al. [213] showed that the QuEChERS method for sample preparation and UHPLC-HRMS for detection represent a good combination for the determination of various contaminants, including TBZ, in four different types of shellfish. The internal standard was added to the samples and the reaction mixture was vortexed and left to equilibrate overnight. The extraction was performed with acetonitrile, water, and a mixture of different magnesium and sodium salts. In the HRMS spectrum, a molecular ion for TBZ detection was observed at *m*/*z* 202.0433.

Liang et al. [214] proposed the UHPLC-MS/MS method for the determination of pharmaceuticals in water samples (Milli-Q water, tap water, lake water and ground water). UHPLC-MS/MS was combined with the cross utilization of two SPE columns and the system was fully automated, which made this method more sensitive when compared with non-automatic extraction, and rapid with only 14 min for analysis. For the SPE method, basic and acidic conditions were used, whereby in the case of TBZ, basic conditions were used for sample preparation. Using this efficient method, authors could simultaneously determine 62 pharmaceuticals, including TBZ, in water samples. Molecular ion was obtained in positive mode, using ESI as the ionization technique.

Qie et al. [215] used the UHPLC-MS/MS method to determine 160 veterinary drugs, including TBZ, in the urine and blood samples of livestock and poultry. The extraction was performed using acetonitrile and EDTA–McIlvaine (citrate-phosphate buffer) as the extraction buffer. In the mass spectrum, a precursor ion was obtained at *m*/*z* 202 and product ions were obtained at *m*/*z* 175 and 131.

Pugajeva et al. [216] used the UHPLC-HRMS method to determine various veterinary drugs in bovine, chicken, and porcine meat samples. Since meat is a complex sample, several different sample preparation methods were compared and the authors concluded that none of the methods was optimal when used alone. They proposed a combination of the solvent extraction method with acetonitrile and formic acid as the solvent, a freezing out step (30 min at −70 °C), and the SPE method using a phospholipid removal column. Molecular ion was obtained in positive mode, using ESI as the ionization technique.

In 2020, Li et al. [217] determined 19 anthelmintics, included TBZ, in river water, tap water, rain water, wastewater treatment plant influent and effluent water, and sediment samples using pressurized liquid extraction (PLE) and SPE as the sample preparation methods, UHPLC as the separation technique coupled with tandem MS as the detection method. The highest concentration of anthelmintics was found in influent water samples and the lowest concentration was found in tap water samples. In the mass spectrum, a precursor ion was obtained at *m*/*z* 202.1, while daughter ions were observed at *m*/*z* 175 and 130.9.

In the same year, Zhan et al. [218] used a combination of extraction with acetonitrile and dimethyl sulfoxide as the sample preparation method, dispersive SPE as a clean-up method, and the UHPLC-MS/MS method for the determination of 291 contaminants in 30 protein powders. In the mass spectrum for the determination of TBZ, a molecular ion was obtained at *m*/*z* 202.1, while product ions were observed at *m*/*z* 175 and 131.

Tomai et al. [219] wrote a paper on also using the UHPLC-MS/MS method for the determination of micro-pollutants, including TBZ. The SPE method for river sediment samples was performed using a methanol–water (50:50) mixture. The purification procedure consisted of stir-disc SPE with buckypaper membrane. Molecular ion was obtained in positive mode, using ESI as the ionization technique.

A year later, Castilla-Fernández et al. [220] proposed the UHPLC-MS/MS method for the determination of pesticides and veterinary drugs in salmon samples. The extraction was performed using acetonitrile with 1% acetic acid. As the clean-up procedure, dispersive SPE was used. The results of the analysis of the salmon samples showed that TBZ was not determined in any sample. Molecular ion was obtained in positive mode, using ESI as ionization technique.

UHPLC is very commonly used method for the determination of TBZ which confirms the high number of papers presented in this review. The method has a low LOD value, high sensitivity, and high reproducibility. Despite these facts, this method has a few disadvantages. In addition to the previously mentioned limitations for the HPLC method such as the use of a large amount of environmentally unfriendly organic solvents, UHPLC uses higher pressure than HPLC for sample separation and it can reduce the lifetime of columns.

### 8.8. Micellar Liquid Chromatography

Micellar liquid chromatography (MLC) is a mode of HPLC in which a pseudo mobile phase consisting of micelles is formed. The analyte separation is based on its distribution between the aqueous mobile phase and the micellar mobile pseudo phase, between the stationary phase and the micellar pseudo phase, and between stationary and aqueous mobile phases [221]. This separation method evolved from the need to separate neutral molecules from the sample mixture. For the determination of TBZ, only two papers by the same authors in 2016 were found for this review.

In the first paper, Peris-Vicente et al. [222] developed a MLC method with UV-Vis detection for the simultaneous monitoring of four post-harvest pesticides (TBZ, pyrimethanil, o-phenylphenol, and imazalil) in wastewater samples. The samples that were taken for analysis were agricultural sewage water, fruit-processing industry wastewater, and influent and effluent water from wastewater treatment plant. There was no complicated sample preparation. Samples were simply filtered. The authors described the advantages of using this method for the determination of TBZ in wastewaters. The complicated and slow sample preparation was avoided, there were no interferences with the wastewater matrix, the method was quick (total time from sample preparation to the end of analysis was 18 min), and inexpensive. Since the authors minimized the use of toxic reagents, reduced the waste in the entire process and also minimized the risk of danger for the operator, this method meets the criteria for green chemistry. In another study, the same group of authors [223] used MLC for the determination of TBZ in wastewaters, but this time using fluorescence detection. The results of the analysis showed that in all samples the concentration of TBZ was above the maximum permitted limits, except in the samples of effluent from the wastewater treatment plant because this water was purified and ready to release into the environment. Compared to the previous study [222], the analysis time was shortened since only two pesticides were analyzed and the fluorescence detector improved the quality. Less compounds from the matrix were detected and the signals from the front of the chromatogram were lower, so the specificity was improved. In addition, the sensitivity was increased due to the lower detection limit. That allows the detection of pollutants in wastewaters at lower levels which implies better monitoring of the environment.

The MLC has acceptable analytical parameters for the determination of TBZ, but is not a commonly used method for its determination, which is confirmed by the very low number of presented papers. One of the main limitations of this method implies a reduced efficiency that is caused by the micelles which can reduce the selectivity of the method and restrict the general application of MLC method. This limitation can present a potential reason for the small quantity of research described in this review for using MLC as the method for the determination of TBZ.

### 8.9. Micellar Electrokinetic Chromatography

Micellar electrokinetic chromatography (MEKC) is a type of capillary electrophoresis used to separate and analyze small molecules in a sample. It is a highly sensitive and efficient analytical technique that provides rapid results with minimal sample preparation. In MEKC, a sample is injected into a capillary tube filled with a buffer solution containing a surfactant. An electric field is then applied to the capillary, causing the migration of the analytes in the sample through the buffer solution at different rates depending on their size, shape, and charge. The surfactant molecules form micelles around the analytes, which can alter their mobility and affect their separation. As the analytes move through the capillary, they are detected by a detector, such as a UV detector or the MS, at the end of the tube. The MEKC offers several advantages over other separation techniques, including high separation efficiency, minimal sample preparation, and low sample consumption. However, MEKC is not a common method for the determination of TBZ and only two studies investigated its determination using that technique.

In 2001, Rodríguez et al. [224] developed a MEKC method for the simultaneous determination of eight pesticides, including TBZ, in grapes, lettuces, tomatoes and oranges. The SPE was used for sample preparation. The authors optimized the parameters of the method such as pH, buffer concentration, type and concentration of surfactant, and methanol content in the mobile phase to obtain the best results. The results showed that the combination of SPE and MEKS is suitable for the simultaneous determination of studied pesticides.

After a long period, in 2014 Bol’shakov et al. [225] developed one more MEKC method for the determination of 27 polar pesticides, including TBZ, in soil using QuEChERS sample preparation. The authors demonstrated that this method provides good separation and resolution of pesticides and can be used for the quantification of multiple pesticides in soil samples with high accuracy and precision. They suggest that this method could be useful for the routine monitoring of pesticide residues in soil and environmental monitoring.

In MEKC, separation is based mainly on charge and hydrophobicity, and compounds with similar properties may be difficult to separate, causing a very low sensitivity. On the other hand, potential interference could come from the micelles, which are used as the separation medium in MEKC and can sometimes interfere with the separation of some compounds, leading to poor separation and detection. Due to these shortcomings, MEKC is not popular for the determination of TBZ.

### 8.10. Capillary Electrochromatography

Capillary electrochromatography (CEC) is a hybrid separation technique that combines the advantages of both capillary electrophoresis and HPLC. Compared to LC, capillary electrochromatography (CEC) achieves much higher component separation efficiency. When the samples are neutral molecules, separation is performed exclusively according to the interaction of the sample components with the stationary phase, while for samples with electrically charged particles, the separation is performed both according to the interaction with the stationary phase and electrophoretic mobility of the component. Separation selectivity can be controlled by a good choice of stationary phase, and choice of good material and capillary dimensions [226]. Despite the good properties of the method, CEC is not commonly used for the determination of TBZ. The possible reason could be that TBZ has a relatively low molecular weight and may not be well suited for separation using CEC.

Cacho et al. [227] used CEC to determine TBZ in citrus fruit samples. The authors constructed a molecularly imprinted capillary column filled with a molecularly imprinted monolith which was used as the stationary phase. Acetonitrile was added to the samples and the mixture was sonicated. Then, the supernatant was filtered, extracts were evaporated and dissolved again in acetonitrile for the analysis. All analysis was performed under the 2 kV voltage and at 60 °C. Different mobile phases (methanol, acetone, chloroform and acetonitrile) were used in parallel experiments to determine the optimal one, which resulted in being acetonitrile. The accuracy of the method was confirmed by comparison of the results with the results obtained using HPLC.

### 8.11. Capillary Electrophoresis

Capillary electrophoresis (CE) is an analytical technique which implies the separation of ions based on their electrophoretic mobility with the use of an applied voltage. Factors affecting electrophoretic mobility are: the charge of the molecule, viscosity, and radius of the atoms [228]. This method is performed in a capillary tube. Beside the capillary tube, other parts of CE systems are high-voltage power supply, a sample introduction system, a detector, and an output device. In addition, some instruments can include a temperature control device, due to the fact that the viscosity of the solutions, as one of the factors affecting electrophoretic mobility, decreases as the column temperature rises [229].

CS with ESI-MS detection (CE-ESI-MS) was used in 2002 by Rodríguez et al. [230] for the purpose of the determination of TBZ and procymidone in fruit and vegetable samples (apples, grapes, oranges, pears, strawberries, and tomatoes). Samples were prepared by SPE and then analyzed. This method was selective and sensitive which makes it a good choice for determination at levels lower than established maximum residue levels.

Ten years later, Hu et al. [231] described capillary zone electrophoresis (CZE) as a method for the determination of TBZ in animal tissue (swine muscle and liver). The entire process included magnetic SPE (MSPE) of the sample with magnetite/silica/poly magnetic microspheres (methacrylic acid-co-ethylene glycol dimethacrylate, Fe_3_O_4_/SiO_2_/poly), field-amplified sample stacking (FASS), and CZE for the determination of TBZ. The results of the analysis of the real samples revealed that the real samples did not contain TBZ so for recovery research the samples of swine muscle and liver had to be spiked.

Xu et al. [232] used non-aqueous CE (NACE) for detection of TBZ, imazalil, and prochloraz in apples, cherry tomatoes, and grape juice. All samples were prepared by DLLME using tetrahydrofuran and chloroform as disperser and extraction solvent, respectively. The authors showed that this method could be applicable for the determination of pesticides in fruit samples because of its short separation time (<5 min), sensitivity, and repeatability.

In 2017, Oliveira et al. [233] presented a CE method with spectrophotometric detection at 210 nm as a method for the determination of TBZ in tap and river water samples. For the sample preparation, electromembrane extraction was used. For this procedure of extraction, which was proved to enhance the sensitivity of CE, polypropylene hollow fiber, impregnated with 1-ethyl-2-nitrobenzene, was used as a liquid membrane.

In the same year, Tejada-Casado et al. [234] used CZE-MS/MS to determine TBZ in poultry and porcine muscle. As a clean-up procedure, dispersive liquid–liquid microextraction with acetonitrile was performed. The authors wanted to present the method for the simultaneous determination of 12 different benzimidazoles in meat samples, and they succeeded because this method was precise, efficient, and selective.

The CE has various separation modes which enables its application to a wide range of samples. One of the simplest and most commonly used CE modes is CZE. This mode can be used for the analysis of a wide range of the compounds including inorganic ions, amino acids, proteins, and peptides. The very important advantage of this method is its efficiency. Small capillary diameters enable very efficient heat dissipation and the application of high voltages [235]. On the other side, CE cannot be “scaled up” to preparative CE, sin the way that, for example, HPLC can be, which presents one of the disadvantages of this method. In addition, it is sensitive to impurities in the sample or buffer, can be complex and expensive, and can be less effective for the separation of high molecular weight compounds, compared to other separation methods. These drawbacks probably limit its wider use for the determination of pesticides, including TBZ.

### 8.12. Voltammetry

Voltammetry is an electroanalytical technique used to study the behavior of electroactive species, such as ions or molecules, in a solution. It is a powerful analytical technique that involves the measurement of current as a function of applied potential, providing information on the redox behavior of electroactive species in a solution. There are several types of voltammetry, including cyclic voltammetry (CV), differential pulse voltammetry (DPV), square wave voltammetry (SWV), and others. Each type of voltammetry has its own advantages and disadvantages and is suitable for different types of electrochemical systems. Voltammetry is widely used in analytical chemistry, electrochemistry, and materials science, among other fields.

Msagati and Ngila [236] described SWV and DPV methods for the voltammetric determination of five benzimidazoles, including TBZ, using a glassy carbon rotating-disk electrode modified with poly(3-methylthiophene). The authors investigated the effect of pH and scan rate on the electrochemical behavior of the drugs and found that the drugs could be selectively determined at pH 2.0. Different voltammetric measurements were performed, and the modification of the carbon rotating-disk electrode with poly(3-methylthiophene) enhanced the sensitivity of the method. All the data were compared, and the results obtained using SWV demonstrated higher sensitivity, lower LOD, and better resolving power compared to those obtained using DPV.

Yang et al. [237] developed a voltammetric method for the sensitive detection of TBZ in real samples and investigated its interaction with human serum albumin, which could potentially elucidate its toxicity and pharmacokinetics. They used a glassy carbon electrode modified with MWCNTs and gold nanoparticles to enhance the electrochemical response of TBZ. The interaction between TBZ and human serum albumin was investigated using CV and fluorescence spectroscopy. The results showed that TBZ interacts with human serum albumin through hydrogen bonding and hydrophobic interactions.

Dong et al. [238] developed glassy carbon electrode modified with ZnFe_2_O_4_/SWCNTs as a novel nano-hybrid material based on single-walled carbon nanotubes (SWCNTs), for the simultaneous voltammetric detection of the benzimidazole fungicides carbendazim and TBZ in apples, leeks, and tomatoes. The method was also successfully applied to the analysis of real water samples with good recoveries, indicating its potential as a sensitive and reliable tool for the detection of benzimidazole fungicides in environmental monitoring.

In 2020, Ribeiro et al. [239] developed a novel electrochemical sensing method for the detection of TBZ in complex samples using a cathodic-pretreated boron-doped diamond (BDD) electrode and SWV. The BDD electrode exhibited excellent electrochemical performance and stability for the detection of TBZ. The developed method was successfully applied to detect TBZ in mango, sugar cane, river waters, and pharmaceutical formulations. The results demonstrated that the BDD electrode-based electrochemical sensing method is a promising technique for the sensitive and selective detection of TBZ in complex samples.

Voltammetry was generally used for the determination of TBZ in fruits. In all studies, Ag/AgCl was used as a reference electrode and platinum wire as an auxiliary electrode. The working electrode was a glassy carbon electrode modified with a different substance. Sample preparation was very simple: fruits and vegetables were crushed, filtrated, and dissolved in ethanol.

Voltammetry has advantages such as high sensitivity, selectivity, rapid response, and low sample volume. However, its limited range of applications and possible interferences could be disadvantages. In the case of the determination of TBZ, there are additional challenges. TBZ has a high tendency to adsorb onto the surface of the working electrode, which can lead to a decrease in the sensitivity of the voltammetric methods. Another problem can be that TBZ is a relatively large and complex molecule, which can make it difficult to obtain well-defined and reproducible voltammetric responses. These disadvantages make voltammetry a less commonly used method for the determination of TBZ.

### 8.13. Potentiometry

Potentiometry is an electrochemical method used to determine the concentration of ions in a solution based on measuring the potential difference between two electrodes (indicator and reference) immersed in the analyte solution. The measured potential difference is related to the concentration of ions in the solution. The ion-selective electrodes (ISEs), as indicator electrodes, respond to changes in the ion concentration of ions in the sample solution. Usually, ISEs have a liquid membrane containing ionophore as sensing material, plasticizer, and polyvinyl chloride. The optimization of the membrane components, as well as modification using nanomaterials leads to the development of potentiometric methods with improved analytical parameters. There are only a few methods for the determination of TBZ using potentiometry although it is a very simple method and does not require complicated sample preparation.

In 2016, Volnyanska et al. [240,241] started their study on the determination of TBZ using direct potentiometry. They determined TBZ in banana peel and pulp samples. The authors prepared (TBZH_2_)_3_(PMo_12_O_40_)_2_ ionic associate and used it as sensing material for ISE with a plasticized membrane. The Ag/AgCl electrode was used as the reference. After optimization, tricresyl phosphate was chosen as the plasticizer for ISE and pH 4 as the optimal pH value for measurements. The response time of the electrode was pretty long (2–3 min at low concentrations and 40–50 s at higher concentrations). The sample preparation was simple. Bananas were chopped, placed in a glass of water, and left for 12 h. After filtration through the cheesecloth and acidification to pH 4, measurements were performed. The proposed ISE represents a simple sensor for the determination of TBZ, but its main drawback is a slow response time.

In 2022, Budetić et al. [242] proposed a new potentiometric sensor for the determination of TBZ in fruit peels (oranges, lemons, and bananas). The authors developed ISE with an internal filling solution (3M NaCl) and an ionic pair of TBZ cation and the 5-sulfosalicylate anion, as the sensor material. They optimized the membrane of the sensor by varying the content of the sensor material and using six different plasticizers. The response time of the new sensor was only 8 s. For the determination of TBZ, the Gran method and direct potentiometry were compared. Due to the better results, TBZ was determined in fruit peels using the Gran method. The accuracy and influence of the matrix components were checked using the known addition method. For simple preparation of fruit samples, fruit were chopped and covered with water, pH was adjusted to 2.6, and after 24 h, the samples were filtered. In the same year, the same group of authors led by Dandić [243] continued to develop TBZ sensors. Instead of conventional ISE with a liquid electrolyte, they used a solid-state sensor. This time, MWCNTs modified with a sulfate group and TBZ cation were used as the sensing material. That modification caused improved analytical parameters. The applicability of the sensor was demonstrated in fruit samples (four citrus fruit and bananas) where TBZ was determined using the Gran method.

Due to its simplicity, selectivity, and low-cost, potentiometry could be a good alternative for the fast and accurate determination of TBZ in real samples. However, it is important to consider the fact that some substances present in the sample can interfere with measurement and cause inaccurate results. Additionally, the simultaneous determination of TBZ and other pesticides using potentiometry could be challenging and time-consuming. Some of these drawbacks could explain the low number of papers describing the potentiometric determination of TBZ.

### 8.14. Immunoassay

Immunoassay is a bioanalytical method based on the selective recognition of antigen and antibody. The most commonly used type of immunoassay is enzyme-linked immunosorbent assay (ELISA) which uses enzymes for the labeling of antigens or antibodies. In the 1990s, Brandon [244,245,246] and his team and Bushway and his team [151,153,247] started to develop this method for the determination of TBZ in fruit, vegetables, juices, and bovine liver. They used horseradish peroxidase (HRP), monoclonal antibodies which bound TBZ or its metabolites, and determination spectrophotometrically at 414 or 450 nm.

The competitive strip-based immunoassay is a type of immunoassay proposed by Blažková et al. [248] to determine TBZ in samples of various fruit juices. The nitrocellulose membrane strip was coated with a conjugate of TBZ and protein in the test zone. Carbon particles were used as a label, so during the test, the sample and the conjugate of carbon particles and antibodies migrated along the membrane strip by capillary forces. The strip-based immunoassay was based on the following principle: when the strip was placed in the reaction mixture, carbon-labelled secondary antibodies bound to the anti-TBZ antibodies thus forming a detection complex. If TBZ was not present in the sample, a black product of the strongest intensity appeared, because the detection complex bound to the conjugate of TBZ and protein. On the other hand, if TBZ was present in the sample, the anti-TBZ antibodies were neutralized in the detection complex and the intensity of the black product was decreased in proportion to the concentration of TBZ. The intensity of the black color on the strip allowed a semiquantitative visual assessment of the TBZ concentration while quantitative evaluation was performed using scanning densitometry.

In 2012, Estevez et al. [4] developed an indirect competitive immunoassay for the determination of TBZ in whole oranges using surface plasmon resonance (SPR). The conjugate of TBZ hapten and bovine serum albumin (BSA) was used as an antigen which was covalently immobilized on the gold surface of the sensor, while the antibodies were injected into the device. The higher sensor response was observed due to the lower analyte concentration and, consequently, the higher amount of the free antibodies available to interact with the antigen surface. The authors concluded that this type of immunoassay was sensitive, fast, and reusable because the bioactive surface could be regenerated for more than 100 cycles.

Three years later, Tsialla et al. [7] developed a competitive indirect enzyme immunoassay to determine TBZ in white and red wines. The assay was based on a conjugate of TBZ and BSA, as a solid-phase reagent, and monoclonal anti-TBZ antibodies. The determination of TBZ was based on measuring the optical density at 405 nm after the addition of chromogenic peroxidase substrate. This method proved to be fast, precise, and in good correlation with the LC-MS/MS analysis.

One of the most important characteristics of immunoassays is simple sample preparation. In most cases, in papers previously described, it has included homogenization, extraction with dimethyl sulfide, methylene chloride or methanol, and centrifugation. In the research described, immunoassay was successfully applied to the determination of TBZ in various types of samples due to its high selectivity and specificity. Additionally, the method is applicable for the analysis of a large number of samples because it is fast. However, immunoassay is characterized with possible problems such as the instability of reagents and high costs, which is the reason for the less common use of this method, compared to other methods.

**Figure 5:**
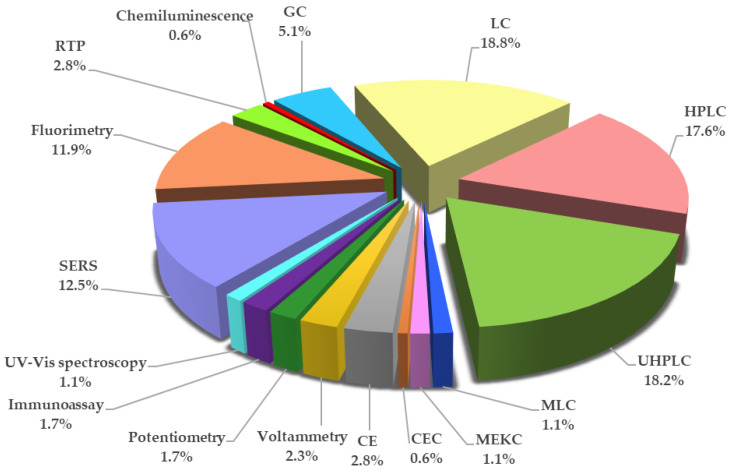
Distribution of analytical methods, described in this review, for the determination of TBZ in the period from 2000 to 2023.

In the period from 2000 to 2023, many analytical methods for TBZ determination have been developed. Table 2, Table 3 and Table 4 present the summarized characteristics of spectrometric methods, chromatographic methods, and other methods, respectively, for the determination of TBZ described above. Chromatographic methods were the most frequently used (63% of the described methods) which was to be expected due to their good analytical performances. SERS (represented by 13% of the described methods) was also a commonly used method for the determination of TBZ despite its low sensitivity compared to those of chromatographic methods. The potential reasons could be simple and fast sample preparation, good accuracy, and rapid and non-destructive analysis. The wide use of fluorimetry (12% of the described methods) is not surprising, due to the native fluorescence of TBZ. A graphical presentation of the percentage of methods used for the determination of TBZ can be seen in Figure 5.

## 9. Conclusions

TBZ is a widely used fungicide and anthelmintic drug that is commonly found in food products, especially fruit and vegetables. The detection of TBZ in food is important due to its potential toxicity and potential impact on human health. This review gives important information about the synthesis and structural characteristics of TBZ which are responsible for its biological activity. Generally, it summarizes all the important information about the properties of TBZ and the advantages and disadvantages of methods for its determination and thus presents a starting point for all research in this area.

There are a lot of methods available to determine the presence and concentration of TBZ in various samples (fruit, vegetables, water, animal products, etc.), including chromatography-based methods, immunoassays, electrochemical and spectroscopy-based methods. The most commonly used methods for the determination of TBZ are LC methods, including the more sensitive HPLC and UHPLC. However, despite their superior analytical characteristics such as high sensitivity, selectivity, accuracy, and the ability to analyze a wide range of sample matrices, LC methods have some serious drawbacks. These drawbacks include the high consumption of organic solvents, complicated and time-consuming sample preparation, and expensive instrumentation. Spectroscopy-based methods are also frequently used for the detection of TBZ. However, they may suffer from low sensitivity and specificity compared to chromatographic methods. Surprisingly, electrochemical methods are rarely used for the determination of TBZ, regardless of their simplicity, availability in most laboratories, and low cost. In addition, compared to chromatographic methods, electrochemical methods do not require the consumption of toxic, organic solvents, which is crucial because environmental safety is one of the most important concerns today. Considering the above, electrochemical methods have high potential for further development.

In conclusion, the choice of method for TBZ detection depends on several factors, including the analytical requirements, sample matrix, cost, and accessibility of the method. Therefore, the optimal method should be selected based on the specific requirements of the analytical task. Despite the large number of developed methods for the determination of TBZ, there is still potential for further improvements and optimizations in order to develop the most convenient method for the determination of TBZ, in a specific medium and sample.

## Figures and Tables

**Figure 4:**
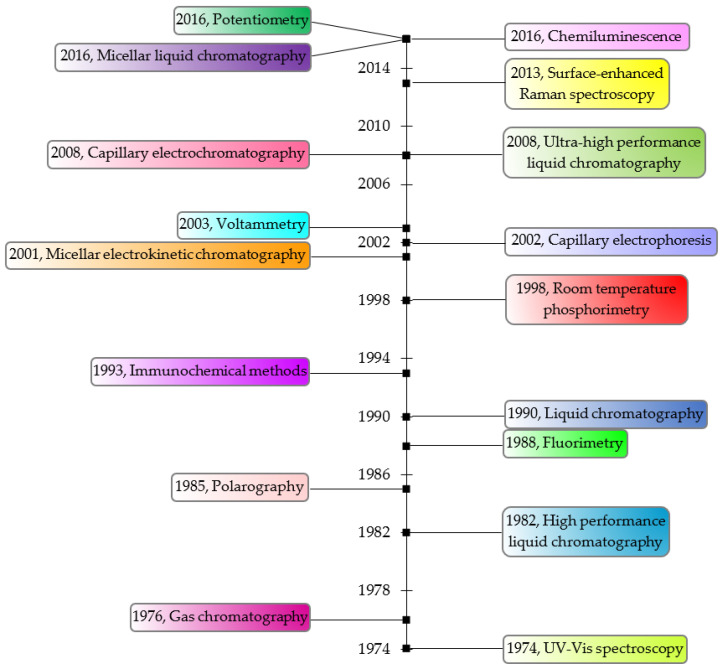
Chronological overview of the beginnings of development of methods for TBZ determination.

**Table 1:** Physicochemical properties.

Molecular weight (g/mol)		201.25
LogP		2.47
Hydrogen Bond Donor Count		1
Hydrogen Bond Acceptor Count		3
Exact mass (Da)		201.03606841
pKa (at 25 °C)		4.64
Solubility (g/L at 25 °C)	*n*-heptane	0.1
	methanol	8.28
	acetone	2.43
	ethyl acetate	1.49

**Table 2:** Spectrometric methods for the determination of TBZ.

Method	Sample	Conditions and Characteristic Method Parameters	Measuring Range (μg/mL)	Correlation Coefficient	LOD (μg/mL)	RSD (%)	Recovery (%)	Reference
UV-Vis spectroscopy	fruit, vegetables	max. absorption: 340 nm; solvent: 1-butyl-3-methylimidazolium hexafluorophosphate	3.0 × 10^−4^–2.8 × 10^−1^	0.9948	1.0 × 10^−4^	2.1–5.6	95.1–98.2	[37]
UV-Vis spectroscopy	fruit	max. absorption: 305 nm; eutectic solvents: betaine with 2-furoic acid, phenylacetic acid, mandelic acid, and glycolic acid	4.0 × 10^−4^–1.5 × 10^−1^	0.9996	1.0 × 10^−4^	1.2–3.8	91.0–106.0	[38]
SERS	lemon	solvent: water and ethanol; EF: Ag colloids (500 µL); spectral resolution: 10 cm^−1^; WN: 200–3400 cm^−1^; Ex: 532 nm; laser power: 40 mW	-	-	4.0 × 10^−6^	-	-	[39]
SERS	fruit	solvent: water and ethanol; EF: Ag colloids (500 µL); spectral resolution: 8 cm^−1^; room temperature; WN: 200–3400 cm^−1^; Ex: 532 nm; laser power: 40 mW	-	-	4.0 × 10^−6^	-	-	[6]
SERS	apple	solvent: methanol; EF: Ag dendrites (5 µL); spectral resolution: 3 cm^−1^; room temperature; WN: 500–2000 cm^−1^	-	0.9770	0.1	-	89.2–115.4	[40]
SERS	apple	solvent: 1% acetic acid in acetonitrile; EF: Au nanoparticles (50 µL); WN: 550–2000 cm^−1^; laser power: 80 mW	-	0.9630–0.9770	2.0 × 10^−2^	3.6–6.7	>90	[41]
SERS	TBZ solution, orange	solvent: water and ethanol; EF: Au nanoparticles (100 µL); WN: 500–1700 cm^−1^; Ex: 785 nm; laser power: 15 mW	1.0 × 10^−3^–100 (TBZ solution) 0–2.5 ^1^ (orange)	0.9764 (TBZ solution) 0.9926 (orange)	1.0 × 10^−2^ (TBZ solution) 1.3 × 10^−2 1^ (orange)	1.4–12.2 (TBZ solution) 2.2–19.4 (orange)	-	[42]
SERS	orange	solvent: acetonitrile; EF: Ag colloids (0.24 µL); WN: 400–2000 cm^−1^; Ex: 785 nm; laser power: 25 mW	-	0.9900	4.0	-	87.3–108.8	[43]
SERS	rapeseed	solvent: acetonitrile; EF: Ag nanoparticles (500 µL); spectral resolution: 2 cm^−1^; WN: 200–3300 cm^−1^; Ex: 785 nm; source power: 200 mW	-	0.7400–0.9100	0.1	0.7–9.9	94.7–118.9	[44]
SERS	orange	solvent: ethanol; EF: Au coated paper; spectral resolution: 0.6 cm^−1^; WN: 400–1800 cm^−1^; Ex: 632 nm	-	-	2.0 × 10^−2^	10.0–20.0	-	[45]
SERS	red soil	solvent: acetonitrile; EF: Au nanoparticles; spectral resolution: 2 cm^−1^; WN: 200–3300 cm^−1^; Ex: 785 nm; source power: 200 mW	-	0.9892–0.9948	0.1	1.4–10.5	71.8–116.7	[46]
SERS	lemon, carrot, and mango juices	solvent: acetone–water (50:50); EF: gold nanorods (10 µL); spectral resolution: 6 cm^−1^; WN: 500–1800 cm^−1^; Ex: 785 nm; laser power: 20 mW	-	0.9800–0.9900	1.5 × 10^−1^–1.8 × 10^−1^	-	96.0–99.0	[3]
SERS	cherry	solvent: methanol–dichloromethane (1:1); EF: Ag sol–polyurethane (80 µL); Ex: 785 nm; laser power: 3 mW	-	-	2.0 × 10^−2^	-	82.0–94.0	[47]
SERS	TBZ in methanol solution, apple	solvent: acetonitrile–water (50:50); EF: Au nanorods; WN: 600–1700 cm^−1^; Ex: 785 nm; laser power: 50 mW	-	0.9910 (methanol solution) 0.9800 (apple)	3.7 × 10^−2^ (methanol solution) 6.0 × 10^−2^ (apple)	15.4	76.0–107.0 (apple)	[48]
SERS	peach	EF: Au-IP6-Mil-101(Fe); WN: 600–1700 cm^−1^; Ex: 633 nm	1.5–7.5	5.0 × 10^−2^	0.9860	2.7–17.2	84.3–113.1	[49]
SERS	TBZ solution, apple surface, apple juice	solvent: water; EF: Ag@SiO_2_ nanocubes; WN: 600–1200 cm^−1^; Ex: 633 nm; laser power: 90 mW	1.0 × 10^−3^–1.0	0.9920	1.9 × 10^−2^	8.8	-	[50]
SERS	mango	solvent: water; EF: Au nanoparticles; spectral resolution: 2 cm^−1^; WN: 200–3200 cm^−1^; Ex: 785 nm; source power: 250 mW	3.0 × 10^−2^–10.0	-	-	2.3	-	[51]
SERS	pear, apple, orange	solvent: methanol; EF: Au@Ag nanoparticles; WN: 600–1800 cm^−1^; Ex: 633 nm; laser power: 4.25 mW	-	0.9620–0.9800	5.1 × 10^−2^	10.5	76.0–134.0	[52]
SERS	TBZ solution	solvent: methanol; EF: Ag nanoparticles (980 µL); WN: 200–1800 cm^−1^; Ex: 514.5 nm;	1.6 × 10^−2^–3.2 × 10^−2^	-	1.4 × 10^−2^	-	-	[53]
SERS	apple, tomato, pear	solvent: methanol; EF: Au nanorods; WN: 543–1683 cm^−1^; Ex: 633 nm; laser power: 17 mW	1.0 × 10^−2^–100.0	0.9777–0.9803	1.5 × 10^−2^–1.6 × 10^−2^	-	74.3–127.7	[54]
SERS	apple	solvent: ethanol; EF: Au@Ag nanoparticles; WN: 600–1800 cm^−1^; Ex: 785 nm	1.0 × 10^−3^–1.0 × 10^3^	0.9406	1.0 × 10^−3^	<10	83.0–93.5	[55]
SERS	milk	solvent: water; EF: Au@Ag nanoparticles–TGA; WN: 350–1800 cm^−1^; Ex: 633 nm; laser power: 50 mW	-	0.9880	1.2 × 10^−1^	4.1–9.2	88.0–103.0	[56]
SERS	apple, peach	solvent: acetonitrile and methanol; EF: Au@Ag nanoparticles; WN: 500–1800 cm^−1^; Ex: 785 nm; source power: 140 mW	0.1–100.0	0.9770–0.9980	3.2 × 10^−2^–3.4 × 10^−2^	3.9–4.4	95.0–101.0	[57]
SERS	citrus fruit	solvent: methanol; EF: Au nanorods (5.0 µL); WN: 200–3200 cm^−1^; Ex: 785 nm	-	0.9737	0.33	<5	-	[58]
fluorimetry	water	solvent: methanol; pH = 2–3; Ex: 300 nm, Em: 340 nm	5.0 × 10^−3^–4.0 × 10^−2^	>0.9900	2.9 × 10^−4^	2.1–5.4	97.0–102.0	[64]
fluorimetry	pesticides	solvent: methanol; Ex: 260–306 nm, Em: 310–370 nm	-	0.9990	4.7 × 10^−3^	2.0	-	[65]
fluorimetry combined with SIA	water	solvent: ethanol; pH = 2; FR: 6.6 mL/min; carrier: water; Ex: 301 nm, Em: 355 nm	8.0 × 10^−5^–2.0 × 10^−2^	-	2.0 × 10^−5^	0.5	96.0–106.0	[66]
fluorimetry combined with SIA	water	solvent: ethanol; pH = 2; FR: 6.7 mL/min; Ex: 301 nm, Em: 355 nm	1.9 × 10^−4^–6.0 × 10^−2^	0.9997	0.3 × 10^−4^	0.7	90.0–104.0	[67]
fluorimetry combined with FIA	water, pharmaceutical preparations, commercial pesticides	solvent: methanol–water (50:50); pH = 2; FR: 1.14 mL/min; Ex: 309 nm, Em: 354 nm	1.0 × 10^−2^–0.8	0.9997	2.4 × 10^−3^	0.9	97.1–104.5	[68]
fluorimetry combined with FIA	water	solvent: ethanol; FR: 1.16 mL/min; carrier: methanol–water (20:80); Ex: 305 nm, Em: 358 nm	5.0 × 10^−4^–1.6 × 10^−2^	0.9995	9.0 × 10^−5^	0.7–2.0	97.0–103.0	[69]
fluorimetry combined with FIA	water, commercial pesticides	solvent: ethanol; FR: 1.16 mL/min; carrier: methanol–-water (20:80); Ex: 305 nm, Em: 358 nm	4.0 × 10^−4^–2.0 × 10^−2^	0.9996	8.0 × 10^−5^	0.2–0.8	98.8–102.0	[70]
fluorimetry combined with FIA	citrus fruit	solvent: acetonitrile; FR: 1 mL/min; carrier: methanol–water (25:75); Ex: 305 nm, Em: 358 nm	0.3–10.0 ^1^	0.9995	9.0 × 10^−2 1^	1.5–4.0	87.1–108.9	[71]
fluorimetry combined with FIA	water	solvent: methanol; FR: 0.9 mL/min; carrier: water; Ex: 300 nm, Em: 340 nm	8.0 × 10^−3^–1.2 × 10^−1^	-	2.8 × 10^−3^	0.2–1	98.0–105.8	[72]
fluorimetry combined with FIA	water	solvent: methanol, 0.04 M SDS-0.01 M NaOH; pH = 12; FR: 2.4 mL/min; carrier: methanol–water (15:85); Ex: 296 nm, Em: 347 nm	8.0 × 10^−3^–0.2	0.9950	2.5 × 10^−3^	1.1	93.2–108.3	[73]
fluorimetry	water	solvent: water; pH = 6.5; Ex: 302 nm, Em: 355 nm, 358 nm	1.2 × 10^−2^–1.6	0.9998	1.1 × 10^−3^	0.5–2.2	91.9–103.6	[59]
fluorimetry combined with SIA	mushroom	solvent: acetonitrile; FR: 10 mL/min; carrier: methanol–water (30:70); Ex: 305 nm, Em: 345 nm	1.6–40.0 ^1^	0.9992	0.5 ^1^	2.0–5.0	93.0–110.0	[74]
fluorimetry	red wine	solvent: methanol; Ex: 315 nm, Em: 355–365 nm	5.0 × 10^−2^–1.0	0.9980	7.2 × 10^−3^	1.8–7.8	85.9–102.8	[75]
fluorimetry	apple juice	solvent: methanol; pH = 8; Ex: 302 nm, Em: 345 nm	5.0 × 10^−3^–5.0 × 10^−2^	0.9987	2.2 × 10^−3^	2.1–7.9	94.1–108.1	[76]
fluorimetry	orange juice	solvent: acetonitrile; pH = 10; Ex: 320 nm, Em: 546 nm	1.6–8.1	0.9990	-	1.6–2.5	101.1–104.6	[77]
fluorimetry	strawberry tree berries, citrus fruit	solvent: ethanol; pH = 4.7; Ex: 304 nm, Em: 350 nm	3.0 × 10^−2^–1.2 × 10^−1^	0.9920	1.7 × 10^–4^	0.4–2.3	99.9–102.8	[78]
fluorimetry	TBZ solution	solvent: ionic liquids; Ex: 352 nm, 317 nm, Em: 350 nm, 357 nm	2.0–16.1	-	2.4 × 10^–3^	-	-	[79]
fluorimetry combined with FIA	fruit, vegetables	solvent: methanol; FR: 0.15 mL/min; carrier: acid sample (in acetic acid); Ex: 210–310 nm, Em: 320–410 nm	9.0 × 10^−3^–4.0 × 10^−2^	-	3.0 × 10^−3^	-	82.0–115.0	[80]
fluorimetry	apple, orange, tomato juice	solvent: universal buffer solution (boric, acetic, and phosphoric acid, all 0.04 M and NaOH solution); pH = 7; Ex: 300 nm, Em: 360 nm	3.0 × 10^−2^–1.7	-	8.0 × 10^−3^	2.6–5.5	92.5–105.1	[81]
fluorimetry	orange	solvent: acetonitrile; Ex: 300 nm, Em: 544 nm	5.5 × 10^−2^–16.1	0.9950	5.5 × 10^−2^	1.6–2.7	98.4–104.5	[82]
fluorimetry	peach, soil, sewage	solvent: methanol–water (40:60); Ex: 302 nm, Em: 420 nm	-	0.9930–0.9950	1.0 × 10^−4^ (soil) 1.1 × 10^−3^ (sewage) 1.2 × 10^−3^ (peach)	0.3–2.5	86.2–109.2	[83]
RTP	aqueous solution	Ex: 300 nm, Em: 488 nm; in solution; heavy atom salt: KI; deoxygenation: Na_2_SO_3_; lifetime: 89 µs	1.5 × 10^−2^–1.5 × 10^−1^	-	1.5 × 10^−2^	4.5	-	[86]
RTP	water	Ex: 306 nm, Em: 450 nm; in solution; heavy atom salt: KI; deoxygenation: Na_2_SO_3_; lifetime: 100 µs	0.0–1.8 × 10^−1^	-	1.4 × 10^−3^	1.1–3.0	78.8–114.3	[87]
RTP	water, pineapple	Ex: 298 nm, Em: 481 nm; in solution; β-CD, TBZ and Triton X-100 (1:1:1); heavy atom salt: KI; lifetime: 800 µs	2.0 × 10^−2^–8.2 × 10^−1^	0.9937	2.1 × 10^−3^	1.9	95.0–102.0	[88]
RTP	water	Ex: 300 nm, Em: 485 nm; in solid phase; nylon; heavy atom salt: Pb (II) acetate; deoxygenation: N_2_	3.2 × 10^−2^–2.6 × 10^−1^	-	1.0 × 10^−2^	2.4	93.0–118.0	[89]
RTP	water	Ex: 300 nm, Em: 485 nm; in solid phase; nylon; heavy atom salt: KI; deoxygenation: Na_2_SO_3_	1.3 × 10^−2^–1.1 × 10^−1^	0.9985	4.5 × 10^−3^	3.2	97.0–110.0	[90]
CL	water	solvent: ethanol; glass spiral flow cell (1.5 mm I.D.); FR: 2.8 mL/min; reaction time: 8 s	1.0 × 10^−3^–2.0	0.9999	3.0 × 10^–4^	1.1–2.9	92.0–108.0	[93]

EF = enhancement factor; WN = wavenumber range; Ex = excitation; Em = emission; FR = flow rate; SDS = sodium dodecyl sulfate ^1^ Measuring unit is µg/g.

**Table 3:** Chromatographic methods for the determination of TBZ.

Method	Sample	Conditions and Characteristic Method Parameters	Measuring Range (μg/mL)	Correlation Coefficient	LOD (μg/mL)	RSD (%)	Recovery (%)	Reference
LP-GC-MS	carrot extract	solvent: toluene; MP: helium; column: Gas-Chrom G(HP) (Sigma-Aldrich, Darmstadt, Germany) (3 m × 0.15 mm); t_R_: <20 min; 250 °C, detection: *m*/*z* 201, 174; selected ion monitoring mode	-	-	3.0 × 10^−3 1^	-	-	[97]
GC-MS/MS	cereals, dry animal feed	solvent: acetonitrile; MP: helium; column: Gas-Chrom G(HP) (Sigma-Aldrich, Darmstadt, Germany) (30 m × 0.25 mm) with 0.5 μm particles; t_R_: 16.46 min; temperature: 300 °C detection: *m*/*z* 201, 174, 130; electron ionization mode	-	-	4.0 × 10^−3 1^	7.0–10.0	103.0–124.0	[98]
GC-MS/MS	cereals, dry animal feed	solvent: 5% formic acid in acetonitrile; MP: helium, FR: 1.2 mL/min; column: J&W Scientific DB-5 MS (Agilent, Santa Clara, CA, USA) (30 m × 0.25 mm) with 0.5 µm particles; t_R_: 16.31 min; detection: *m/z* 201, 174, 130; electron ionization mode	1.0 × 10^−2^–0.5	0.9999	-	3.0–9.0	92.0–112.0	[99]
GC-MS/MS	wine	solvent: acetonitrile; MP: helium, FR: 1.2 mL/min; column: DB-5 (Agilent, Santa Clara, CA, USA) (30 m × 0.25 mm) with 0.5 µm particles; t_R_: 16.4 min; temperature: 300 °C; detection: *m/z* 201, 174, 130; electron impact mode	1.0 × 10^−2^–0.2	0.9989	-	4.0–17.0	89.0–95.0	[100]
GC-MS	coconut pulp	solvent: acetonitrile, *n*-hexane; MP: helium, FR: 1.8 mL/min; column: J&W Scientific DB-5MS (Agilent, Santa Clara, CA, USA) (30 m × 0.25 mm) with 0.25 µm particles; t_R_: 19.26 min; detection: *m*/*z* 201, 174, 129; electron impact ionization	9.0 × 10^−2^–10.0	0.9979	8.0 × 10^−2 1^	4.5–14.7	74.1–88.4	[101]
GC-MS	fruit, vegetables	solvent: acetone–ethyl acetate (1:1); MP: helium; column: Agilent Technologies HP 5 MS (Agilent, Santa Clara, CA, USA) (30 m × 0.25 mm) with 0.25 µm particles; temperature: 280 °C; ESI; positive mode	5.0 × 10^−2^–10.0	-	-	7.3–12.9	102.0–119.0	[102]
GC-MS	mango	solvent: methanol; MP: helium, FR: 1.0 mL/min; column: Restek Rtx^®^-1 MS Crossbond (Restek, Rheinfelden Switzerland) (30 m × 0.25 mm) with 0.25 µm particles; temperature: 250 °C; detection: *m*/*z* 201, 174, 129; electron impact mode	3.3 × 10^−2^–1.67	0.9948	1.0 × 10^−2 1^	5.8–15.6	78.5–95.1	[103]
GC-MS	artichoke leaves, fruit	solvent: ethyl acetate containing triphenyl phosphate; MP: helium, FR: 1.0 mL/min; column: TR-5MS Thermo (Termo Fisher Scientific, Neuilly-sur-Seine, France) (30 m × 0.25 mm) with 0.25 µm particles; t_R_: 9.1 min; detection: *m/z* 202, 175, 131; electron impact mode	-	-	-	6.7–17.5	70.8–71.3	[104]
GC-MS	soy milk	solvent: acetone–water (3:7); MP: helium, FR: 1.5 mL/min; column: Agilent Technologies HP- 5MS (Agilent, Santa Clara, CA, USA) (30 m × 0.25 mm) with 0.25 µm particles; 260 °C, electron ionization	5.0 × 10^−3^–1 ^1^	0.9972	1.0 × 10^−3 1^	4.0–16.0	81.0–121.0	[105]
LC-MS	pear, tomato	solvent: acetonitrile–water (50:50); MP: acetonitrile and ammonium formate in water–acetonitrile (95:5) (gradient), FR: 1 mL/min; column: Zorbax SB-C_8_ (Agilent, Santa Clara, CA, USA) (150 × 4.6 mm) with 3.5 μm particles; t_R_: 9 min; vaporization temperature: 400 °C; detection: *m*/*z* 202.1; EA; APCI; positive ion mode	5.0 × 10^−2^–5.0 ^1^	0.9990	5.0 × 10^−4^	-	95.0–108.0	[114]
LC-MS	orange	solvent: acetonitrile–2 mM ammonium formate (50:50); MP: methanol and 2 mM ammonium formate in water (gradient), FR: 1 mL/min; column: Zorbax SB-C_18_ (Agilent, Santa Clara, CA, USA) (30 × 4.6 mm) with 3.5 μm particles; t_R_: <4 min; vaporization temperature: 325 °C; detection: *m*/*z* 202; EA; APCI; positive ion mode; 350 °C	5.0 × 10^−2^–5.0 ^1^	-	2.0 × 10^−2 1^	7.0	75.0	[115]
LC-MS	fruit, vegetables	solvent: methanol; MP: methanol and 50 mM ammonium formate (gradient), FR: 1 mL/min; column: Phenomenex Luna C_18_ (Phenomenex, Torrance, CA, USA) (150 × 4.6 mm) with a Phenomenax C_18_ guard cartridge (4 × 2 mm), both with 5 μm particles; t_R_: <8 min; vaporization temperature: 450 °C; detection: *m*/*z* 202.1; APCI; positive ion mode	5.0 × 10^−2^–10.0 ^1^	>0.9940	1.0 × 10^−2 1^	10.0–15.0	63.0	[116]
LC-MS	citrus fruit	solvent: methanol; MP: methanol and water (gradient), FR: 0.5 mL/min; column: Inertsil ODS-3 (GL Sciences, Tokyo, Japan) (150 × 3 mm) with 5 μm particles; t_R_: <4 min; vaporization temperature: 450 °C; detection: *m*/*z* 200; APPI; negative ion mode	-	-	1.0 × 10^−2 1^	2.0–12.0	82.0–100.0	[117]
LC-MS	citrus fruit	solvent: methanol; MP: methanol and water (gradient), FR: 0.5 mL/min; column: Inertsil ODS-3 (GL Sciences, Tokyo, Japan) (150 × 3 mm) with 5 μm particles; t_R_: 3.0–4.2 min; vaporization temperature: 350 °C; detection: *m*/*z* 200.02–200.04, 173.01–173.03; APPI; negative ion mode	-	-	2.0 × 10^−3 1^	2.2–5.4	82.8–91.7	[118]
LC-MS/MS	vegetables	solvent: methanol; MP: ammonium formate and acetonitrile–methanol (3:1) (gradient); FR: 0.2 mL/min; column: Polaris C_18_-A (Polaris, Medina, WA, USA) (150 × 2 mm) with 3 μm particles and with a Varian precolumn (30 × 2 mm); t_R_: 6.53 min; desolvation temperature: 300 °C; detection: *m*/*z* 131; ESI; positive ion mode	1.0 × 10^−2^–1	>0.9900	1.5 × 10^−3 1^	5.0–28.0	83.0–95.0	[119]
LC-MS/MS	banana, orange	solvent: acetone, diluted with methanol (1:5); MP: 2.5 mM ammonium acetate-0.01% formic acid in methanol (gradient); FR: 0.3 mL/min; column: Nucleosil C_18_ (Macherey-Nagel, Düren, Germany) (125 × 2.1 mm) with 5 μm particles; t_R_: 12.80 min; desolvation temperature: 350 °C; detection: *m*/*z* 174.9, 130.9; ESI; positive ion mode	1.1 × 10^−3^–0.1	>0.9990	5.0 × 10^−5^ (bananas) 0.01 ^1^ (oranges)	3.0–8.0	83.0–88.0	[120]
LC	bovine liver	solvent: methanol–water (50:50); MP: ammonium dihydrogen phosphate buffer pH 6.8-methanol-acetonitrile (gradient); FR: 0.5 mL/min; column: Waters stainless-steel C_18_ (Waters, Wien, Austria) (150 × 3.0 mm) with a Waters guard column (3 × 2 mm), both with 3.5 μm particles; t_R_: <13 min; temperature: 40 °C; UV detection (298 nm)	2.5 × 10^−1^–15.0	≥0.9900	1.2 × 10^−1 1^	4.6–24.0	56.0–96.0	[121]
LC-MS	water, urine, milk	solvent: methanol–water (1:1) containing 5% formic acid; MP: acetonitrile and acetic acid (gradient), FR: 0.15 mL/min; column: Waters XTerra^®^ C_8_ (Waters, Wien, Austria) (50 × 2.1 mm) with 3.5 μm particles; t_R_: <8 min; detection: *m*/*z* 201.5–202.5; ES	1.0 × 10^−5^–1.0	0.9900	1.0 × 10^−7^	-	74.0–100.0	[122]
LC-MS	fruit-based soft drinks	solvent: methanol–water (1:2); MP: water with 0.1% formic acid and acetonitrile (gradient), FR: 0.6 mL/min; column: Zorbax Eclipse XDB-C_8_ (Agilent, Santa Clara, CA, USA) (150 × 4.6 mm) with 5 μm particles; t_R_: 9.8 min; temperature: 325 °C; detection: *m*/*z* 202.04334; ESI; positive ion mode	1.0 × 10^−4^–5.0 × 10^−2^	0.9996	9.0 × 10^−6^	6.9–10.1	95.3–104.6	[123]
LC-MS	fruit-based soft drinks	solvent: methanol–water (1:2); MP: water with 0.1% formic acid and acetonitrile (gradient), FR: 0.5 mL/min; column: Zorbax Rapid Resolution Eclipse XDB-C_18_ (Agilent, Santa Clara, CA, USA) (50 × 4.6 mm) with 1.8 μm particles; t_R_: 4.37 min; temperature: 325 °C; detection: *m*/*z* 202.0427; ESI; positive ion mode	1.0 × 10^−4^–5.0 × 10^−2^	0.9987	-	3.4–8.0	88.6–104.7	[124]
LC-MS	fruit-based soft drinks	solvent: methanol–water (1:2); MP: water with 0.1% formic acid and acetonitrile (gradient), FR: 0.5 mL/min; column: Zorbax Rapid Resolution Eclipse XDB-C_18_ (Agilent, Santa Clara, CA, USA) (50 × 4.6 mm) with 1.8 μm particles; t_R_: 1.5 min; temperature: 325 °C; detection: *m*/*z* 202.0433; ESI; positive ion mode	-	-	1.0 × 10^−5^	6.5	-	[125]
LC-MS/MS	baby food	solvent: 10% methanol; MP: water with 0.1% formic acid and acetonitrile (gradient), FR: 0.6 mL/min; column: Zorbax Eclipse XDB-C_8_ (Agilent, Santa Clara, CA, USA) (150 × 4.6 mm) with 5 μm particles; t_R_: 7.6 min; temperature: 350 °C; detection: *m*/*z* 202; ESI; positive ion mode	-	0.9978	3.0 × 10^−3 1^	-	-	[126]
LC	fruit, vegetables	supramolecular solvent: decanoic acid, tetrabutylammonium hydroxide; MP: phosphate buffer pH 4–methanol (60:40); FR: 1 mL/min; column: Kromasil C_18_ (Eka Chemicals, Bohus, Sweden) (150 × 4.6 mm) with 5 μm particles; t_R_: <11 min; fluorimetric detection; Ex: 300 nm, Em: 350 nm	5.0 × 10^−5^–6.0 × 10^−2^	0.9999	4.0 × 10^−4 1^	2.2–3.5	95.0–100.0	[127]
LC	water	supramolecular solvent: decanoic acid, tetrabutylammonium chloride, tetrabutylammonium hydroxide; MP: phosphate buffer pH 4-methanol (60:40); FR: 1 mL/min; column: Kromasil C_18_ (Eka Chemicals, Bohus, Sweden) (150 × 4.6 mm) with 5 μm particles; t_R_: <11 min; fluorimetric detection; Ex: 300 nm, Em: 350 nm	1.0 × 10^−5^–1.6 × 10^−2^	0.9999	4.0 × 10^−6^	4.0	95.0–102.0	[128]
LC-MS/MS	wine	solvent: methanol; MP: 0.1% formic acid and 0.1% formic acid in acetonitrile (gradient); FR: 0.2 mL/min; column: XTerra C_18_ (Waters, Wien, Austria) (150 × 2.1 mm) with a XTerra C_18_ guard column (10 × 2.1 mm), both with 3.5 μm particles; t_R_: 2.7 min; temperature: 350 °C; detection: *m*/*z* 175, 131; ESI; positive ion mode	1.0 × 10^−4^–0.1	0.9970 (white wine) 0.9950 (red wine)	4.5 × 10^−4^ (white wine) 8.8 × 10^−4^ (red wine)	6.7–9.2	87.0–88.0	[129]
LC-MS	orange juice	solvent: ethanol; MP: 20 mM formic acid and methanol (gradient), FR: 0.05 mL/min; column: BioBasic 8 C_8_ (Bio Basic, Markham, IL, USA) (50 × 1 mm) with 5 μm particles; t_R_: <9 min; detection: *m*/*z* 202; ESI; positive ion mode	1.0 × 10^−4^–1.0 × 10^−2^	0.9990	5.0 × 10^−5^	7.9–10.6	-	[130]
LC-MS/MS	citrus juice	solvent: 0.05% formic acid; MP: 0.05% formic acid and acetonitrile (gradient), FR: 0.2 mL/min; column: Varian Pursuit C_18_ (Varian, Santa Clara, CA, USA) (50 × 2 mm) with 3 μm particles; t_R_: 4.3 min; desolvation temperature: 300 °C; detection: *m*/*z* 174.8, 130.9; ESI; positive ion mode	5.0 × 10^−5^–1.5 × 10^−2 1^	0.9970	1.7 × 10^−5 1^	4.0–8.9	95.3–98.6	[131]
LC-MS/MS	bean sprouts	solvent: acetonitrile; MP: 10 mM ammonium formate and methanol (gradient), FR: 0.5 mL/min; column: YMC Pack Pro C_8_ (YMC, Kyoto, Japan) (150 × 4.6 mm) with 3 μm particles; t_R_: 10.02 min; temperature: 350 °C; detection: *m*/*z* 175, 131; ESI; positive ion mode	1.0 × 10^−2^–1.0 ^1^	0.9980	2.0 × 10^−3 1^	0.5–1.8	88.9–96.3	[132]
LC-MS/MS	fruit jam	solvent: acetonitrile; MP: 0.1% formic acid and acetonitrile with 0.1% formic acid (gradient), FR: 0.03 mL/min; column: Eksigent Halo C_18_ (Eksigent Technologies, Framingham, MA, USA) (50 × 0.5 mm) with 2.7 μm particles; t_R_: 0.46 min; temperature: 400 °C; detection: *m*/*z* 174.9, 131.1; ESI; positive ion mode	9.0 × 10^−3^–0.6 ^1^	0.9800	9.0 × 10^−3^	2.0–6.0	65.0–107.0	[133]
LC-MS/MS	orange, pear	solvent: acetonitrile; MP: 0.1% formic acid and acetonitrile with 0.1% formic acid and 5% of water (gradient), FR: 0.3 mL/min; column: Zorbax Eclipse plus C_8_ (Agilent, Santa Clara, CA, USA) (100 × 2.1 mm) with 1.8 μm particles; t_R_: 1.31 min; temperature: 375 °C; detection: *m*/*z* 175.0, 131.0; ESI; positive ion mode	1.0 × 10^−3^–0.5 ^1^	0.9989 (orange) 0.9996 (pear)	-	0.0–1.0	77.0–96.0	[135]
LC-MS/MS	milk powder, butter, fish tissue, egg	solvent: methanol–0.05% formic acid (25:75); MP: 0.01% formic acid and methanol (gradient), FR: 0.1 mL/min; column: Atlantis T3 C_18_ (Waters, Wien, Austria) (100 × 2.1 mm) with 3 μm particles and guard column; t_R_: 9.12 min; temperature: 300 °C; detection: *m*/*z* 175, 131; ESI; positive ion mode	1.0 × 10^−3^–0.2	>0.9970	-	4.0–6.8	56.1–101.0	[136]
LC-MS/MS	leek, leaf lettuce and garland chrysanthemum	solvent: acetonitrile; MP: acetonitrile and 0.1% formic acid (gradient), FR: 0.3 mL/min; column: Hypersil GOLD C_18_ (Termo Fisher Scientific, Waltham, MA, USA) (100 × 2.1 mm) with 3 μm particles; t_R_: 1.11 min; temperature: 300 °C; detection: *m*/*z* 175.1, 131.2; ESI; positive ion mode	1.0 × 10^−2^–1.0	≥0.9992	3.0 × 10^−4 1^	2.1–14.7	81.0–109.0	[137]
LC-MS/MS	sewage sludge	solvent: methanol–water (1:9); MP: water and methanol, both with 0.1 mM ammonium acetate and 0.01% formic acid (gradient), FR: 0.3 mL/min; column: Waters Symmetry C_18_ (Waters, Wien, Austria) (50 × 2.1 mm) with 3.5 μm particles; t_R_: 4.8 min; temperature: 350 °C; detection: *m*/*z* 175.2, 131.2; ESI; positive ion mode	5.0 × 10^−4^–0.5	0.9990	-	1.0–42.0	92.0–122.0	[138]
LC-MS/MS	bean sprouts	solvent: acetonitrile–water (12.5:87.5); MP: water–methanol (95:5) with 0.1% formic acid and water–methanol (50:50) (gradient), FR: 0.4 mL/min; column: Acquity UPLC BEH C_18_ (Waters, Milford, CT, USA) (50 × 2.1 mm) with 1.7 μm particles; t_R_: 1.58 min; temperature: 300 °C; detection: *m*/*z* 174.8, 130.9; ESI; positive ion mode	2.0 × 10^−3^–0.2	0.9978	2.1 × 10^−3 1^	1.9–3.1	102.5–103.2	[139]
LC-MS/MS	coconut	solvent: acetonitrile–water; MP: water–methanol (98:2) and methanol, both with 0.1% formic acid and 5 mM ammonium formate (gradient), FR: 0.225 mL/min; column: Acquity UPLC BEH C_18_ (Waters, Milford, CT, USA) (100 × 2.1 mm) with 1.7 μm particles; t_R_: 3.45 min; temperature: 500 °C; detection: *m*/*z* 175.2, 131.2; ESI; positive ion mode	2.5 × 10^−3^–2.5 × 10^−1^	≥0.9984	3.0 × 10^−3 1^	1.0–7.0	84.0–100.0	[140]
LC-MS/MS	milk	solvent: acetonitrile; MP: 0.1% formic acid and acetonitrile with 0.1% formic acid (gradient), FR: 0.2–0.4 mL/min; column: Waters Xterra C_18_ (Waters, Wien, Austria) (50 × 3.0 mm) with 3.5 μm particles; t_R_: 5.22 min; temperature: 400 °C; detection: *m*/*z* 175.2, 64.9; ESI; positive ion mode	5.0 × 10^−2^–1.0 ^1^	>0.9900	1.7 × 10^−2 1^	5.7–10.9	83.0–89.0	[141]
LC-MS/MS	sweet green pepper	solvent: methanol; MP: 0.1% formic acid and methanol (gradient), FR: 0.3 mL/min; column: Waters Nova-Pak C_18_ (Waters, Wien, Austria) (150 × 3.9 mm) with a guard column (20 × 3.9), both with 4 μm particles; t_R_: 8.5 min; temperature: 400 °C; detection: *m*/*z* 174.8, 130.6; ESI; positive ion mode	0.2–2.4 ^1^	0.9954	-	-	71.0–107.0	[142]
LC-MS/MS	sewage sludge	solvent: methanol; MP: acetonitrile and 100 mM ammonium formate (gradient), FR: 0.2 mL/min; column: Waters Atlantis HILIC (Waters, Wien, Austria) (50 × 4.6 mm) with 3 μm particles; ESI; positive and negative ion mode	1.3 × 10^−3^–2.5 ^1^	0.9966	-	3.0–9.0	48.0–95.0	[143]
LC-MS/MS	freshwater fatty fish muscle tissue	solvent: acetonitrile; MP: 0.1% formic acid and acetonitrile (gradient), FR: 0.6 mL/min; column: Zorbax Eclipse XDB-C_18_ (Agilent, Santa Clara, CA, USA) (150 × 4.6 mm) with 5 μm particles; t_R_: 9.9, 10.2 min; detection: *m*/*z* 175.1, 131.1	-	>0.9900	-	2.0–9.0	88.0–107.0	[144]
LC-MS/MS	wastewater	solvent: acetonitrile–water (10:90); MP: 0.1% formic acid and methanol (gradient), FR: 0.5 mL/min; column: Phenomenex Kinetex C_18_ (Phenomenex, Torrance, CA, USA) (150 × 4.6 mm) with 2.6 μm particles; t_R_: 7.26 min; temperature: 500 °C; detection: *m*/*z* 175.1, 131.1; ESI; positive ion mode	1.0 × 10^−5^–1.0 × 10^−3^	0.9956	-	6.0–8.0	79.0–102.0	[145]
LC-MS/MS	orange	solvent: acetonitrile; MP: 10 mM ammonium formate buffer pH 4-methanol (10:90), FR: 0.6 mL/min; column: Zorbax Eclipse plus C_18_ (Agilent, Santa Clara, CA, USA) (150 × 4.6 mm) with 5 μm particles; t_R_: 2.66 min; temperature: 450 °C; detection: *m*/*z* 175.02; ESI; positive ion mode	5.0 × 10^−3^–0.2 ^1^	0.9998	1.3 × 10^−3 1^	5.0–5.4	102.9	[146]
LC-MS/MS	vegetable oil	solvent: methanol–water (10:90); MP: acetonitrile and 2 mM ammonium acetate (gradient), FR: 0.5 mL/min; column: Waters Acquity UPLC BEH C_18_ (Waters, Milford, CT, USA) (100 × 2.1 mm) with 1.7 μm particles; t_R_: 3.22 min; temperature: 550 °C; detection: *m*/*z* 175.0, 131.0; ESI; positive ion mode	5.0 × 10^−3^–0.5 ^1^	0.9957	1.0 × 10^−3 1^	1.9–10.9	95.3–106.2	[147]
HPLC	milk	solvent: acetonitrile–sodium 1-octanesulphonate (1:1), pH 3.5; MP: 0.01 M sodium 1-octanesulphonate, pH = 3.5, acetonitrile–water (gradient); FR: 0.5 mL/min; column: Alltima C_18_ (Restek, Rheinfelden, Switzerland) (150 × 3.2 mm) with 5 μm particles and 100 Å pore size and a guard column (7.5 × 3.2 mm); temperature: 35 °C; detection: UV PDA, 294 nm	4.3 × 10^−3^–1.0	0.9999	3.4 × 10^−3^	97.9–102.2	77.0–80.0	[156]
HPLC	livestock, milk	solvent: acetonitrile– methanol–0.02M sodium dihydrogen phosphate (2:2:6); MP: acetonitrile–methanol–0.02 M NaH_2_PO_4_ (gradient); FR: 1.0 mL/min; column: Cosmosil 5 C_18_ MS-II (Nacalai Tesque, Kyoto, Japan) (250 × 4.6 mm) with 5 µm particles; detection: UV PDA (290–320 nm) and fluorescence (Ex: 290 nm, Em: 320 nm)	5.0 × 10^−2^–2.5	1.0	2.0 × 10^−2^ (UV) 5.0 × 10^−3^ (fluorescence)	-	84.5–97.6	[157]
HPLC	lemon	solvent: 0.5% trifluoroacetic acid in acetonitrile; MP: acetonitrile–water–30% ammonia solution (39:60.5:0.5); FR: 1.0 mL/min; column: Supelcosil LC-18 RP column (Sigma-Aldrich, St. Louis, MO, USA) (250 × 4.6 mm) with 5 μm particles and 100 Å pore size; t_R_: 5.8 min; room temperature; detection: UV, max. absorption: 254 nm	0.1–40.0	0.9990	2.7 × 10^−1^	3.8–5.0	96.7–98.3	[158]
HPLC	water	solvent: methanol–water (50:50); MP: methanol–water (40:60) with 0.6% ammonia; FR: 1.0 mL/min; column: Nova-Pack C_18_ (Waters, Wien, Austria) (150 × 3.9 mm) with a Nova Pack Silica guard column (20 × 3.9), both with 4 μm particles; t_R_: 6.2 min; room temperature; detection: fluorescence, Ex: 300 nm, Em: 350 nm	1.0 × 10^−3^–0.1	0.9980	0.8 × 10^−4^–1.3 × 10^−4^	2.3–4.3	86.0–89.0	[159]
HPLC	wine	solvent: methanol; MP: acetonitrile and 10 mM ammonium acetate (gradient); FR: 1.0 mL/min; column: Synergy Max-RP C_12_ (Phenomenex, Torrance, CA, USA) (250 × 4.6 mm) with 4 µm particles and a guard column Security Guard Max-RP C_12_ (4.0 mm × 3.0 mm); temperature: 35 °C; vaporization temperature: 300 °C; detection: *m*/*z* 202; APCI; positive ion mode	-	0.9900	8.0 × 10^−3^	<10	94.5–102.4	[160]
HPLC	fruit	solvent: acetonitrile; MP methanol and acetic acid (gradient); FR: 1.0 mL/min; column: polymer particles (50 × 4.6 mm); t_R_: 7.5 min; temperature: 0–180 °C; detection: fluorescence, Ex: 305 nm, Em: 345 nm	0.1–5.0	0.9990	3 × 10^−5^	4.2–7.9	88.7–102.4	[161]
HPLC	water	solvent: methanol; MP: methanol–water (45:55); FR: 1.0 mL/min; column: Symmetry C_18_ (Waters, Wien, Austria) (150 × 3.9 mm) with 8 µm particles; t_R_: 3.5 min; temperature: 30 °C; detection: fluorescence, Ex: 300 nm, Em: 350 nm	5.0 × 10^−4^–0.3	0.9990	4.0 × 10^−5^	0.2–3.3	96.9–115.7	[162]
HPLC	apple	solvent: MP; MP: methanol–water (50:50); FR: 1.0 mL/min; column: Symmetry C_18_ (Waters, Wien, Austria) (150 × 3.9 mm) with 5 µm particles; t_R_: <6 min; temperature: 20 °C; detection: fluorescence, Ex: 280 nm, Em: 315 nm	1.0 × 10^−2^–1.0 ^1^	0.9998	3.0 × 10^−3 1^	3.8–4.1	92.3–96.1	[163]
HPLC	water, soil	solvent: methanol; MP: methanol–water (60:40), pH 7; FR: 1.0 mL/min; column: Centurysil C_18_ (Macherey-Nagel, Düren, Ger-many) (250 × 4.6 mm) with 5 µm particles; t_R_: <8.0 min; room temperature; detection: fluorescence, Ex: 280 nm, Em: 315 nm	5.0 × 10^−3^–0.8 (water) 1.0 × 10^−2^–1.0 ^1^ (soil)	0.9997 (water) 0.9994 (soil)	5.0 × 10^−4^–1.0 × 10^−3^ (water) 1.0 × 10^−3^–1.6 × 10^−3 1^ (soil)	3.1–5.8 (water) 4.2–6.2 (soil)	86.0–94.0 (water) 84.0–90.8 (soil)	[164]
HPLC	orange juice	solvent: toluene; MP: methanol–water (70:30); FR: 0.6 mL/min; column: Zorbax Eclipse XDB-C_8_ (Agilent, Santa Clara, CA, USA) (150 × 4.6 mm) with 5 µm particles; t_R_: <3.0 min; detection: fluorescence (Ex: 305 nm, Em: 345) nm and UV (max. absorption: 290 nm)	1.0 × 10^−2^–5.0	>0.9950	4.0 × 10^−3^	6.6–7.6	2.1–15.1	[165]
HPLC	soil	solvent: acetonitrile–10 mM phosphate buffer, pH 8.7 (59:41); MP: acetonitrile–10 mM phosphate buffer pH 8.7 (22:78); FR: 1.0 mL/min; column: Nova-Pak C_18_ (Waters, Wien, Austria) (150 × 3.9 mm) with a Guard-Pak C_18_ guard column, both with 4 μm particles; t_R_: 4.65 min; room temperature; detection: fluorescence, Ex: 305 nm, Em: 335 nm	5.0 × 10^−3^–5.0	0.9970–0.9990	1.2 × 10^−6^–2.8 × 10^−6 1^	-	80.0–117.0	[166]
HPLC	tomato	solvent: acetonitrile; MP: acetonitrile–water (60:40); column: OptimaPak C_18_ (R Stech, Daejeon, South Korea) (4.6 × 150 mm) with 5 µm particles; t_R_: <11 min; detection: UV, max. absorption: 245 nm	5.0 × 10^−3^–0.2 ^1^	0.9990	2.4 × 10^−4 1^	4.5–5.3	76.0–89.0	[167]
HPLC	edible fungi	solvent: methanol–dichloromethane (5:95); MP: methanol–water (35:65); FR: 1.0 mL/min; column: Centurysil C_18_ (Macherey-Nagel, Düren, Germany) (250 × 4.6 mm) with 5 µm particles; temperature: 30 °C; detection: UV, max. absorption: 275 nm	5.0 × 10^−2^–5.0	0.9993	3.0 × 10^−3 1^	0.5–3.5	86.0–105.0	[168]
HPLC	fruit, vegetables	solvent: MP; MP: methanol–water (50:50); FR: 1.0 mL/min; column: Agilent Sorbax SB C_18_ (Agilent, Santa Clara, CA, USA) (150 × 4.6 mm) with 5 µm particles; t_R_: 5.5 min; detection: UV diode array, max. absorption: 280 nm	2.7 × 10^−3^–2.0	0.9894	9.0 × 10^−4^	-	-	[169]
HPLC	tissue	solvent: 1-octanol; MP: methanol and 0.1% acetic acid (gradient); FR: 1.0 mL/min; column: Atlantis dC_18_ (Waters, Wien, Austria) (150 × 4.6 mm) with 5 µm particles; t_R_: 3.3 min; room temperature; detection: UV, max. absorption: 296 nm	5.0 × 10^−3^–1.0	0.9991–0.9995	3.0 × 10^−4^	1.4–3.1	89.0–105.0	[170]
HPLC	milk	solvent: 1-octanol; MP: 0.1% acetic acid and methanol (gradient); FR: 1.0 mL/min; column: Atlantis dC_18_ (Waters, Wien, Austria) (150 × 4.6 mm) with 5 µm particles; t_R_: <3 min; room; detection: UV, max. absorption: 296 nm	5.0 × 10^−3^–1.0	0.9990	5.0 × 10^−3^	1.2–2.8	89.0–105.0	[171]
HPLC	orange	solvent: acetonitrile–acetic acid (99:1); MP: 5 mM ammonium formate and methanol containing 5 mM ammonium formate (gradient); FR: 0.5 mL/min; column: Inertsil ODS-4 (GL Sciences, Tokyo, Japan) (50 × 2.1 mm) with 3 µm particles and a guard column (4 mm × 2 mm) with 4 µm particles; t_R_: 7.0 min; temperature: 30 °C; source temperature: 500 °C; detection: *m*/*z* 175.1; ESI; positive ion mode	0.1–2.5 × 10^−1 1^	0.9983	2.0 × 10^−3 1^	6.9–12.2	93.1–92.7	[172]
HPLC	wastewater, fruit juice	solvent: methanol; MP: methanol–water (50:50); FR: 1.0 mL/min; column: Zorbax SB-C_18_ (Agilent, Santa Clara, CA, USA) (150 × 4.6 mm) with 5 µm particles; t_R_: 2.2 min; temperature: 30 °C; detection: UV diode array, max. absorption: 298 nm	1.0 × 10^−2^–1.0	0.9962	2.6 × 10^−3^	6.5	92.9–103.9	[173]
HPLC	milk, egg, tissue, feed, water, soil	solvent: acetonitrile–HCOOH; MP: 0.1% HCOOH-5 mM HCOONH_4–_0.1% HCOOH (gradient); FR: 0.3 mL/min; column: Acclaim 120 C_18_ (Termo Fisher Scientific, Waltham, MA, USA) (150 × 2.1 mm) with 2.2 µm particles; t_R_: 7.2 min; temperature: 40 °C; source temperature: 250 °C; detection: *m*/*z* 202.04; ESI; positive ion mode	1.0 × 10^−6^–0.2 ^1^	-	1.0 × 10^−3^	5.0	-	[174]
HPLC	lemon, cucumber, apple	solvent: MP; MP: water–methanol (50:50); FR: 1.0 mL/min; column: Zorbax Eclipse XDB-C_18_ (Agilent, Santa Clara, CA, USA) (150 × 4.6 mm) with 5 µm particles; t_R_ < 5 min; room temperature; detection: UV diode array, max. absorption: 285 nm	2.0 × 10^−3^–0.5	0.9978	5.4 × 10^−4^	1.3–3.4	96.9–101.3	[175]
HPLC	TBZ solution	solvent: methanol–water (30:70); MP: 1-octanesulfonic acid sodium salt–0.1% methanesulfonic acid and acetonitrile (gradient); FR: 1.5 mL/min; column: ACE 5 C_18_ (Advanced Chromatography Technologies, Aberdeen, Scotland) (50 × 4.6 mm) with 5 µm particles; t_R_: <1.5 min; temperature: 35 °C; detection: UV, max. absorption: 300 nm	0.1–120.0	1.0	-	1.3–5.4	94.6–99.5	[176]
HPLC	fruit, vegetables	solvent: acetonitrile–water (40:60); MP: acetonitrile–ammonium acetate (40:60); FR: 1.0 mL/min; column: Hisep 5 C_18_ (Sigma-Aldrich, Darmstadt, Germany) (250 × 4.6 mm) with 5 µm particles; t_R_: <10 min; temperature: 40 °C; detection: fluorescence, Ex: 285 nm, Em: 315 nm	2.0 × 10^−3^–1.0	0.9998	7.5 × 10^−3 1^	1.3–5.4	80.0–99.1	[177]
HPLC	citrus fruit	solvent: acetonitrile; MP: water and methanol (gradient); FR: 1.0 mL/min; column: Kromasil dC_18_ (Eka Chemicals, Bohus, Sweden) (150 × 4.6 mm) with 5 µm particles; t_R_: <7 min; temperature: 25 °C; detection: fluorescence, Ex: 280 nm, Em: 315 nm	0.3–3.0 ^1^	-	0.2 ^1^	<8.0	28.0–38.0	[178]
HPLC	citrus fruit	solvent: methanol; MP: water and acetonitrile (gradient); FR: 0.7 mL/min; column: Symmetry C_18_ (Waters, Wien, Austria) (150 × 3.0 mm) with 3.5 µm particles and a guard cartridge Atlantis T3 with 3 µm particles; t_R_: <4 min; detection: UV diode array, max. absorption: 286 nm, 300 nm	2.0 × 10^−5^–2.0 × 10^−3^	0.9980	4.0 × 10^−3 1^ (orange) 9.0 × 10^−3 1^ (lemon)	<4.0	5.1 (orange) 6.1 (lemon)	[179]
HPLC	orange	solvent: MP; MP: 1 × 10^−3^ M phosphate buffer (pH 7) and methanol (gradient); FR: 0.6 mL/min; column: Zorbax Eclipse XDB-C_8_ (Agilent, Santa Clara, CA, USA) (150 × 4.6 mm) with 5 µm particles and a guard cartridge Atlantis T3 with 3 µm particles; t_R_: <8 min; detection: UV diode array, max. absorption: 280 nm, 300 nm	-	0.9930	0.1 ^1^	6.0	21.0	[180]
HPLC	citrus fruit	solvent: acetonitrile; MP: 1 × 10^−2^ M acetonitrile–ammonium acetate (35:65); FR: 1.0 mL/min; column: s Epic C_18_ (Termo Fisher Scientific, Waltham, United States) (150 × 4.6 mm) with 5 µm particles; t_R_: <8 min; room temperature; detection: fluorescence, Ex: 305 nm, Em: 350 nm	1.0 × 10^−2^–1.0	0.9990	7.2 × 10^−4^	2.6–6.9	84.9–98.4	[181]
HPLC	red grape, cherry tomato	solvent: methanol; MP: methanol–water (61:39); FR: 0.8 mL/min; column: Thermo Scientific C_18_ (Termo Fisher Scientific, Waltham, United States) (250 × 4.6 mm) with 5 µm particles; detection: UV, 200–370 nm	-	0.9994	0.03	3.2	99.3	[182]
HPLC	Chinese herbs	solvent: acetonitrile; MP: 0.05% formic acid in methanol, 0.05% formic acid and 5 mM ammonium formate in water (gradient); FR: 0.4 mL/min; column: Agilent Poroshell 120 EC-C_18_ (Agilent, Santa Clara, CA, USA) (150 × 3.0 mm) with 2.7 µm particles; t_R_: 5.8 min; temperature: 35 °C; source temperature: 500 °C; detection: *m*/*z* 202.2; ESI; positive ion mode	2.0 × 10^−3^–0.4 ^1^	0.9997	-	2.3–6.2	86.3–99.7	[183]
HPLC	fruit, vegetables, juice	solvent: 0.1 M hydrochloric acid; MP: phosphate buffer–acetonitrile (80:20) adjusted to pH 4; FR: 0.25 mL/min; column: Agilent SB-C_18_ (Agilent, Santa Clara, CA, USA) (100 × 2.2 mm); t_R_: <13 min; detection: UV, max. absorption: 285 nm	4.7 × 10^−3^–1.0 ^1^	0.9930–0.9980	1.8 × 10^−3^–6.1 × 10^−3 1^	3.1–10.6	59.3–111.4	[184]
HPLC	specific parts of honeybees	solvent: acetonitrile; MP: water and acetonitrile (gradient); FR: 200 nL/min; column: EASY-Spray PepMap C_18_ (Termo Fisher Scientific, Waltham, MA, USA) (150 × 75 µm) with 3 µm particles and 100 Å pores; t_R_: 21 min; temperature: 25 °C; capillary temperature: 250 °C; MS detection, ESI; positive ion mode	-	0.9950	6.0 ^2^	9.9	75	[185]
HPLC	banana, citrus fruit	solvent: methanol; MP: phosphoric acid buffer (pH 7) –acetonitrile–methanol (70:20:10); FR: 1.0 mL/min; column: Shiseido Capcell Pak C_18_ (Shiseido, Tokyo, Japan) (250 × 4.6 mm) with 5 µm particles; t_R_: 12.47 min; temperature: 40 °C; detection: UV photo diode array, max. absorption: 285 nm	3.1 × 10^−1^–20.0	0.9990	9.0 × 10^−3^–1.7 × 10^−2^	1.3	93.6–98.1	[186]
UHPLC-MS/MS	fruit juice	solvent: methanol and dichloromethane; MP: methanol and 0.01% formic acid (gradient), FR: 0.35 mL/min; column; Waters Acquity UPLC BEH C_18_ column (Waters, Milford, CT, USA) (100 × 2.1 mm) with 1.7 µm particles; t_R_: 2.72–2.79 min; vaporization temperature: 350 °C; detection: *m*/*z* 202, 175, 131; ESI; positive ion mode	-	0.9981	1.8 × 10^−3^	5.0–15.1	76.8–87.2	[188]
UHPLC-MS/MS	egg	solvent: methanol–0.05% formic acid (50:50); MP: methanol, 0.05% formic acid, FR: 0.3 mL/min; column: Acquity UPLC BEH C_18_ column (Waters, Milford, CT, USA) (100 × 2.1 mm) with 1.7 µm particles; t_R_: 3.09–3.13 min; vaporization temperature: 350 °C; detection: *m*/*z* 201.8, 175.2, 131.2; ESI; positive ion mode	-	-	0.2 ^1^	9.1–15.2	88.4–97.0	[190]
UHPLC-MS	milk, powdered milk-based infant formulae	solvent: 0.05% formic acid and methanol; MP: 0.05% formic acid and methanol (gradient), FR: 0.3 mL/min; column: Hypersil GOLD aQ C_18_ column (Termo Fisher Scientific, Waltham, MA, USA) (100 × 2.1 mm) with 1.7 µm particles; t_R_: 1.40–1.48 min; detection: *m*/*z* 202.0434, 175.0324, 131.0604; ESI; positive ion mode	4.1 × 10^−3^–8.1 × 10^−3 1^	-	8.1 × 10^−3 1^	-	-	[191]
UHPLC-MS/MS	chicken meat	solvent: 0.1 % formic acid in acetonitrile–water (50:50); MP: 0.1% formic acid in acetonitrile and 0.1% formic acid (gradient), FR: 0.3 mL/min; column: C_18_ column (100 × 2.1 mm) with 1.7 µm particles; t_R_: 1.93–2.13 min; detection: *m*/*z* 201.8, 175.2; ESI; positive ion mode	-	-	6.4 × 10^−3 1^	8.1–20.5	91.1–114.2	[192]
UHPLC-MS/MS	meat-based baby food, milk-based infant formulae	solvent: methanol and 0.05% formic acid; MP: methanol and 0.05% formic acid (gradient); FR: 0.3 mL/min; column: UPLC BEH C_18_ column (Waters, Milford, CT, USA) (100 × 2.1 mm) with 1.7 µm particles; t_R_: 3.05–3.19 min; detection: *m*/*z* 201.8, 175.2; ESI; positive ion mode	5 × 10^−3^–0.1 ^1^	-	5.0 × 10^−4 1^	1.0–4.0	87.2–125.0	[193]
UHPLC-MS/MS	gilthead sea bream	solvent: 0.1% formic acid in acetonitrile and 0.1% formic acid; MP: 0.1% formic acid in acetonitrile and 0.1% formic acid (gradient); FR: 0.3 mL/min; column: BEH C_18_ column (Waters, Milford, CT, USA) (100 × 2.1 mm) with 1.7 µm particles; t_R_: 2.04–2.11 min; detection: *m*/*z* 201.8, 175.2, 131.2; ESI; positive ion mode	-	-	3.0 × 10^−3 1^	6.0–17.0	76.0–97.0	[194]
UHPLC-MS/MS	green tea tablets and capsules	solvent: acetonitrile and 1% acetic acid; MP: acetonitrile and 0.01% formic acid (gradient); FR: 0.35 mL/min; column: Hypersil GOLD aQ (Termo Fisher Scientific, Waltham, MA, USA) (100 × 2.1 mm) with 1.9 µm particles; t_R_: 1.93–2.10 min; detection: *m*/*z* 202, 175, 131	-	>0.9800	1.0 × 10^−3 1^	7.0–16.0	82.0–88.0	[195]
UHPLC-MS/MS	milk	solvent: acetonitrile; MP: 0.01% acetic acid and acetonitrile–water (90:10) (gradient); FR: 0.6 mL/min; column: Acquity stainless steel HSS T3 (Waters, Wien, Austria) (100 × 2.1 mm) with 1.8 µm particles; t_R_: 3.13 min; detection: *m*/*z* 201.9, 130.85, 174.8; ESI; positive ion mode	1.4 × 10^−4^–1.9 × 10^−3 1^	0.9980	0.1 ^1^	-	87.0–101.0	[196]
UHPLC-MS/MS	bovine milk	solvent: 0.1% acetic acid–0.1% formic acid in acetonitrile; MP: 0.1% acetic acid and 0.1% formic acid in acetonitrile (gradient); FR: 0.3 mL/min; column: Acquity BEH C_18_ column (Waters, Milford, CT, USA) (50 × 2.1 mm) with 1.7 µm particles; t_R_: 1.23 min; detection: *m*/*z* 202, 175 and 131; ESI; positive ion mode	-	-	3.0 × 10^−5^	-	93.3–95.0	[197]
UHPLC-MS/MS	chicken tissue (egg, liver, muscle)	solvent: methanol–ammonia (99:1); MP: 5% methanol and 95% water containing 0.1% formic acid (gradient); FR: 0.35 mL/min; column: BEH C_18_ column (Waters, Milford, CT, USA) (100 × 2.1 mm) with 1.7 µm particles; t_R_: 3.27 min; detection: *m*/*z* 202, 175, 131; ESI; positive ion mode	1.0 × 10^−4^–0.1 ^1^	-	0.1 ^1^	3.3–13.7	93.6–116.7	[198]
UHPLC-MS/MS	bovine milk	solvent: 0.1% formic acid with 5 mM ammonium acetate–methanol (90:10); MP: 0.1% formic acid with 5 mM ammonium acetate and methanol (gradient); FR: 0.3 mL/min; column: BHE C_18_ (Waters, Milford, CT, USA) (100 × 2.1 mm) with 1.7 µm particles; detection: *m*/*z* 202.1, 175.1, 131.1; ESI; positive ion mode	1.1 × 10^−2^–1.1 ^1^	0.9991	0.1 ^1^	-	108.0–112.0	[199]
UHPLC-MS	fruit, vegetables	solvent: methanol; MP: 0.1% formic acid in methanol and 0.1% formic acid (gradient); FR: 1.0 mL/min; column: BEH C_18_ column (Waters, Milford, CT, USA) (50 × 2.1 mm) with 1.7 µm particles; t_R_: 1.45 min; detection: *m*/*z* 202.0439; ESI; positive ion mode	1.0 × 10^−2^–0.8	0.9995	3.3 × 10^−3 1^	2.5	108.0	[200]
UHPLC-MS/MS	fruit, vegetables	solvent: ammonium acetate in methanol/water (95:5); MP: 5 mM ammonium acetate in water–methanol (90:10) and 5 mM ammonium acetate in methanol–water (90:10) (gradient); FR: 0.45 mL/min; column: BEH C_18_ column (Waters, Milford, CT, USA) (100 × 2.1 mm) with 1.7 µm particles; t_R_: 4.63 min; detection: *m*/*z* 202, 175.1, 131; ESI; positive ion mode	-	-	-	3.3–10.3	81.0–103.1	[201]
UHPLC-MS/MS	insect bolus	solvent: water–methanol (1:1) containing 10 ng/mL triphenyl phosphate; MP: 0.2% formic acid and 0.2% formic acid–methanol–acetonitrile (gradient); FR: 0.4 mL/min; column: BEH C_18_ column (Waters, Milford, CT, USA) (100 × 2.1 mm) with 1.8 µm particles; t_R_: 3.5 min; detection: *m*/*z* 202, 175, 131; ESI; positive ion mode	1.0 × 10^−4^–3.0 × 10^−2^	-	1.6 × 10^−4 1^	13.5	82.1	[202]
UHPLC-MS	water	solvent: methanol; MP: 2 mM ammonium formate and 2 mM ammonium formate in methanol (gradient); FR: 0.4 mL/min; column: BEH C_18_ column (Waters, Milford, CT, USA) (100 × 2.1 mm) with 1.8 µm particles; t_R_: 5.94 min; detection: *m*/*z* 202.0434; ESI; positive ion mode	0–5.0 × 10^−5 1^	0.9982	1.0 × 10^−5^	7.0	107.6	[203]
UHPLC-MS/MS	bovine milk	solvent: methanol; MP: 0.1% formic acid and 0.1% formic acid in acetonitrile (gradient), FR: 0.4 mL/min; column: BEH C_18_ column (Waters, Milford, CT, USA) (50 × 2.1 mm) with 1.7 µm particles; t_R_: 3.27 min; detection: *m*/*z* 201.9, 175, 131.1; ESI; positive ion mode	0–1.0 × 10^−2 1^	0.9967	3.0 × 10^−6 1^	-	52.6–81.7	[204]
UHPLC-MS/MS	orange juice	solvent: acetonitrile; MP: 0.1% formic acid and 5 mM ammonium formate in (a) water–methanol (98:2) and (b) methanol (gradient); FR: 0.25 mL/min; column: BEH C_18_ column (Waters, Milford, CT, USA) (50 × 2.1 mm) with 1.7 µm particles; t_R_: 3.04 min; detection: *m*/*z* 202; ESI; positive ion mode	-	-	3.0 × 10^−3^	3.0–11.0	80.0–86.0	[205]
UHPLC-MS/MS	vegetables, cereals	solvent: acetonitrile; MP: 0.1% formic acid and 5 mM ammonium formate in water, 0.1 % formic acid and 5 mM ammonium formate in methanol (gradient); FR: 0.25 mL/min; column: BEH C_18_ column (Waters, Milford, CT, USA) (50 × 2.1 mm) with 1.7 µm particles; ESI; positive ion mode	2.0 × 10^−2^–1.6 ^1^	-	1.0 × 10^−2 1^	3.0–13.0	73.0–74.0	[206]
UHPLC-MS/MS	bovine tissue (liver, kidney, muscle)	solvent: solution of trichloroacetic acid; MP: 10 mM ammonium formate in water–acetonitrile (98:2) and 0.1% formic acid in methanol–acetonitrile (75:25) (gradient); FR: 0.4 mL/min; column: Waters Acquity UPLC column (Waters, Wien, Austria) (100 × 2.1 mm) with 1.8 µm particles; ESI; positive and negative ion mode	-	-	0.1 1	2.0–15.0	90.0–109.0	[207]
UHPLC-MS/MS	swine waste lagoon sludge	solvent: 0.2% formic acid in methanol; MP: 0.1% formic acid and 0.1% formic acid in methanol–acetonitrile (20:80) (gradient); FR: 0.4 mL/min; column: BEH C_18_ column (Waters, Milford, CT, USA) (50 × 2.1 mm) with 1.7 µm particles; detection: *m*/*z* 202, 175, 131; ESI; positive ion mode	1.0 × 10^−3^–0.5 ^1^	-	3.0 × 10^−4 1^	-	70.8–83.1	[208]
UHPLC-MS/MS	wastewater	solvent: acetonitrile; MP: 0.1% formic acid and acetonitrile (gradient); FR: 0.4 mL/min; column: Zorbax Eclipse XDB C_18_ (Agilent, Santa Clara, CA, USA) (50 × 4.6 mm) with 1.8 µm particles; ESI; positive ion mode	2.0 × 10^−5^–5.0 × 10^−3^	0.9979	5.0 × 10^−6^	1.0–11.0	101.0–115.0	[209]
UHPLC-MS/MS	bovine muscle tissue	solvent: acetonitrile; MP: 0.1% formic acid–5 mM ammonium formate and 0.1% formic acid in acetonitrile–water (95:5) (gradient); FR: 0.4 mL/min; column: BEH C_18_ column (Waters, Milford, CT, USA) (50 × 2.1 mm) with 1.7 µm particles; t_R_: 2.04 min; detection: *m*/*z* 202, 91.9, 131, 175; ESI; positive ion mode	5.0 × 10^−2^–0.2 ^1^	0.9980	1.4 × 10^−2 1^	2.0–10.5	93.3–102.5	[210]
UHPLC-MS	air	solvent: water–methanol (70:30); MP: 0.1% formic acid–4 mM ammonium formate and 0.1% formic acid–4 mM ammonium formate in methanol (gradient); FR: 0.3 mL/min; column: Hypersil Gold aQ column (Termo Fisher Scientific, Waltham, MA, USA) (100 × 2.1 mm) with 1.9 µm particles; t_R_: 5.97 min; detection: *m*/*z* 202.04334, 175.09788; ESI; positive ion mode	5.0 × 10^−3^–0.2	-	-	0.5–4.0	92.8–99.0	[211]
UHPLC-MS/MS	fish bile	solvent: methanol–water (50:50), dichloromethane; MP: 0.05% formic acid and 5 mM ammonium acetate in a) ultrapure water, b) methanol (gradient); FR: 0.35 mL/min; column: Agilent SB-C_18_ column (Agilent, Santa Clara, CA, USA) (100 × 3 mm) with 1.8 µm particles; t_R_: 3.22 min; detection: *m*/*z* 208.1, 136.1, 180.1; ESI; positive ion mode	1.0 × 10^−4^–2.0 × 10^−2^	0.9995	8.0 × 10^−5^	1.0–4.0	86.0–93.0	[212]
UHPLC-MS	shellfish	solvent: acetonitrile–water (50:50); MP: acetonitrile and water (gradient); FR: 0.2 mL/min; column: Purospher STAR RP-18 end-capped column (Sigma-Aldrich, Darmstadt, Germany) (150 × 2.1 mm) with 2 µm particles; t_R_: 5.1 min; detection: *m*/*z* 202.0433; ESI; positive ion mode	1.0 × 10^−5^–5.0 × 10^−2 1^	-	2.0 × 10^−5 1^	1.8	96.0–107.0	[213]
UHPLC-MS/MS	water	solvent: 0.1% ammonium hydroxide, methanol–acetonitrile–propan-2-ol (1:1:1); MP: 0.1% formic acid and acetonitrile–methanol (2:1) (gradient); FR: 0.4 mL/min; column: Acquity BEH C_18_ (Waters, Milford, CT, USA) (150 × 3 mm) with 1.7 µm particles; t_R_: 6.54 min; ESI; positive ion mode	4.0 × 10^−7^–4.0 × 10^−4^	0.9994	7.7 × 10^−8^	-	94.7–104.4	[214]
UHPLC-MS/MS	urine and blood of livestock and poultry	solvent: dichloromethane; MP: 0.2% formic acid–2 mM ammonium acetate, 0.2% formic acid in methanol (gradient); FR: 0.4 mL/min; column: Agilent Zorbax Eclipse Plus C_18_ (Agilent, Santa Clara, CA, USA) (150 × 3 mm) with 1.8 µm particles; t_R_: 9.15 min; detection: *m*/*z* 202, 175, 131; ESI; positive ion mode	1.0 × 10^−3^–0.1	0.9998	3.0 × 10^−4^	4.0–6.0	84.0–90.0	[215]
UHPLC-MS	meat (bovine, chicken, porcine)	solvent: 0.01% acetic acid and 5 mmol/L ammonium formate in water–acetonitrile (2:1); MP: 0.1% formic acid, 0.1% formic acid in acetonitrile, and 0.1% formic acid in methanol (gradient); column: Phenomenex Luna Omega (Phenomenex, Torrance, CA, USA) (100 × 2.1 mm) with 1.6 µm particles; t_R_: 6.6 min; ESI; positive ion mode	-	-	6.7 × 10^−2 1^	7.9–8.7	90.0–108.0	[216]
UHPLC-MS/MS	water, sediment	solvent: methanol–water (1:1); MP: methanol (10–100%, gradient); FR: 0.4 mL/min; column: BEH C_18_ (Waters, Milford, CT, USA) (100 × 2.1 mm) with 1.7 µm particles; t_R_: 3.19 min; detection: *m*/*z* 202.1, 175, 130.9; ESI; positive ion mode	-	0.9995	2.0 × 10^−2^	1.7–8.9	66.0–93.0	[217]
UHPLC-MS/MS	protein powder	solvent: methanol–water (1:5); MP: 0.1% formic acid–0.5 mM ammonium acetate, 0.1% formic acid in methanol, 2 mM ammonium acetate, and methanol (gradient); FR: 0.25 mL/min; column: Acquity HSS-T3 (Waters, Wien, Austria) (100 × 2.1 mm) with 1.8 µm particles; t_R_: 4.7 min; detection: *m*/*z* 202.1, 175, 131; ESI; positive ion mode	-	0.9970	-	1.3–3.2	85.6–111.3	[218]
UHPLC-MS/MS	river sediment	solvent: methanol–ultrapure water; MP: water and methanol (gradient); FR: 0.2 mL/min; column: Kinetex XB-C_18_ (Phenomenex, Torrance, CA, USA) (50 × 2.1 mm) with 1.7 µm particles; t_R_: 1.19 min; detection: *m*/*z* 202.1, 175, 131; ESI; positive ion mode	7.0 × 10^−4^–1.0 ^1^	0.9775	2.0 × 10^−4 1^	6.0–20.0	63.0–70.0	[219]
UHPLC-MS/MS	salmon	solvent: water–acetonitrile; MP: 0.1% formic acid and 0.1% formic acid in acetonitrile (gradient), FR: 0.4 mL/min; column: Zorbax RRHD Eclipse Plus C_18_ (Agilent, Santa Clara, CA, USA) (100 × 2.1 mm) with 1.8 µm particles; t_R_: 3.1 min; ESI; positive ion mode	-	-	-	10.0	96.0	[220]
MLC	wastewater	solvent: water; MP: 0.07 mol/L SDS, 5% pentan-1-ol, and 0.01 M phosphate buffer; FR: 0.95–1.05 mL/min; column: Kromasil C_18_ (Eka Chemicals, Bohus, Sweden) (150 × 4.6 mm) with 5 µm particles; t_R_: 3.5 min; detection: UV/Vis; max. absorption: 305 nm	0.5–15	0.9999	0.2	1.3–8.1	93.9–103.7	[222]
MLC	wastewater	solvent: water; MP: 0.1 mol/L SDS, 6% pentan-1-ol and 0.01 M phosphate buffer; FR: 1 mL/min; column: Kromasil C_18_ (Eka Chemicals, Bohus, Sweden) (150 × 4.6 mm) with 5 µm particles; detection: UV/Vis; max. absorption: 305 nm	1.0 × 10^−2^–2.0	0.9995	5.0 × 10^−3^	-	92.1–104.2	[223]
MEKC	grape, lettuce, orange, tomato	solvent: hydrochloric acid; buffer: 4 mM borate (pH 9.2) containing sodium cholate; MP: methanol–electrophoretic buffer (20:80); capillary: fused-silica (50 cm effective length × 75 µm inner diameter); t_m_: 11.6 min; temperature: 25 °C; electrokinetic injection: 5 s at 10 kV; detection: α-diode array, max. absorption: 210 nm; separation voltage: 15 kV	1.0–100.0	0.9960	0.1–1.0 ^1^	4.0–10.0	53.0–56.0	[224]
MEKC	soil	solvent: water; electrolyte: 15 mM sodium tetraborate, 30 mM SDS, 10 mM tetrabutylammonium phosphate, and 10% acetonitrile; capillary: fused-silica (50 cm effective length × 75 µm inner diameter); t_m_: <7 min; temperature: 20 °C; voltage: 25 kV; detection: UV, max. absorption: 190 nm; separation voltage: 25 kV	2.5 × 10^−1^–5.0	≥0.9970	5.7 × 10^−2 1^	<10.0	28.0–46.0	[225]
CEC	lemon, orange	solvent: acetonitrile; MP: acetonitrile and 0.02 M sodium dihydrogen phosphate; column: long fused-silica capillary with UV-transparent protecting polymer coating (150 mm, 100 µm I.D.) with TBZ imprinted monolith; 60 °C; t_R_: <6 min; detection: UV/Vis, max. absorption: 305 nm	5.0 × 10^−2^–20.0	0.9940–0.9998	4.0 × 10^−2^–4.5 × 10^−2^	4.1–6.9	85.0–105.0	[227]

Ex = excitation; Em = emission; FR = flow rate; MP = mobile phase; t_R_ = retention time; t_m_ = migration time; SDS = sodium dodecyl sulfate. ^1^ Measuring unit is µg/g ^2^ Measuring unit is pg/bee.

**Table 4:** Other analytical methods for the determination of TBZ.

Method	Sample	Conditions and Characteristic Method Parameters	Measuring Range (μg/mL)	Correlation Coefficient	LOD (μg/mL)	RSD (%)	Recovery (%)	Reference
CE	fruit, vegetables	solvent: dichloromethane; capillary column: fused-silica capillary (150 cm × 75 µm); detection: *m*/*z* 202; ESI; positive ion mode	1.0–10.0	0.9889	1.0 × 10^−2^	7.0–12.0	61.0–68.0	[230]
CE	swine muscle, swine liver	solvent: acetonitrile, trifluoroacetic acid; type: CZE; capillary column: fused-silica capillary (75.5 cm × 75 µm); detection: UV/Vis; max. absorption: 292 nm	5.0 × 10^−2^–2.0 ^1^	0.9972 (muscle) 0.9929 (liver)	1.0 × 10^−3 1^	0.3–7.4	87.9–104.9	[231]
CE	fruit, fruit juice	solvent: tetrahydrofurane, chloroform; type: NACE; capillary column: fused-silica capillary (60 cm × 75 µm); detection: UV/Vis; max. absorption: 204 nm	2.5 × 10^−2^–0.5	0.9991	5.1 × 10^−4^	1.4–3.2	79.9–101.1	[232]
CE	water	solvent: 1 M hydrochloric acid; capillary column: bare silica capillary (60 cm × 50 µm); detection: UV/Vis; max. absorption: 210 nm	3.7 × 10^−3^–0.5	0.9952	1.1 × 10^−3^	2.8	96.0–103.0	[233]
CE	meat	solvent: acetonitrile–water (30:70); type: CZE; capillary column: fused-silica capillary (100 cm × 50 µm); detection: *m*/*z* 202; ESI; positive ion mode	4.0 × 10^−3^–0.1 ^1^	0.9987	1.0 × 10^−3 1^	2.8–8.5	76.7–87.7	[234]
voltammetry	TBZ solution	solvent: methanol–formic acid (95:5); buffer: phosphoric acid–glacial acetic acid–boric acid; pH 2.55–9.54; type: SWV; WE: glassy carbon rotating-disk electrode modified with poly(3-methylthiophene); RE: Ag/AgCl; AE: platinum wire; potential range: −4.0–4.0 V	-	0.9913	6.8 × 10^−2^	1.9	-	[236]
voltammetry	orange, apple, pear	solvent: ethanol; buffer: phosphate (pH 6); type: CV; WE: MWCNT-COOH glassy carbon; RE: saturated calomel; AE: platinum wire; scan rate: 0.05–0.2 V	-	0.9950	6.0 × 10^−2^	4.2	95.0–105.0	[237]
voltammetry	apple, leek, tomato	solvent: ethanol; buffer: phosphate (pH 7); type: DPV; WE: glassy carbon modified with ZnFe_2_O_4_/SWCNTs; RE: Ag/AgCl; AE: platinum wire; potential range: 0.2–1.0 V	0.1–20.1	0.9970	1.0 × 10^−2^	3.3	88.0–104.0	[238]
voltammetry	mango, sugar cane, river water, pharmaceutical formulation	solvent: ethanol; buffer: Britton–Robinson (2.2–12); type: SWV; WE: boron-doped diamond; RE: Ag/AgCl; AE: platinum wire; scan rate: 10–150 mVs^−1^	0.1–2.3	0.9996	2.6 × 10^−2^	0.9–5.3	71.0–103.3	[239]
potentiometry	banana	solvent: water, pH 4 with HCl; ISE: liquid membrane (ion-pair: (TBZH_2_)_3_(PMo_12_O_40_)_2_; plasticizer: tricresyl phosphate; PVC; solvent: cyclohexanone); reference electrode: Ag/AgCl; slope: 30 mV/decade; response time: 2–3 min (low *c*), 40–50 s (high *c*); life time: 2 months	2.0–2012.5	-	-	-	99.0–99.6	[240]
potentiometry	orange, lemon, banana	solvent: water, pH 2.6 with HCl; ISE: liquid membrane (ion-pair: TBZ and 5-sulfosalicylate (1%); plasticizer: dibutyl sebacate; PVC; solvent: tetrahydrofuran); reference electrode: Ag/AgCl; slope: 62.2 mV/decade; response time: 8 s; life time: 1 month	0.2–201.3	0.9990	6.4 × 10^−2^	-	102.4–107.0	[242]
potentiometry	orange, lemon, banana, clementine, lime	solvent: water, pH 2.6 with HCl; solid-state ISE: liquid membrane (ion-pair: MWCNT-OSO_3_^−^TBZ^+^ (1%); plasticizer: dibutyl sebacate; PVC; solvent: tetrahydrofuran); reference electrode: Ag/AgCl; slope: 60.4 mV/decade; response time: 8 s; life time: 3 months	0.1–201.3	0.9991	1.2 × 10^−1^	-	95.1–110.0	[243]
immunoassay	fruit juice	conjugate: TBZ-ovalbumin; label: carbon nanoparticles; detection complex: carbon-labelled secondary antibodies and anti-TBZ antibodies; TBZ detection: visual and scanning densitometry	1.1 × 10^−4^–4.1 × 10^−3^	-	8.0 × 10^−5^	-	81.9–123.6	[248]
immunoassay	orange	conjugate: TBZ hapten-BSA; SPR biosensor using gold surfaces; TBZ detection: SPR: polarized light: 670 nm, flow speed: 20 µL/min	2.4 × 10^−4^–1.7 × 10^−3^	-	1.3 × 10^−4^	-	105.1–110.9	[4]
immunoassay	red and white wine	enzyme: HRP; conjugate: TBZ-BSA; absorption: 405 nm	1.0 × 10^−5^–2.0 × 10^−3^	0.9985	5.0 × 10^−6^	-	90.6–108.0	[7]

WE = working electrode; RE = reference electrode; AE = auxiliary electrode. ^1^ Measuring unit is µg/g.

## Supplementary Materials

The following supporting information can be downloaded at: https://www.mdpi.com/article/10.3390/molecules28093926/s1, Figure S1: Raman spectrum of solid TBZ powder [57]; Figure S2: Fluorescence emission spectrum of TBZ (Ex: 299 nm) in a pH 2 buffer aqueous solution [63]; Figure S3: MS-MS spectrum of TBZ [113].
