# Supplementary material for: Review of Characteristics and Analytical Methods for Determination of Thiabendazole
Source: Molecules. 2023 May 6;28(9):3926. doi: 10.3390/molecules28093926 (PMC10179875; doi:10.3390/molecules28093926)
Supplement: Supplementary file 1 [file molecules-28-03926-s001.zip › molecules-2344227-supplementary.pdf]

Review Paper

Review of characteristics and analytical methods for determination of thiabendazole

Mateja Budetić, Doris Kopf, Andrea Dandić and Mirela Samardžić *

Department of Chemistry, Josip Juraj Strossmayer University of Osijek, Osijek, 31000, Croatia

* Correspondence: mirelas@kemija.unios.hr

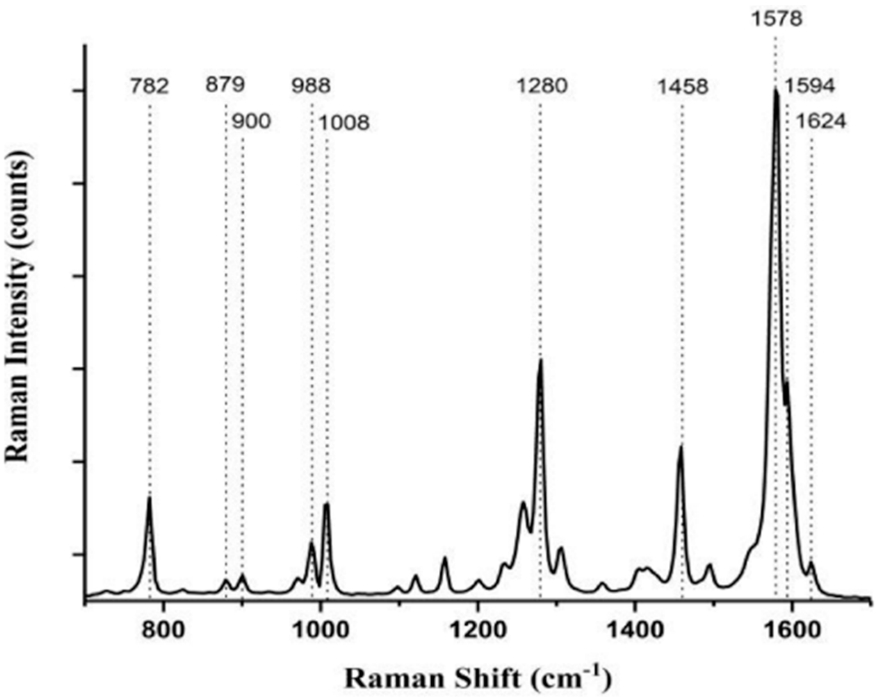

Figure S1. Raman spectrum of solid TBZ powder [57].

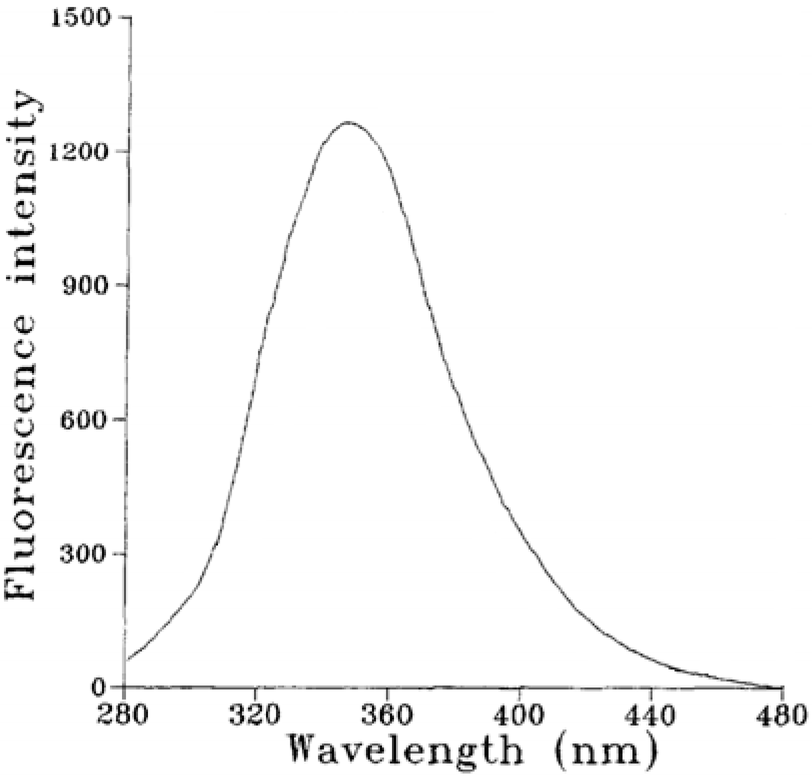

Figure S2. Fluorescence emission spectrum of TBZ (Ex: 299 nm) in a pH 2 buffer aqueous solution [63].

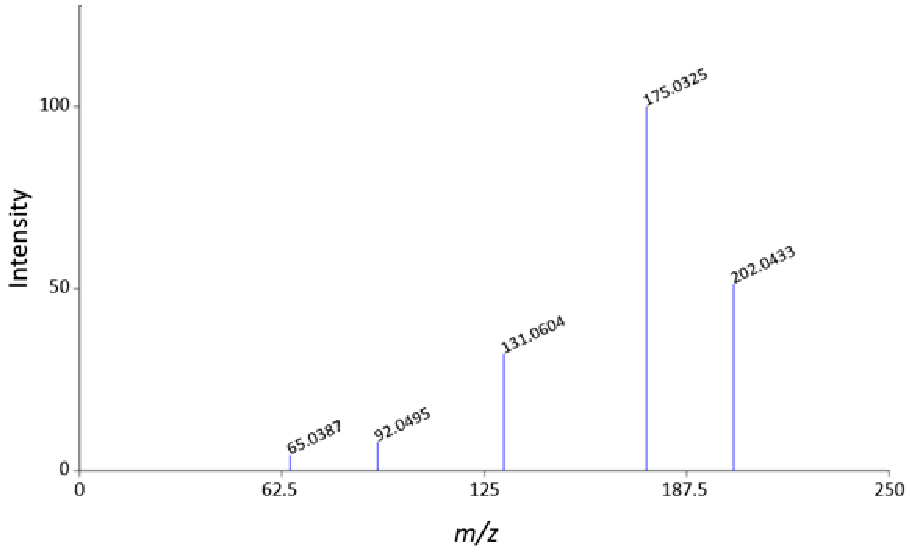

Figure S3. MS-MS spectrum of TBZ [113].

References

57. Chen, Z.; Sun, Y.; Shi, J.; Zhang, W.; Zhang, X.; Huang, X.; Zou, X.; Li, Z.; Wei, R. Facile Synthesis of Au@Ag Core–Shell Nanorod with Bimetallic Synergistic Effect for SERS Detection of Thiabendazole in Fruit Juice. *Food Chem.* **2022**, *370*, 131276, doi:10.1016/j.foodchem.2021.131276.

63. Garcia, L.F.; Aaron, J.J. Flow Injection Analysis for the Determination of Thiabendazole and Fuberidazole in Water by Spectrofluorimetry. *Microchim. Acta 1997 1263* **1997**, *126*, 289–294, doi:10.1007/BF01242335.

113. Thiabendazole | C10H7N3S - PubChem Available online: https://pubchem.ncbi.nlm.nih.gov/compound/Thiabendazole (accessed on 27 April 2023).
